# Redox-configurable ambidextrous catalysis: structural and mechanistic insight†
†Electronic supplementary information (ESI) available: Control studies. CCDC 1405638. For ESI and crystallographic data in CIF or other electronic format see DOI: 10.1039/c5sc02144h


**DOI:** 10.1039/c5sc02144h

**Published:** 2015-07-14

**Authors:** Shahab Mortezaei, Noelle R. Catarineu, Xueyou Duan, Chunhua Hu, James W. Canary

**Affiliations:** a Department of Chemistry , New York University , New York , New York 10003 , USA . Email: canary@nyu.edu

## Abstract

A ligand capable of adopting two pseudo-enantiomeric helically chiral states when bound to copper has been applied as an asymmetric catalyst in the Michael addition of malonate substrates to nitrostyrenes. The absolute configuration of the helically chiral ligand is inverted upon oxidation/reduction of the copper center. In this way, the handedness of the Michael addition product (*R*/*S*) can be selected based on the handedness of the catalyst (Λ/Δ). Exciton coupled circular dichroism (ECCD) was used to identify which of the two pseudo-enantiomeric forms the catalyst adopted after reduction/oxidation, with additional support from X-ray crystallographic data. The synthesis of the ligand was achieved in five steps with an overall 61% yield. Enantiomeric excesses of the Michael addition products of up to 72% (*S*) and 70% (*R*) were obtained in acetonitrile. The ability to choose the handedness of the product based on the chiral state of the catalyst has been demonstrated with several different solvents, bases, nitrostyrene/malonate substrates, and prochiral malonate substrates. A combination of molecular modelling, crystal structure and kinetic data suggest that one urea moiety of the catalyst ligand likely binds the nitrostyrene substrate while blocking the *Re* face of the nitrostyrene in the transition state.

## Abstract 2

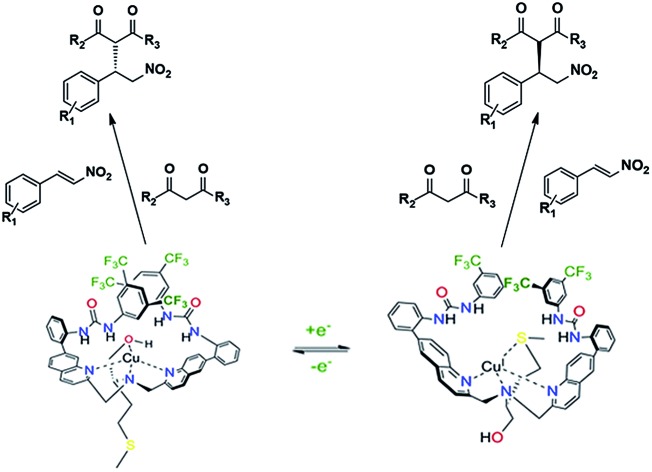
A helically chiral copper complex is used as a switchable asymmetric catalyst capable of delivering either enantiomer of a Michael addition reaction.

## Introduction

The ability to dynamically control catalysts, either *in situ* or before reaction, provides the potential to attain complex target compounds in far fewer steps. The ability of one catalyst to mediate multiple transformations reduces waste and cuts costs associated with other static catalysts. Some initial work in this area has employed catalytic scaffolds such as ferrocenes, strained alkenes, rotaxanes, calixarene, and other building blocks to design dynamic catalysts.^1^–^13^ So far, most designed catalysts tend to use heat, light, or redox state as a means of catalyst control. Scaffolds that are capable of triggered chirality inversion have been employed to design several molecular machines with different functionalities. More recently, our group^14^ and that of Feringa^3^,^14^ have reported catalysts that utilize these helically chiral scaffolds to control the enantioselectivity of asymmetric conjugate addition reactions. Although much progress has been made developing dynamic catalysts, most of these examples have been limited to proof of concept studies. The efficient synthesis and straightforward use of the ambidextrous catalyst lend themselves well to more comprehensive and in-depth studies not usually associated with a dynamic catalyst. Here we provide additional characterization, scope of reaction studies, and mechanistic insight into the asymmetric ambidextrous catalyst.^15^

### Design

**Fig. 1:**
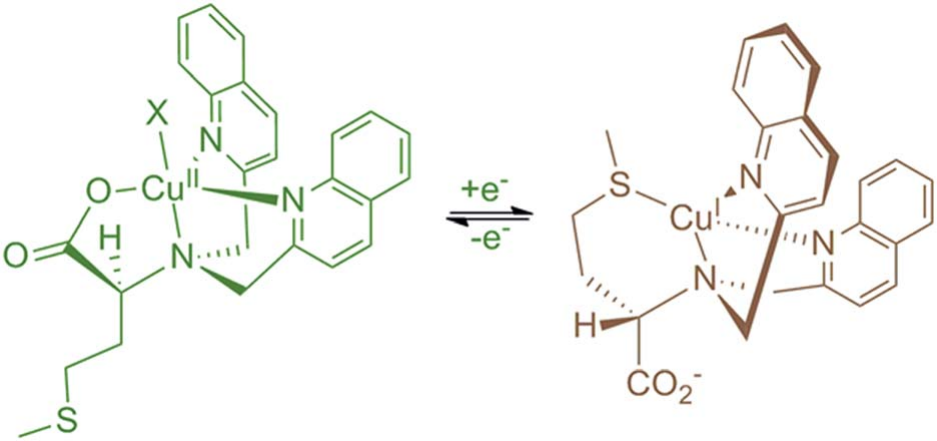
Helical inversion of a methionine-based copper complex.^16^

A copper complex capable of redox-triggered inversion of helical chirality was discovered several years ago (Fig. 1).^16^–^18^ Exciton coupled circular dichroism (ECCD) was used as an optical readout to differentiate between the two possible pseudo-enantiomeric states. Chromophores were attached to the scaffold to monitor ECCD properties at different wavelengths. After establishing that chromophores could be appended to the complex without destroying the readout, we sought to attach a catalytic group to the scaffold in hopes of designing a catalyst that possessed some form of dynamic control.

With a chiral scaffold already in hand, we looked to the field of asymmetric catalysis for inspiration. Several chiral scaffolds have already been appended with either organocatalytic or metal-containing catalytic moieties to create new asymmetric catalysts.^19^–^21^ We reasoned that a catalyst attached to an asymmetric scaffold capable of inverting its chirality would be capable of catalyzing the formation of either enantiomer product depending on the enantiomeric state of the scaffold. Of the organocatalysts discovered, the urea and thiourea subset have proven to be some of the most versatile and robust. (Thio)urea catalysts have been employed in a diverse set of reactions, such as cycloadditions, Michael additions, and condensations.^22^–^24^ The mechanism of each of these reactions most often features the (thio)urea group activating a substrate by hydrogen-bonding in a Lewis acidic interaction.^25^,^26^ The compatibility of urea groups with the chiral scaffold led to their selection for the ambidextrous catalyst.

The asymmetric conjugate addition of malonate esters to nitroalkenes has been performed several times with a diverse array of urea-containing organocatalysts.^27^–^31^ The heavily studied nitrostyrene conjugate addition was chosen as a model reaction for the ambidextrous catalyst.

**Fig. 2:**
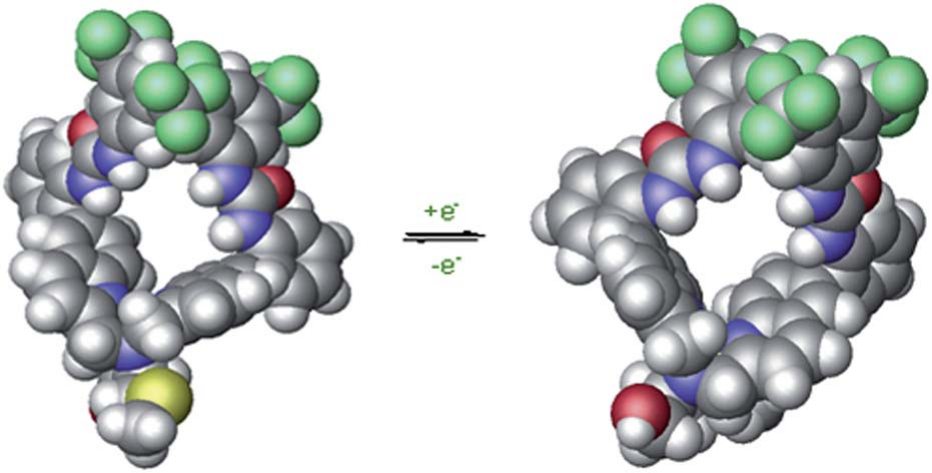
Model used for design of a helically chiral scaffold with appended urea groups.

Molecular modeling studies were used to identify the ideal position for the placement of the catalytic urea group. A model of the catalyst was built using X-ray data from a related complex (Fig. 2).^32^ Placement of the urea groups on the 6-position of the quinoline chromophores appeared to project the urea groups away from the copper scaffold and lower the chance of the urea groups hindering the chirality inversion process. Additionally, a chiral cleft forms from the near perpendicular orientation of the urea groups. We hypothesized this asymmetric pocket could be a potential binding site for prochiral substrates.

## Results and discussion

### Catalyst synthesis

**Scheme 1:**
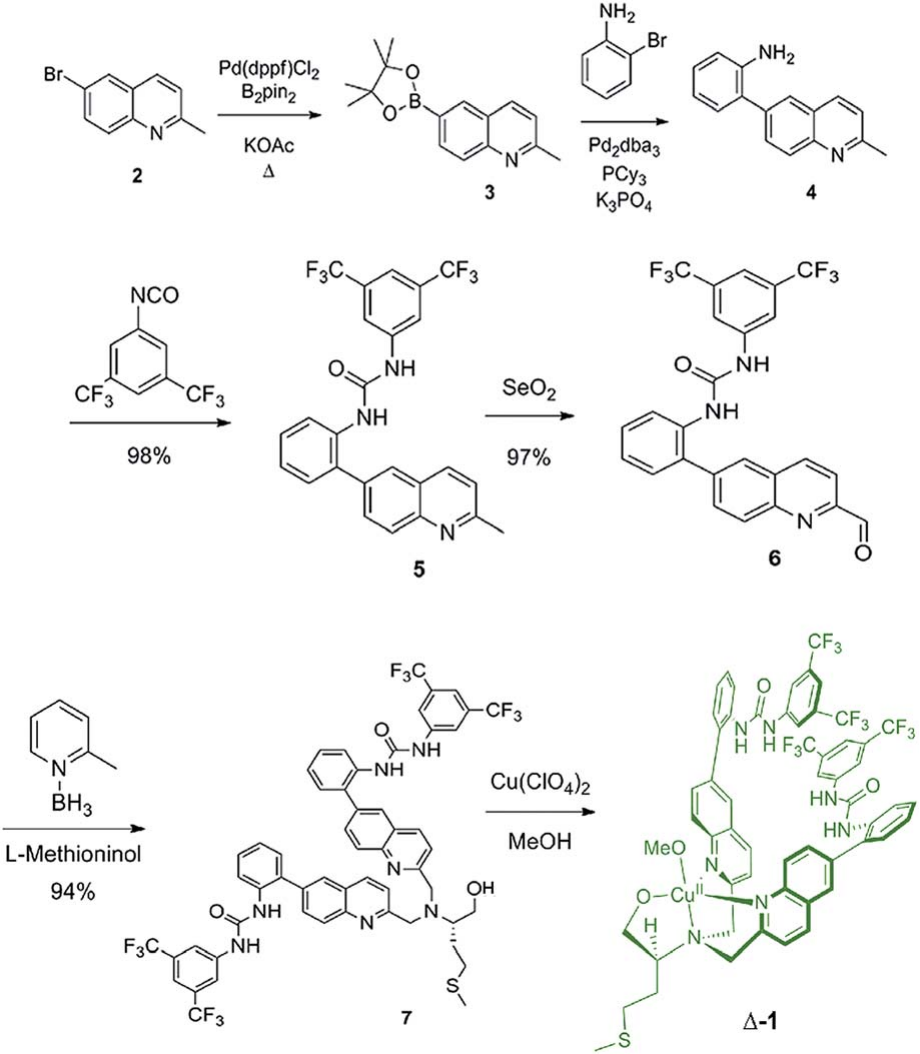
Synthetic route used to produce complex **Δ-1**.

The synthesis of ligand **7** began with the palladium-catalyzed Miyaura borylation of commercially available 6-bromo-2-methylquinoline (Scheme 1). A Suzuki coupling reaction was then performed with 2-bromoaniline using conditions that are amenable to the presence of heterocycles and amines to produce amine **4** in 68% overall yield for two steps.^33^ Amine **4** was then reacted with 3,5-bis(trifluoromethyl)phenyl isocyanate to produce urea **5** in high yield. The methyl group of urea **5** was then oxidized to an aldehyde using SeO_2_ in 97% yield. The reductive amination of aldehyde **6** with l-methioninol was performed using 2-picoline borane as the reducing agent to give di-alkylated ligand **7** in 94% yield.^34^ Four equivalents of aldehyde **6** were necessary to di-alkylate the amine fully due to unwanted reduction of the aldehyde to the corresponding alcohol. The alcohol byproduct can be isolated and re-oxidized with SeO_2_ to regenerate aldehyde **6**. The five-step sequence to ligand **7** proceeded in 61% yield overall. The copper complex (**Δ-1**) was then obtained after addition of copper(ii) perchlorate and filtration.

### Switching and readout

**Fig. 3:**
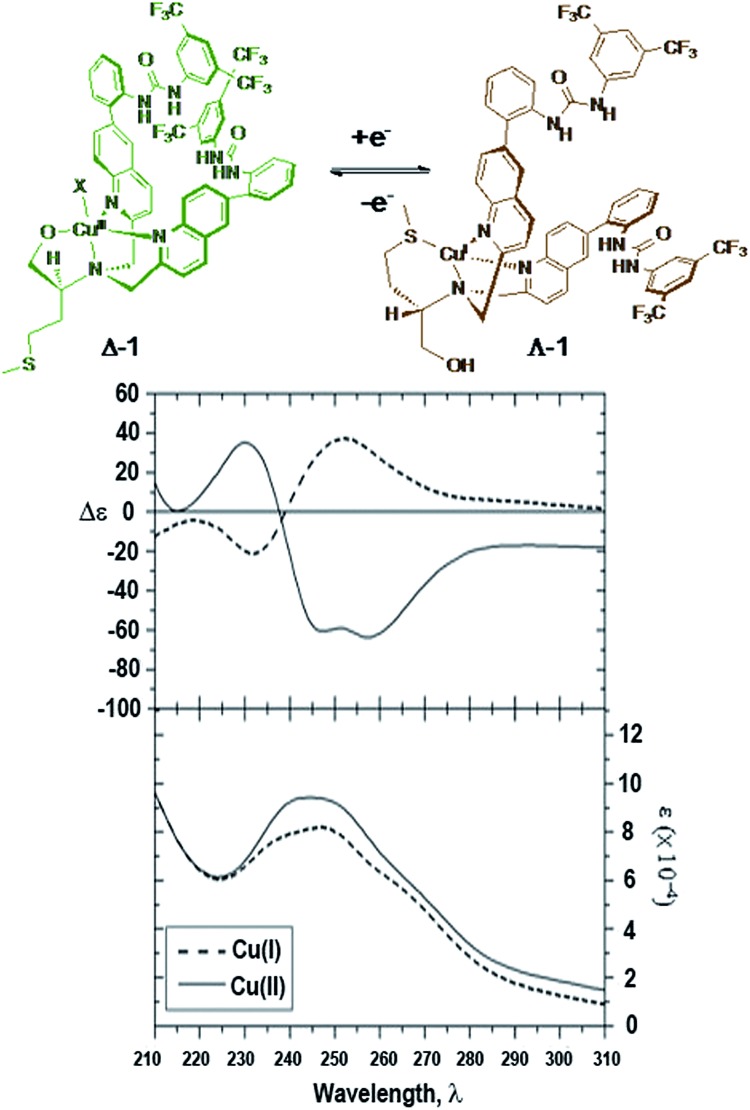
Redox triggered switching between **Δ-1** and **Λ-1**. CD (L mol^–1^ cm^–1^) and UV (L mol^–1^ cm^–1^) of **Δ**/**Λ-1** (59 μM, acetonitrile).

Before testing its catalytic properties, the ability to reconfigure the complex was tested. As with previously reported chiral molecular switches, UV and CD studies are crucial in determining whether or not the complex is capable of inverting helicity upon chemical oxidation or reduction of the copper center.^35^ Spectra were obtained in acetonitrile and methanol due to the low level of absorption of these solvents in the UV region of interest (Fig. 3).

Electronic spectra of both **Δ-1** and the Cu(ii) complex in Fig. 1 16 appear to be similar qualitatively. However, complex **Δ-1** displays additional features that likely arise from the presence of additional aromatic substituents that may absorb in the UV wavelength region. The absorption spectrum of **Δ-1** [Cu(ii)] showed a flattened peak suggesting transitions of similar intensity near 240 and 250 nm. The transition near 240 nm is likely due to the ^1^B_b_ transition with the dipole oriented in the longitudinal direction. This transition gives rise to an exciton couplet in the CD spectrum with a trough at 247 nm, null near 238 nm, and a peak near 230 nm. The trough at 258 nm is likely due to a π–π* transition involving the quinoline and attached phenyl ring. The **Λ-1** (Cu(I)) compound shows a broad peak near 248 nm in the absorbance spectrum, and associated peak, null, and trough at 252, 239, and 232 nm, respectively in the CD spectrum.^36^

Overall, the CD spectra of the **Δ-1** and **Λ-1** complexes exhibited significant mirror image ECCD character, consistent with inversion of the asymmetric orientation of the chromophores upon change in oxidation state. Chemical reduction of **Δ-1** was performed using l-ascorbic acid to attain **Λ-1**. However, **Λ-1** may also be obtained by addition of Cu(CH_3_CN)_4_PF_6_ to ligand **7**. CD/UV spectra of the copper(i) complex obtained by direct formation using a copper(i) precursor are identical to spectra of the copper(i) complex obtained by reduction of the copper(ii) complex. CD studies of **Δ-1** suggest that ligand reorganization takes place with inversion of helicity as hypothesized.

### Initial catalytic performance

**Scheme 2:**
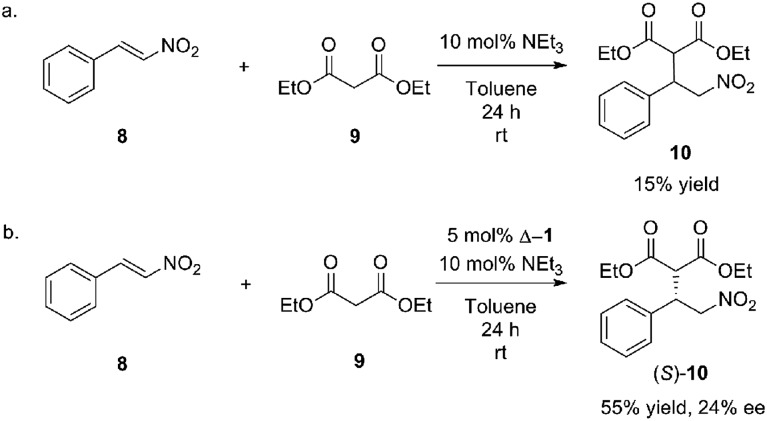
Preliminary catalyst results.

After UV/CD studies of **Δ-1** confirmed operational and controllable helicity inversion, the catalytic activity of **Δ-1** was tested. A model reaction inspired by Takemoto's work was identified to test the hypothesis that the catalyst could be reconfigured to give opposite stereoselectivity. Initial model studies were performed in toluene due to the higher affinity observed between urea and anionic substrates in nonpolar solvents (Scheme 2).

Triethylamine was used as a co-catalyst with 10 mol% loading with 5 mol% loading of **Δ-1**. The product nitroalkane (**10**) was obtained in 55% yield as opposed to 13% yield without **Δ-1**. The Michael adduct (*S*)-**10** was obtained with 24% ee for the (*S*)-product, affirming that the catalyst was imparting enantioselectivity to the reaction, albeit with low efficiency. These results compelled us to begin screening solvents with **Δ-1** and **Λ-1** to determine if reversal of enantioselectivity could be achieved under other conditions.

### Solvent effects

The ability of the catalyst to function ambidextrously in other solvents was tested by screening **Δ-1** and **Λ-1** for enantioselectivity in a variety of solvents. Additionally, the solvent screen was performed to test the effects of different solvents on the enantiomeric excess and yield of the reaction.

Reversal of enantioselectivity occurred in all solvents in which the catalyst is able to achieve at least a moderate enantiomeric excess. Acetonitrile provided the best combination of yield and enantioselectivity (Table 1).

**Table 1:** Effects of solvent on **Δ**/**Λ-1**^*a*^

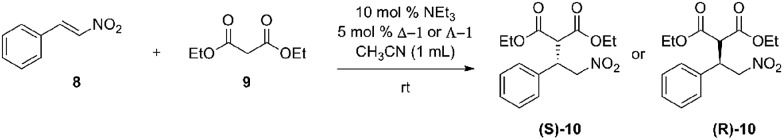				
Solvent	**Δ-1**		**Λ-1**	
	% ee of (*S*)-**10**^*b*^	% yield^*c*^	% ee of (*R*)-**10**	% yield
Toluene	24	55	51	33
THF	48	33	57	78
MeCN	72	55	70	40
CHCl_3_	30	40	68	34
CH_2_Cl_2_	46	44	74	43
Hexane	51	30	60	30
EtOAc	0	81	7	73
DCE	22	46	44	52
Dioxane	3	87	0	78
MeOH	20	35	5	35

^*a*^All reactions were performed using β-nitrostyrene **8** (0.34 mmol, 1 equiv.), diethylmalonate **9** (0.68 mmol, 2 equiv.), and NEt_3_ (0.034 mmol, 0.1 equiv.) in solvent (1 mL) with 5 mol% catalyst (**Δ-1** or **Λ-1**) at room temperature for 24 h.

^*b*^Determined by chiral HPLC analysis.

^*c*^Isolated yields.

The enantioselectivity is likely dependent on both the solvent's ability to stabilize binding of nitrostyrene to urea and allow an asymmetrical conformation of the catalyst in both oxidation states. As expected, reactions performed in nonpolar solvents outperformed polar solvents in stereoselectivity. This difference is presumably due to decreased substrate-urea binding in polar solvents. Yields were generally similar in all solvents tested, with the exception of dioxane and ethyl acetate. These solvents likely decrease the rate of competitive anionic polymerization of nitrostyrene catalyzed by the exogenous base.

### Base effects

Subsequent screens were performed testing the role of the base in the catalyzed reaction. Multiple bases were tested in both oxidation states (Table 2). With all bases tested, reversal of enantioselectivity was achieved. Stronger bases, such as DBU, gave both low yield and low stereoselectivity. This finding is expected due to the base's catalysis of the anionic polymerization of nitrostyrene (**8**).^37^ Low yields were also obtained with DMAP. Insoluble polymer side products formed almost immediately after addition of these bases.

**Table 2:** Base screen for different complex oxidation states^*a*^

Base	**Δ-1**		**Λ-1**	
	% ee^*b*^	% yield of (*S*)-**10**^*c*^	% ee	% yield of (*R*)-**10**
NEt_3_	72	55	70	40
DIPEA	70	38	56	32
DABCO	66	44	30	48
DBU	34	40	7	34
DMAP	57	21	31	32

^*a*^All reactions were performed using β-nitrostyrene **8** (0.34 mmol, 1 equiv.), diethylmalonate **9** (0.68 mmol, 2 equiv.), and base (0.034 mmol, 0.1 equiv.) in MeCN (1 mL) with 5 mol% catalyst (**Δ-1** or **Λ-1**) at room temperature for 24 h.

^*b*^Determined by chiral HPLC analysis.

^*c*^Isolated yields.

Hindered bases with moderate conjugate acid p*K*_a_ values, such as DIPEA and NEt_3_, performed the best from this screen.^38^ These results led us to do a more comprehensive base screen performed in only one of the catalyst states varying steric effects and basicity (Table 3). Indeed, the best results were obtained using bases that had high steric hindrance and a moderately high basicity as indicated by the p*K*_a_ of the conjugate acid in acetonitrile.

**Table 3:** Base screen for basicity and steric effects^*a*^

Base	**Δ-1**		
	% ee^*b*^	% yield of (*S*)-**10**^*c*^	p*K*_a_^*d*^
DBU	34	20	24.33
NEt_3_	72	55	18.82
DIEA	70	38	18.80
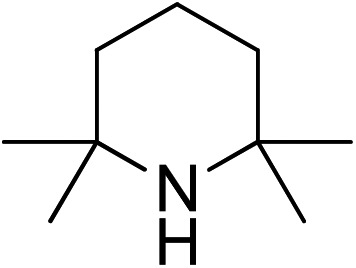	53	33	18.64
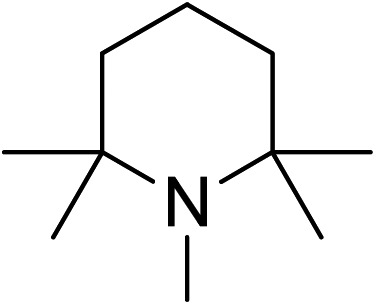	66	43	18.62
DABCO	66	44	18.29
DMAP	57	21	17.95
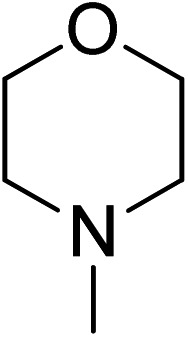	40	17	15.8
Collidine	10	8	14.77
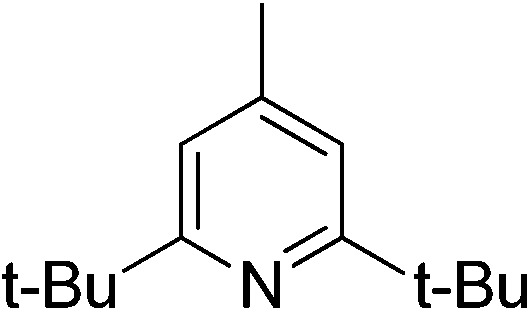	0	21	13–14
TBAF	20	61	?

^*a*^All reactions were performed using β-nitrostyrene **8** (0.34 mmol, 1 equiv.), diethylmalonate **9** (0.68 mmol, 2 equiv.), and base (0.034 mmol, 0.1 equiv.) in MeCN (1 mL) with 5 mol% catalyst (**Δ-1**) at room temperature for 24 h.

^*b*^Determined by chiral HPLC analysis.

^*c*^Isolated yields.

^*d*^p*K*_a_ of conjugate acid in acetonitrile.^38^

Sterically hindered bases, such as collidine and 2,6-di-*tert*-butyl-4-methylpyridine, provide low yields and stereoselectivities whereas bases that are stronger and less sterically hindered, like DMAP and DABCO, afford higher ee values but still suffer from low yields. TBAF was tested as a base in hopes that the low nucleophilicity would decrease the polymerization side reaction, but low stereoselectivity was afforded.

### Substrate scope

We examined the catalyst against a series of different malonate and nitrostyrene substrates (Table 4). The Michael addition reaction with diethyl malonate proceeded with various substituents bound to the nitrostyrene. The yields seemed to be dependent on the stability of the nitrostyrene to base-catalyzed anionic polymerization as can be seen from the 97–98% yield obtained for the dimethoxy substrate (entry 3, Table 4). The stereoselectivities for the substituted nitrostyrenes were lower in some cases, but a clear pattern has yet to emerge. Different malonates were also tested. The more sterically hindered diisopropyl malonate (entry 7, Table 4) gave lower yields than diethyl malonate. In nearly all cases, the enantioselectivity was reversed with a change in copper oxidation state, with the exception being acetylacetone (entry 8, Table 4). The use of acac reduced the **Δ-1** to **Λ-1** yielding (*R*)-**10h** in 60–70% ee. Control studies performed without nitrostyrene show that in the presence of NEt_3_ and acac in acetonitrile, **Δ-1** is reduced to a complex with a circular dichroism spectrum identical to that of **Λ-1**. This behaviour was seen with other ketone containing 1,3-dicarbonyl substrates as well. It is unclear exactly which side reaction results in the reduction of the Cu(ii), but literature suggests a copper-catalyzed homocoupling of dicarbonyl compound is possible.^39^

**Table 4:** Enantioselective reaction of **Δ**/**Λ-1** with different substrates^*a*^

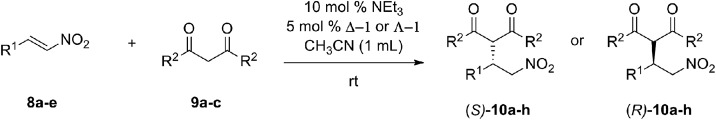						
Entry	**Δ-1**			**Λ-1**		
	R^1^	R^2^	% ee^*b*^	% yield of (*S*)-adduct^*c*^	% ee	% yield of (*R*)-adduct
1 (**10a**)	4-MeC_6_H_4_	OEt	70	67	67	55
2 (**10b**)	4-OMeC_6_H_4_	OEt	60	46	73	20
3 (**10c**)	2,3-(OMe)_2_C_6_H_3_	OEt	48	97	57	98
4 (**10d**)	4-BrC_6_H_4_	OEt	24	45	72	48
5 (**10e**)	4-OHC_6_H_4_	OEt	40	51	53	44
6 (**10f**)	Ph	OMe	57	35	64	77
7 (**10g**)	Ph	i-PrO	73	30	65	30
8 (**10h**)	Ph	Me	60 (*R*)	80 (*R*)	70	78

^*a*^All reactions were performed using nitrostyrene (0.34 mmol, 1 equiv.), malonate (0.68 mmol, 2 equiv.), and NEt_3_ (0.034 mmol, 0.1 equiv.) in MeCN (1 mL) with 5 mol% catalyst (**Δ-1** or **Λ-1**) at room temperature for 3–24 h.

^*b*^Determined by chiral HPLC analysis.

^*c*^Isolated yields.

In the studies shown previously, the malonate compounds lacked a prochiral center so that the products formed were enantiomers. Reaction of prochiral malonates with nitrostyrene using the ambidextrous catalyst could produce both diastereomeric and enantiomeric products. Four prochiral malonates were tested (Table 5) with nitrostyrene **8** using **Δ-1** and **Λ-1**. Moderate yields (39–54%) were obtained. Again, enantioselectivity was reversed upon a change in oxidation state of the copper center. In these experiments, the magnitude of enantioselective inversion (Δee_i_) is the sum of the two ee's obtained for the two diastereomers produced using the catalyst in its two different oxidation states. The magnitudes of Δee_i_ were inconsistent, as the product **10j** (entry 2, Table 5) was obtained with similar enantiomeric excess in both catalyst oxidation states (Δee_i_ = 46 for *d*_1_, 50 for *d*_2_) while product **10k** (entry 3, Table 5) was obtained with higher enantioselectivity (77/72%) in the **Δ-1** state than in the **Λ-1** state (20/24%). In the case of **10k**, if the enantioselectivity were the same in the two catalyst oxidation states, the Δee_i_ for each pair of diastereomers would be ∼140–150. The experimental Δee's (97% for *d*_1_, 96% for *d*_2_) are well below the values expected from enantiomeric transition states for unknown reasons. It should be noted that no inversion of diastereoselectivity was obtained, yet inversion of enantioselectivity was achieved for these compounds.

**Table 5:** Stereoselective reaction of **Δ**/**Λ-1** with prochiral malonates^*a*^

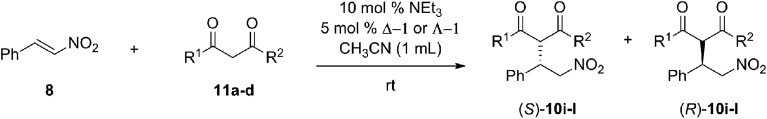						
Entry	**Δ-1**				**Λ-1**	
	R^1^	R^2^	% ee^*b*^ *d*_1_/*d*_2_	% yield of (*S*)-adduct^*c*^	% ee *d*_1_/*d*_2_	% yield of (*R*)-adduct
1 (**10i**)	OMe	Ot-Bu	60/73	54 (dr ∼ 70 : 30)^*d*^	64/64	40 (dr ∼ 68 : 32)
2 (**10j**)	OBz	OEt	26/30	41 (dr ∼ 50 : 50)	20/20	40 (dr ∼ 50 : 50)
3 (**10k**)	OEt	Ot-Bu	77/72	46 (dr ∼ 50 : 50)	20/24	39 (dr ∼ 50 : 50)
4 (**10l**)	OBz	Ot-Bu	37/70	51 (dr ∼ 67 : 32)	50/40	42 (dr ∼ 50 : 50)

^*a*^All reactions were carried out using β-nitrostyrene (0.34 mmol, 1 equiv. malonate (0.68 mmol, 2 equiv.)), and NEt_3_ (0.034 mmol, 0.1 equiv.) in MeCN (1 mL) with 5 mol% catalyst (**Δ-1** or **Λ-1**) at room temperature for 3–24 h.

^*b*^Determined by chiral HPLC analysis (*d*_1_ = diastereomer 1).

^*c*^Isolated yield.

^*d*^(dr = *d*_1_ : *d*_2_).

### Effects of copper salt

Various copper salts were premixed *in situ* with ligand **7** to determine if isolation of the complex prior to use as a catalyst was necessary and to test the effects of different counter ions on the Michael addition reaction (Table 6).

**Table 6:** Effects of different copper salts on catalysis^*a*^

Entry	Cu^2+^ salt	% ee^*b*^	% yield of (*S*)-**10**^*c*^
1	Cu(ClO_4_)_2_·6H_2_O	67	64
2^*d*^	Cu(ClO_4_)_2_·6H_2_O	72	63
3^*d*^	CuCl_2_·2H_2_O	20	55
4^*d*^	Cu(BF_4_)_2_	70	50
5^*d*^	Cu(NO_3_)_2_·H_2_O	37	57

^*a*^All reactions were carried out using β-nitrostyrene **8** (0.34 mmol, 1 equiv.), diethyl malonate **9** (0.68 mmol, 2 equiv.), and NEt_3_ (0.034 mmol, 0.1 equiv.) in MeCN (1 mL) with 5 mol% catalyst (formed *in situ*) at room temperature for 3–24 h.

^*b*^Determined by chiral HPLC analysis.

^*c*^Isolated yields.

^*d*^4 Å MS added.

The effect of metal source on the Cu(ii) catalyst was tested since spatial modulation of the complex could occur if different ions were to bind to the copper atom at its open coordination site.^36^ The absence of coordinative unsaturation in the Cu(i) catalyst discouraged us from screening the synthesis of this catalyst with various counter ions. The isolation of every copper complex was undesirable from a practical standpoint, so we first tested whether the catalyst complex could be formed *in situ* before addition of the reagents. Experiments using Cu(ii) perchlorate hexahydrate (entry 1, Table 6) as the copper source showed that the reaction occurred with slightly lower ee (67%) and higher yield (64%) than when the complex was isolated prior to being employed as a catalyst. The lower enantioselectivity was attributed to the presence of water molecules that originate from the hygroscopic copper perchlorate. The addition of 4 Å molecular sieves remedied this issue (entry 2, Table 6). Other copper salts, such as CuCl_2_ and Cu(NO_3_)_2_, have a noticeable impact on the reaction, providing product (*S*)-**10** with lower enantioselectivity. This result can be attributed either to urea-anion binding or copper-anion coordination. Urea-containing anion sensors have proven capable of binding anions such as Cl^–^ and NO_3_^–^ which could limit catalyst efficiency by blocking nitrostyrene binding. Chloride is also known to coordinate with high affinity to Cu(ii), which could cause a disturbance in catalyst configuration that lowers the ee. Anions that bind weakly to copper and urea, such as BF_4_^–^ and ClO_4_^–^, yielded higher stereoselectivities (entries 2 and 4, Table 6).

### Kinetics

**Fig. 4:**
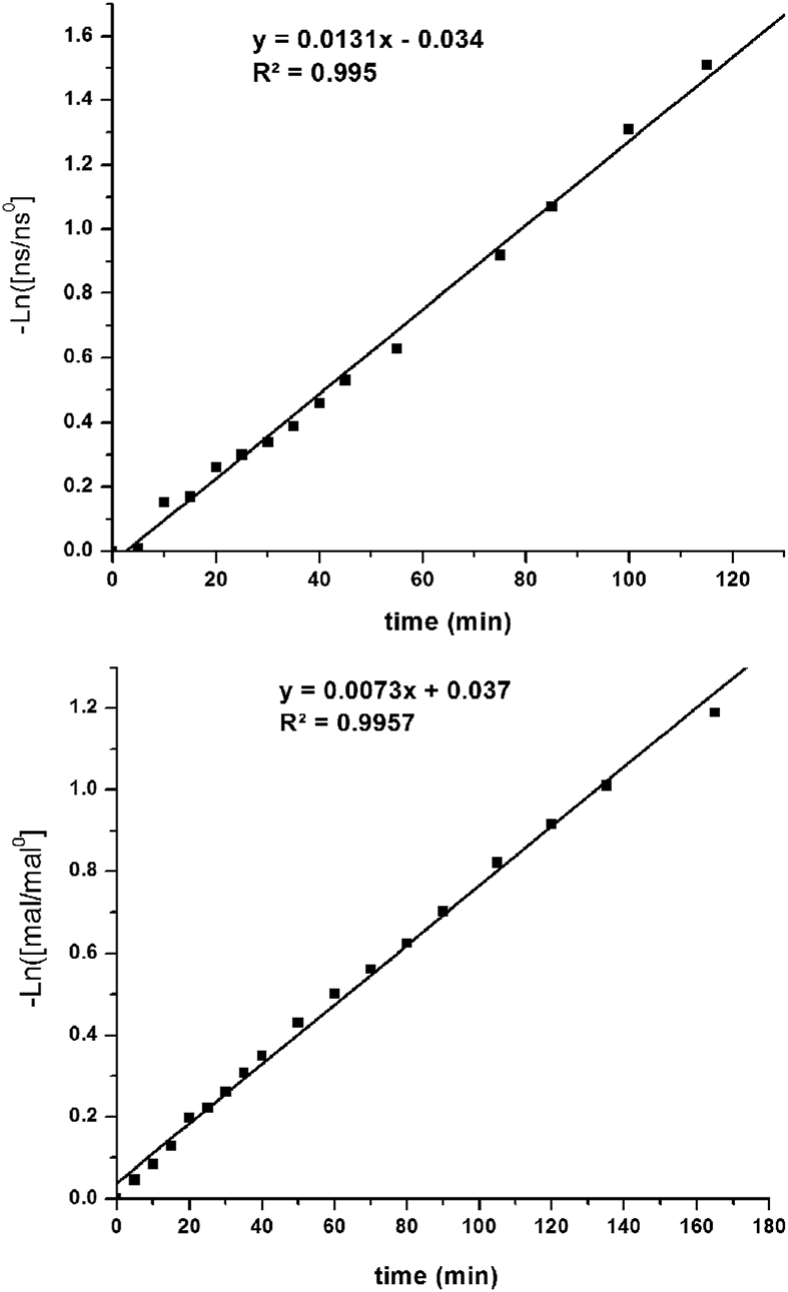
Pseudo first order rate studies performed in the presence of excess diethyl malonate (top) and excess nitrostyrene (bottom) (mal = **9**, ns = **8c**).

The kinetic parameters of the reaction were examined using pseudo-first-order rate studies.^40^ Nitrostyrene **8c** was used due to its resistance to base catalyzed degradation. When the reaction using **Δ-1** was carried out in the presence of excess diethyl malonate, the plot of ln([mal/mal^0^]) *versus* time exhibited a linear correlation, which indicates the reaction is first-order with respect to nitrostyrene (Fig. 4, top). A rate of 2.2 × 10^–4^ M s^–1^ was observed. The same studies were performed in excess nitrostyrene and indicated the reaction was also first-order with respect to diethyl malonate (Fig. 4, bottom). A similar rate of 1.2 × 10^–4^ M s^–1^ was observed.

**Fig. 5:**
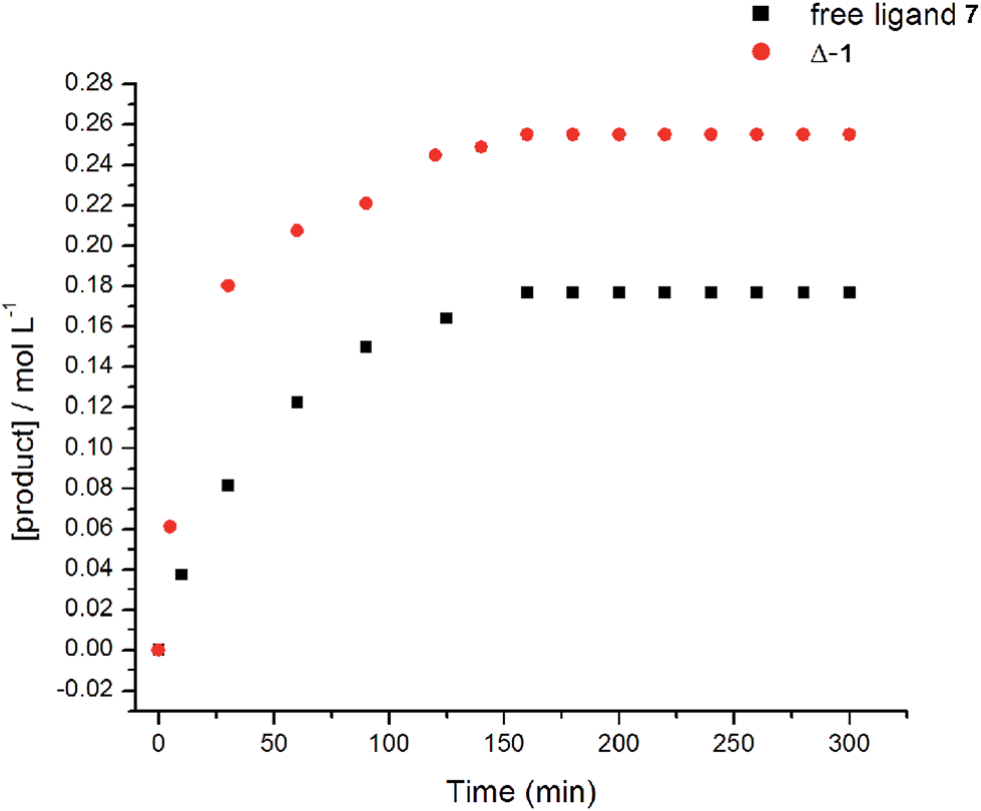
Reaction kinetics monitoring formation of product (*S*)-**10** in the presence of free ligand **7** and **Δ-1**.

We were also interested in whether one or both urea groups were active in the transition state of the reaction. The rate of the reaction using free ligand as catalyst was compared with the rate using **Δ-1** (Fig. 5). The free ligand is much less rigid and the low energy conformations tend to place the urea groups much farther apart than in the metal complexes. Therefore, if both urea groups are active in the transition state, then use of the flexible free ligand may result in a slower rate of reaction. However, the energetic cost for the urea moieties to achieve the right orientation for reaction would not be high, so the rate may not be greatly affected. The yields and reaction rates observed using free ligand [*k*_obs_ = (1.9 ± 0.1) × 10^–3^ M^–1^ s^–1^] and **Δ-1** [*k*_obs_ = (5.0 ± 0.1) × 10^–4^ M^–1^ s^–1^] were of the same order of magnitude with both compounds. This may suggest that only one urea group is likely active in the transition state of the Michael addition. Indeed, literature examples of bifunctional urea catalysts do not always show large rate enhancement for covalent linkage of two reactive moieties.^28^ This data instigated further examination into the mechanism of the catalyst.

### Crystal structure and stereochemical rationale

Initial modeling studies were performed by building a space filling model by modification of previously obtained X-ray data from a related complex. The model suggested that the urea groups position themselves nearly perpendicular to one another, creating a chiral cleft. The two oxidation states of the catalyst produce mirror image chiral substrate binding clefts that lead to enantiomeric transition states. The previous kinetics data studies show first order reaction in diethyl malonate, nitrostyrene, and base. This would be consistent with all of the substrates binding concurrently in the chiral cleft, or with deprotonation of the diethyl malonate followed by attack of the cleft-bound nitrostyrene. The model suggests that nitrostyrene is comfortable bound in the cleft, with little extra room for concomitant interactions with the diethyl malonate. To examine the structure of the catalyst more closely, a crystal suitable for X-ray diffraction was obtained by slow diffusion of water into a concentrated solution of **Δ-1** in acetonitrile.

**Fig. 6:**
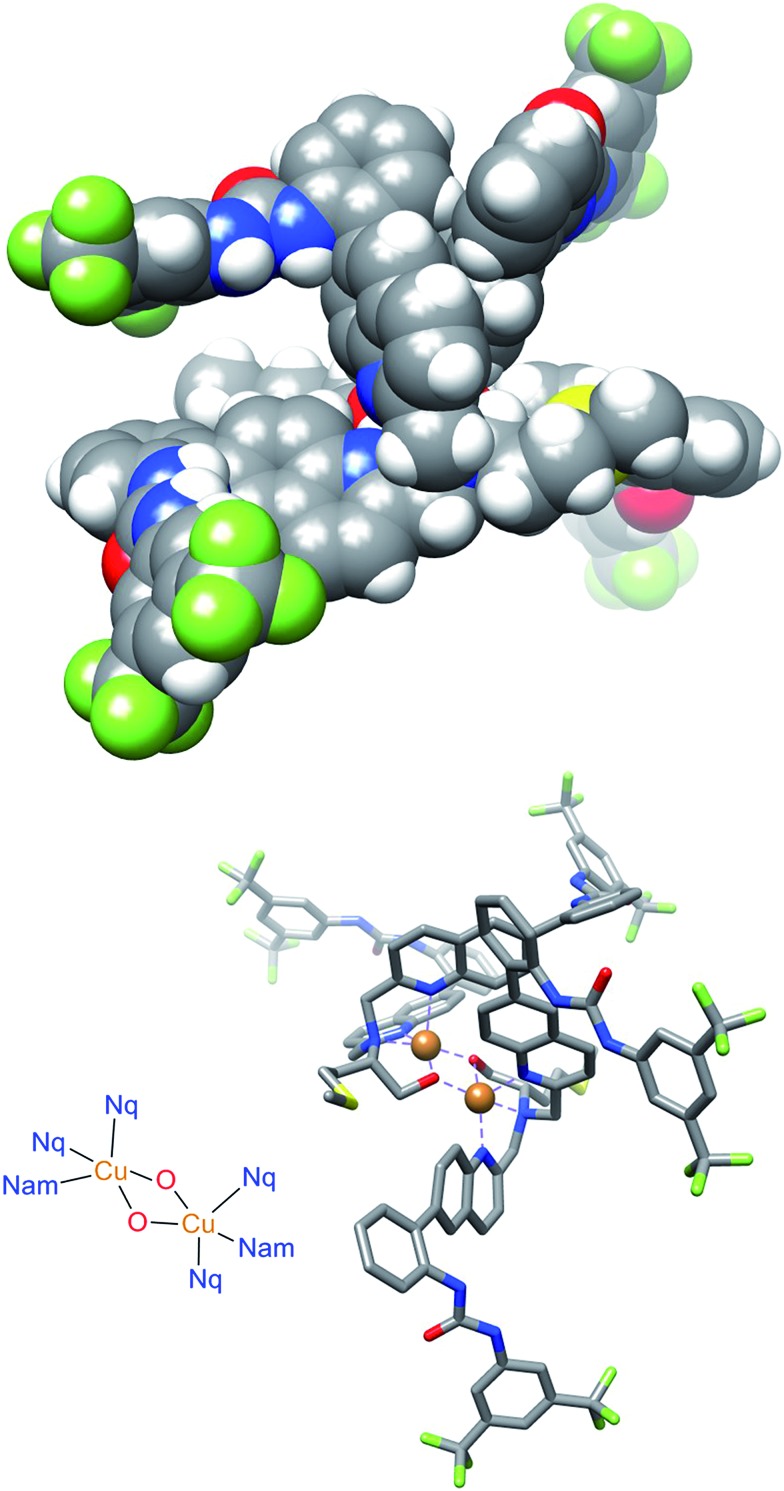
The space filling (top) and stick (bottom) molecular structure of **Δ-1**. The disordered groups, perchlorate ions, acetonitrile molecules, and hydrogen atoms are omitted for clarity.

The crystal structure of **Δ-1** exists as a dimer with a bis(μ-alkoxo)dicopper(ii) bridge connecting the monomers (Fig. 6). Two quinoline nitrogens and oxygen from deprotonated hydroxyl group form the trigonal plane with the central copper ion. A second oxygen atom from the adjacent dimer and tertiary nitrogen occupy apical positions. Previous crystallographic structures of similar Cu(ii) complexes suggest that complexes with coordinated alcohol are monomeric while the deprotonated alkoxo ligands form dimers.^41^ It is unclear as to whether the active catalyst is the monomer or the dimer, and this is the subject of ongoing study. This issue does not appear to be of great significance regarding the catalytic mechanism, as the space filling model (Fig. 6, top) contains a chiral cleft that is similar to that hypothesized earlier in the modeling study (Fig. 2).

In the solid state, the dimers are organized into supramolecular polymers *via* intermolecular hydrogen bonding between urea groups. It is well known that urea catalysts usually suffer from decreased activity due to self-quenching through dimerization or oligomerization.^42^ However, in this catalyst solid state structure, some urea groups remain free for maximum activity. Two hydrogen bonds are present between urea groups of the adjacent dimers, but two are not hydrogen bonded. Noticeably, a chiral cleft was also formed, providing a chiral environment allowing asymmetric reaction.

Unfortunately, binding studies with **Λ-1** in the presence of both nitrostyrene and diethyl malonate using NMR spectroscopy were unsuccessful. Although the urea N–H protons of the free ligand could be accurately assigned, these N–H protons upon the addition of nitrostyrene or diethylmalonate gave inconsistent peak shifts. Addition of NEt_3_ also caused peak shifts that led to inconclusive results. Additionally, UV and CD studies were conducted to test for shifts in the absorption peaks upon addition of substrates, but no shifts occurred in either spectrum with the addition of either substrate.

**Fig. 7:**
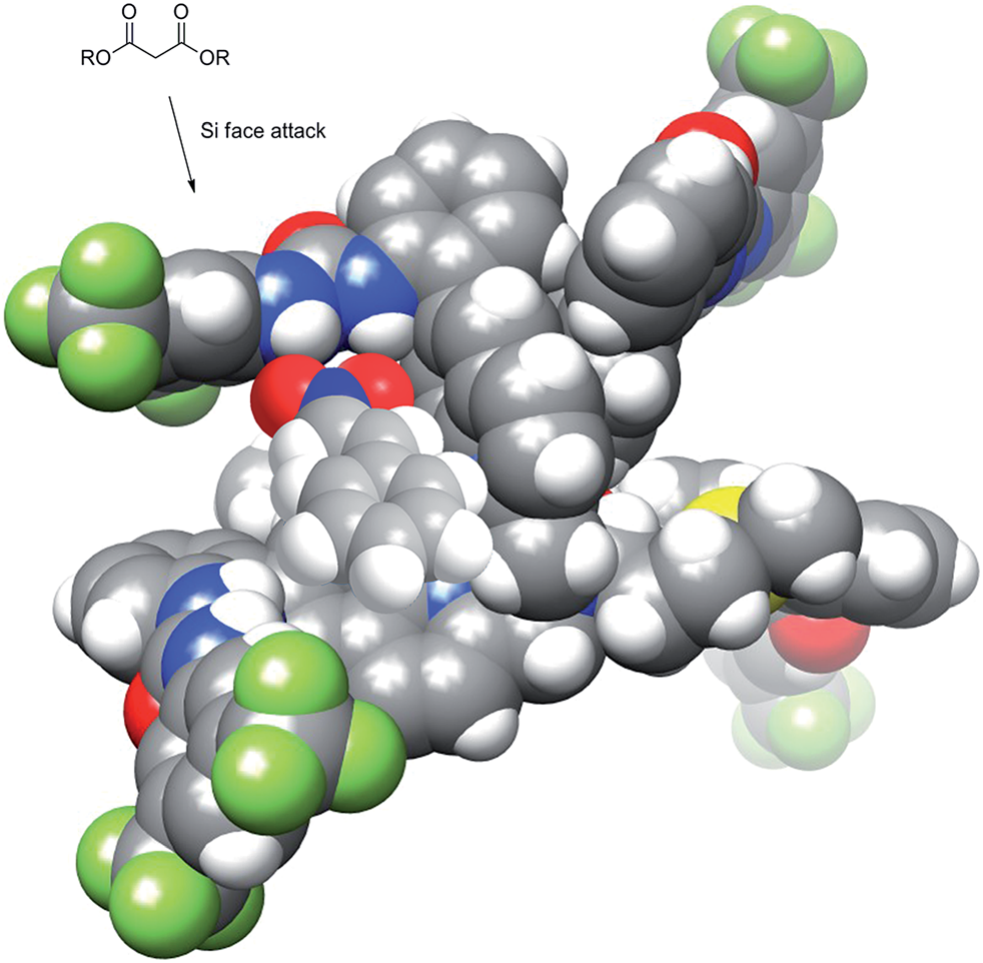
Model of catalyst showing hindered attack at *Re* face of hydrogen bonded nitrostyrene.

Previously designed (thio)urea catalysts that perform the same or similar reactions are thought to bind and activate nitrostyrene by hydrogen bonding of the urea N–H to the two oxygen atoms of the nitro group. This same mechanistic rationale can be applied to **Δ-1**/**Λ-1** with the nitrostyrene binding in the chiral cleft created by the urea groups. The chirality of the cleft then becomes the major determining factor of asymmetric induction. Fig. 7 shows the nitrostyrene bound to the catalyst in a geometry where the approach of diethyl malonate from the *Re* face of the nitrostyrene is blocked by the catalyst. This allows for approach exclusively at the *Si* face, which is less sterically encumbered by the scaffold of the catalyst.

This mechanism takes into account that only one urea is involved in activation of the nitrostyrene substrate and the first-order dependence on both nitrostyrene and diethyl malonate. After C–C bond formation, the resulting nitroalkane anion is protonated by the conjugate acid of NEt_3_, regenerating the catalytic NEt_3_.

The enantioselectivity of the reaction may be limited by the conformational bias in the molecule that accounts for the helical dissymmetry of the two urea-containing arms. The difference in energies of the pseudoenantiomeric conformations of a closely related complex was estimated to be 1.5–2.5 kcal mol^–1^.^43^ The ambidextrous catalyst should have a similar difference in energies for the pseudoenantiomeric states. A Δ*G* value of 1.1 kcal mol^–1^ was calculated from the enantiomeric excess obtained using **Δ-1** at room temperature. The lower free energy value obtained from the enantiomeric excess may be associated with the lower rigidity found in the urea portion of the catalyst.

An appealing aspect of the ambidextrous catalyst is the ease of handling. The catalyst was obtained as the copper complex by simple filtration as the Cu^2+^ or Cu^+^ salt and used afterwards. Additionally, the air stable Cu^2+^ complex (**Δ-1**) was stored and reduced using l-ascorbic acid to isolate **Λ-1**. Model reactions showed nearly identical yields and ee values resulted whether the copper(i) catalyst was synthesized using a Cu^+^ precursor or was formed by reduction of the copper(ii) catalyst.

**Fig. 8:**
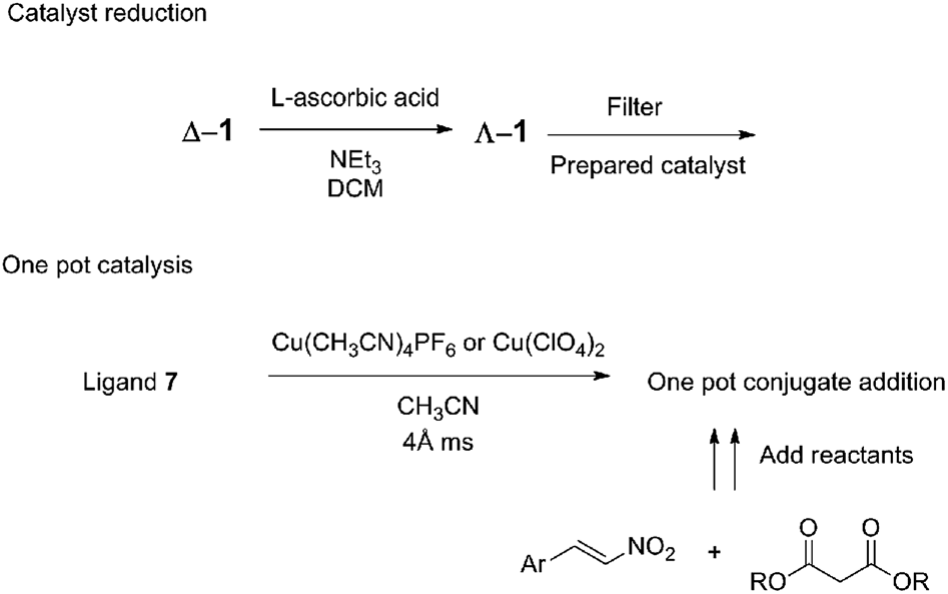
Practical handling the ambidextrous catalyst allows for both *in situ* catalyst reduction and one pot methods.

It has been shown that the complex itself does not have to be isolated because the complexation was completed *in situ* in acetonitrile with either Cu(ClO_4_)_2_ or Cu(CH_3_CN)_4_PF_6_ and the substrates added afterwards (Fig. 8). When the desired reaction was to be completed in a different solvent, complexation was performed in acetonitrile, and the acetonitrile was then removed *in vacuo*. The *in situ* formation of the copper(ii) complex was performed in the presence of 4 Å molecular sieves to absorb the water present in the hygroscopic Cu^2+^ salts.

## Conclusion

A redox reconfigurable copper complex was synthesized that was capable of catalyzing the formation of an asymmetric nitrostyrene conjugate addition. The stereoselectivity could be modulated to afford either *R* or *S* product depending on the oxidation state of the copper center. The catalyst was designed by appending urea groups to a reconfigurable scaffold that was capable of inverting helicity upon oxidation/reduction of the copper center. The urea groups were the only active species influencing catalysis while the copper ion was used to control the handedness of the scaffold's helical structure. The ability to select which enantiomer of product was formed persists with most solvents, copper salts, and bases tested. A broad scope of symmetrical malonates, prochiral malonates, and nitrostyrene substrates were amenable to the synthesis of both enantiomers of product. Kinetic studies, crystal structure analysis, and molecular modeling suggest a mechanism in which one urea group binds and activates a nitrostyrene molecule in a chiral cleft during the stereochemistry-determining step. Malonate attack likely occurs on just one face of the nitrostyrene, affording the conjugate addition product. The mirror image chiral cleft is formed upon catalyst reconfiguration leading to a nearly mirror image transition state. The handling and usability of the catalyst was versatile and allowed several options for isolation or *in situ* use.

## Supplementary Material

Supplementary information

Crystal structure data
