# Supplementary material for: Redox-configurable ambidextrous catalysis: structural and mechanistic insight
Source: Chem Sci. 2015 Jul 14;6(10):5904–12. doi: 10.1039/c5sc02144h (PMC5950827; doi:10.1039/c5sc02144h)
Supplement: Supplementary file 1 [file SC-006-C5SC02144H-s001.pdf]

Electronic Supplementary Material (ESI) for Chemical Science.
This journal is © The Royal Society of Chemistry 2015

Asymmetric Conjugate Addition of Malonates to Nitrostyrenes using Ambidextrous Catalysis

Shahab Mortezaei, Noelle R. Catarineu, Xueyou Duan, Chunhua Hu, and James W. Canary*

Department of Chemistry, New York University, New York, New York 10003, United States

Table of Contents……………………………………………………. ………………**SI-1**

I. General Information……………………………………………………………….**SI-2**

II. Synthesis and Characterization of Catalyst and Products……………………..**SI-3**

III. Method for *in situ* Catalysis……………………………………………………..**SI-96**

IV. Crystallographic information for Δ-1…………………………………………...**SI-97**

V. References……………………………………………………………………….**SI-99**

I. General Information

General experimental information

All commercial materials were purchased from commercial sources and used without further purification. Small scale reactions were carried out in 20 mL Fisher Scientific disposable scintillation vials. Other reactions were carried out using oven-dried (160 °C) glassware. Solvents were purchased from Aldrich with Sure-Seal bottles. ^{1}H-NMR spectra were obtained using a Bruker AC 400 (400 MHz) NMR. ^{13}C-NMR spectra were obtained at 100 MHz on the same spectrometer. All chemical shifts are reported in parts per million (ppm) with reference to TMS = 0 ppm. The ESI mass spectra were obtained with an Agilent 1100 Series Capillary LCMSD Trap XCT Spectrometer using acetonitrile solutions. Absorption spectra were recorded on a Perkin Elmer Lambda spectroscope. Circular dichroism spectra were acquired with an AVIV Model 202SF CD spectroscope. All $\Delta\varepsilon$ units are L mol^{-1} cm^{-1}. The ee was determined on an Agilent 1200 series HPLC with Daicel Chemical Industries, LTD chiral columns. Chiralpak OD (0.46 cm x 25 cm), Chiralpak AD-H (0.46 cm x 25 cm), Chiralpak OD-H (0.46 cm x 25 cm), and Chiralpak OJ-H (0.46 cm x 25 cm) columns were used.

II. Synthesis and Characterization of Catalyst and Products

(2-methylquinolin-6-yl)aniline (4):

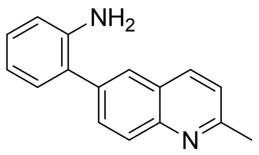

A 50 mL round bottom flask was charged with 1.0 g (4.5 mmol) of 6-bromo-2-methylquinoline (**2**), 1.25 g (5 mmol) bis(pinacolato)diboron, and 20 mL of toluene. The mixture was degassed using argon for 10 min. Pd(dppf)Cl_2 (330 mg, 0.45 mmol) and potassium acetate (1.3 g, 13.5 mmol) were then added and the reaction was heated at 90 °C under an argon atmosphere for 18 h. The reaction was allowed to cool to room temperature before filtering through a pad of silica. The solvent was then removed *in vacuo*. The crude product (1.19 g) was obtained and used without further purification for the Suzuki coupling. To 1.19 g of borylation product was added 605 μL (5.4 mmol) of 2-bromoaniline and a mixture of 30 mL dioxane/ 10 mL (1.3M) aq. K_3PO_4. The mixture was then purged using argon. Pd_2dba_3 (274 mg, 0.3 mmol) and PCy_3 (160 mg, 0.6 mmol) were then added to the reaction mixture before fitting the flask with a reflux condenser and heating the reaction to 100 °C under an inert atmosphere for 17 h. The reaction was allowed to cool, and the mixture was filtered through a pad of silica, which was washed with copious amounts of ethyl acetate. The solvent was removed *in vacuo* and crude was extracted with CH_2Cl_2 (3 x 50 mL). The organic layers were combined and washed with H_2O and brine and subsequently dried over Na_2SO_4 before being concentrated *in vacuo* and chromatographed

(silica gel, 9:1 CH_2Cl_2/EtOAc then 1:1 CH_2Cl_2/EtOAc) to give amine **4** as a solid (720 mg, 68%). Analytical data for title compound: ^{1}H NMR (400 MHz, $CDCl_3$): δ 8.07 (td, J = 8.1, 7.7, 0.8 Hz, 2H), 7.85 (d, J = 2.0 Hz, 1H), 7.80 (dd, J = 8.6, 2.0 Hz, 1H), 7.32 (d, J = 8.3 Hz, 1H), 7.24 – 7.16 (m, 2H), 6.87 (td, J = 7.5, 1.2 Hz, 1H), 6.83 – 6.78 (m, 1H), 3.80 (s, 2H), 2.77 (s, 3H); ^{13}C NMR (100 MHz, $CDCl_3$): δ 159.2, 147.1, 143.8, 137.0, 136.2, 131.0, 130.7, 129.1, 128.9, 127.5, 126.9, 126.7, 122.5, 118.8, 115.9, 25.5, 25.0 (bpin impurity); HRMS (EI) calc. for $[C_{16}H_{14}N_2]^+$ 234 [illegible] 22, 1158, 835, 752 c[illegible]

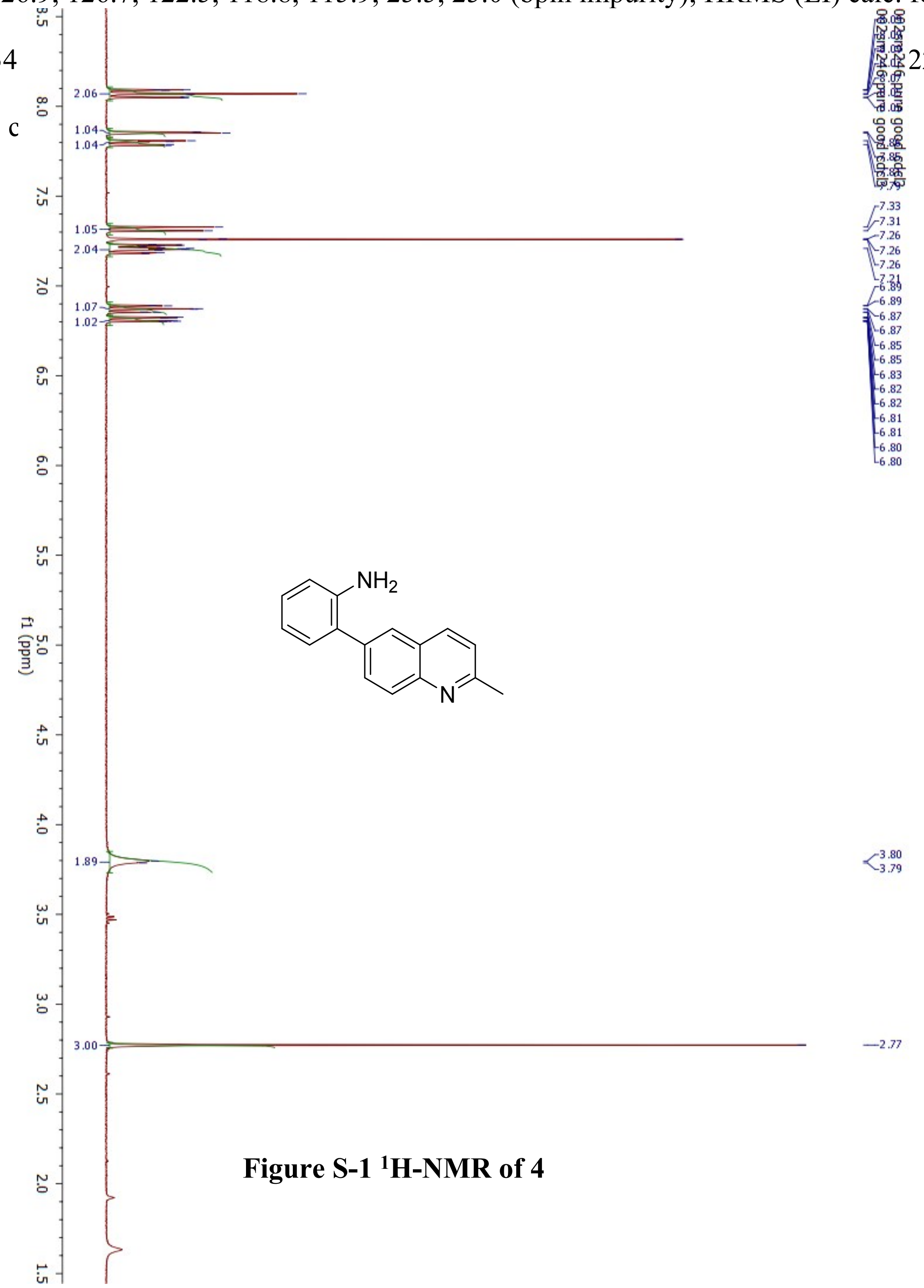

Figure S-1 ^{1}H-NMR of 4

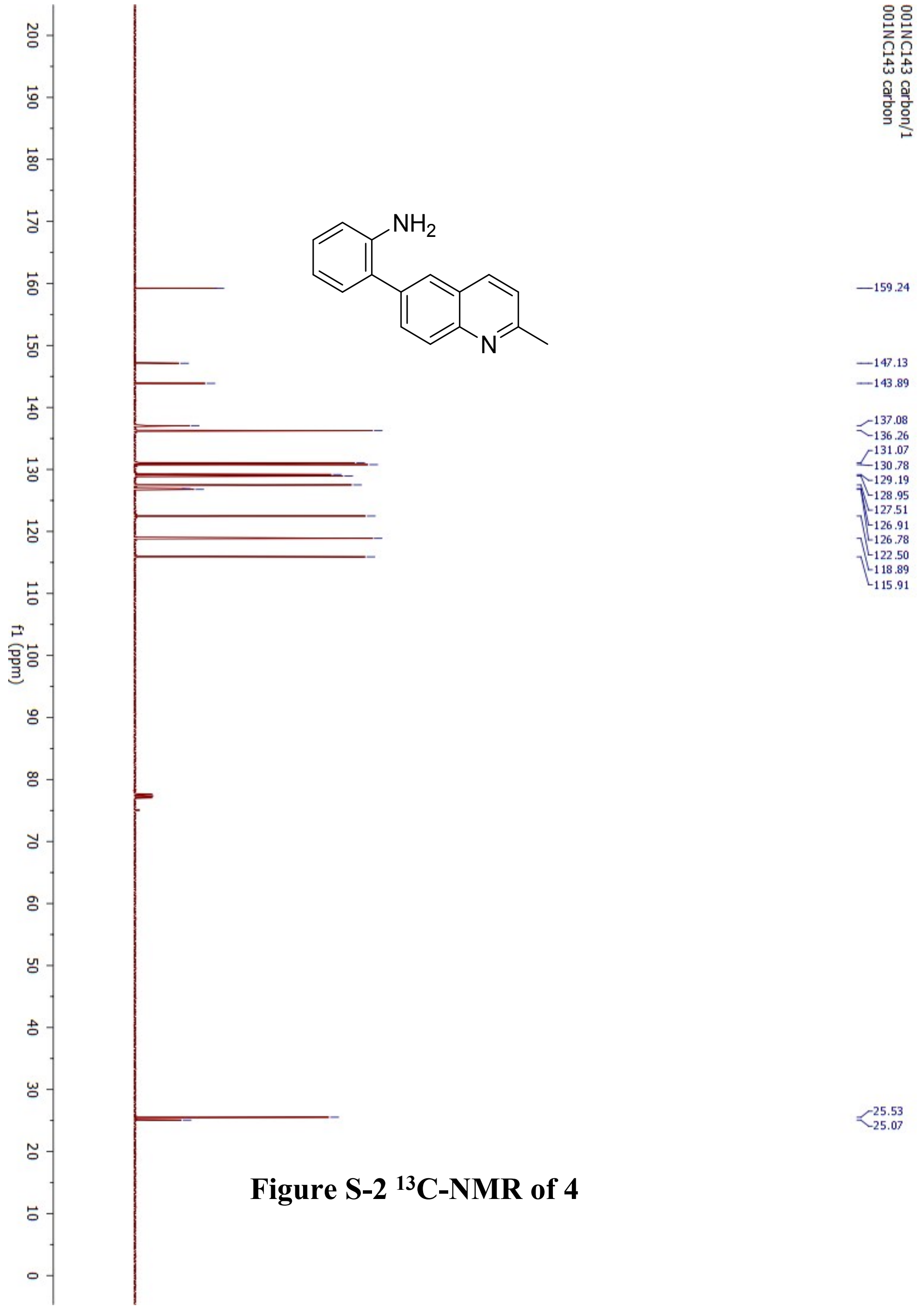

Figure S-2 ^{13}C-NMR of 4

1-(3,5-bis(trifluoromethyl)phenyl)-3-(2-(2-methylquinolin-6-yl)phenyl)urea (5):

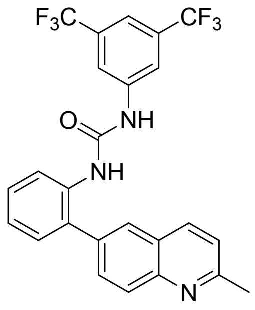

To a stirred solution of amine **4** (720 mg, 3 mmol) in 20 mL CH_2Cl_2 was added 780 μL (4.6 mmol) of 3,5-bis(trifluoromethyl)phenyl isocyanate. The reaction was allowed to stir for 18 h under argon atmosphere at room temperature. The solvent was removed *in vacuo* and the crude was chromatographed (silica gel, 9:1 CH_2Cl_2/EtOAc then 4:1 CH_2Cl_2/EtOAc) to obtain urea **5** (1.46 g, 98%). Analytical data for title compound: ^{1}H NMR (400 MHz, MeOD): δ 8.29 (d, *J* = 8.4 Hz, 1H), 8.05 (d, *J* = 8.8 Hz, 1H), 7.92 (d, *J* = 8.0 Hz, 3H), 7.83 – 7.74 (m, 1H), 7.54 – 7.36 (m, 3H), 7.31 (d, *J* = 7.5 Hz, 1H), 2.75 (q, *J* = 2.4 Hz, 3H). ^{13}C NMR (100 MHz, MeOD): δ 161.2, 155.3, 148.0, 143.1, 138.9, 138.5, 136.6, 135.8, 133.5, 133.2, 132.7, 132.0, 129.9, 129.5, 129.0, 128.5, 126.4, 125.5, 124.2, 119.4, 116.2, 25.0; HRMS (EI) calc. for $[C_{25}H_{17}F_6N_3O]^+$ 489.1276, found 489.1281; IR (NaCl) ν 3342, 3152, 1724, 1661, 1501, 1418, 1252, 1144, 1067 cm^{-1}

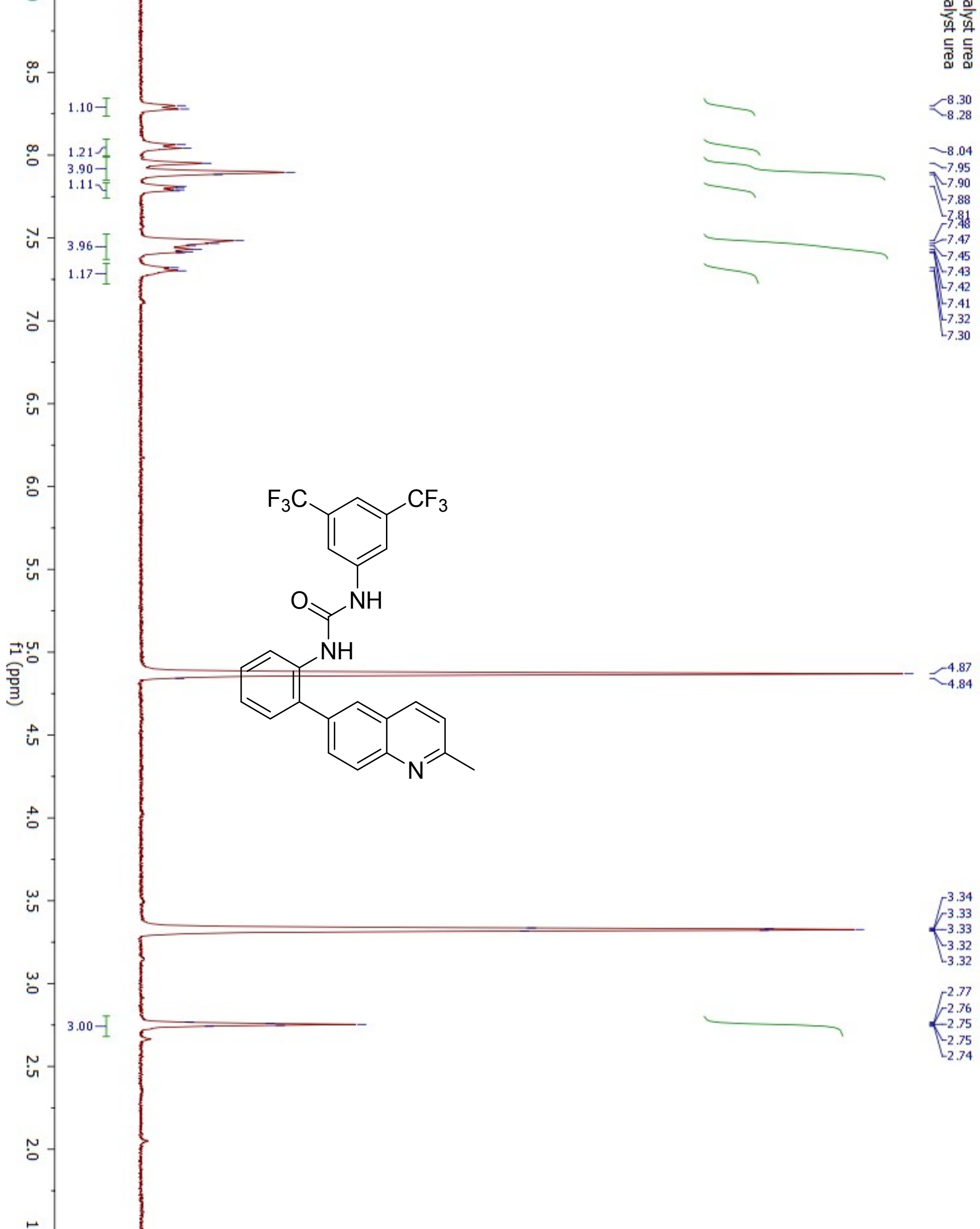

Figure S-3 ¹H-NMR of 5

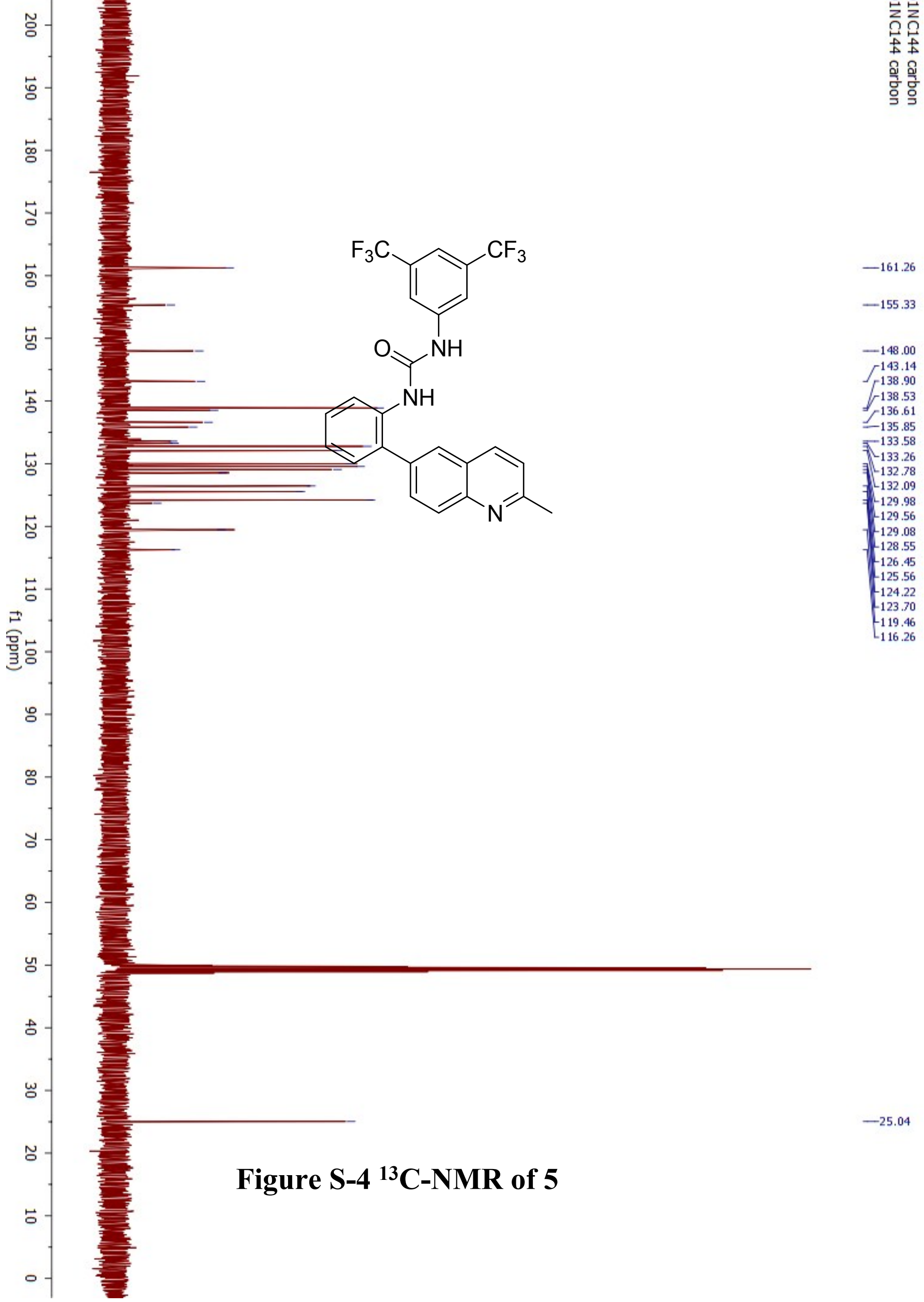

Figure S-4 ^{13}C-NMR of 5

1-(3,5-bis(trifluoromethyl)phenyl)-3-(2-(2-formylquinolin-6-yl)phenyl)urea (6):

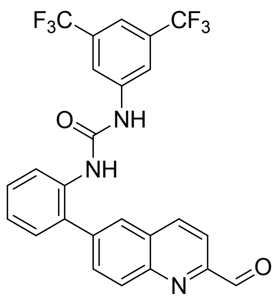

A 50 mL round bottom flask was charged with 1.46 g (3.0 mmol) of urea **5** and 410 mg (3.7 mmol) of SeO_2. *p*-Dioxane (15 mL) was added and the flask was fitted with a reflux condenser and heated to 80 °C for 2 h. The reaction was allowed to cool to room temperature before filtering through a pad of Celite. The solvent was removed *in vacuo* to yield pure aldehyde **6** as a solid (1.45 g, 97%). Analytical data for title compound: ^{1}H NMR (400 MHz, MeOD) δ 10.18 (s, 1H), 8.56 (d, *J* = 8.5 Hz, 1H), 8.33 (d, *J* = 8.7 Hz, 1H), 8.15 – 8.03 (m, 2H), 8.00 – 7.84 (m, 4H), 7.48 (d, *J* = 6.7 Hz, 3H), 7.36 (d, *J* = 8.1 Hz, 1H); ^{13}C NMR (insoluble); HRMS (EI) calc. for $[C_{26}H_{19}F_6N_3O_3]^+$ 535.1331, found 535.1332; IR (NaCl) ν 3251, 3123, 2467, 1711, 1622, 1379, 1133 cm^{-1}

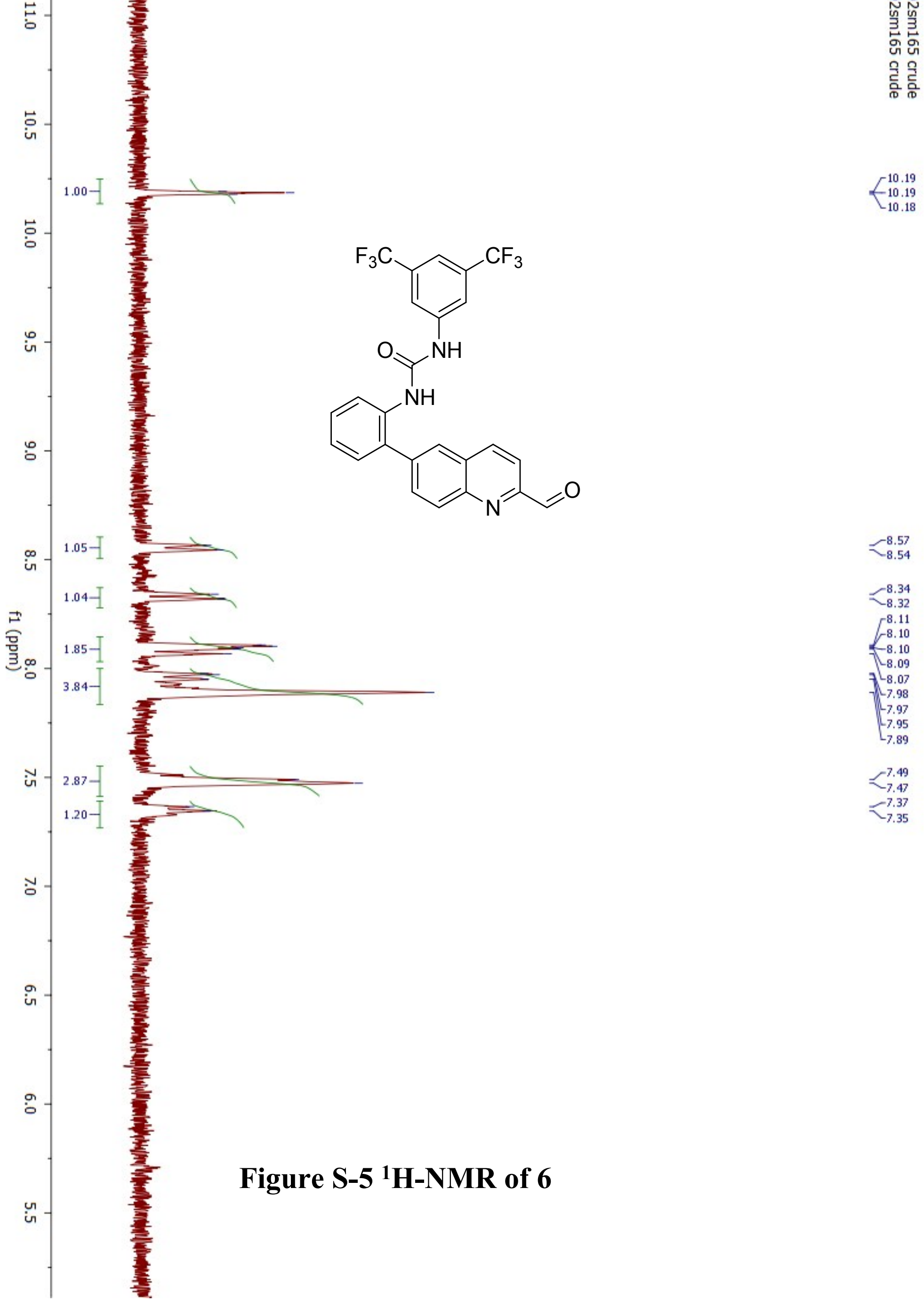

Figure S-5 ^{1}H-NMR of 6

(*S*)-1,1'-((2,2'-(((1-hydroxy-4-(methylthio)butan-2-yl)azanediyl)bis(methylene))bis(quinoline-6,2-diyl))bis(2,1-phenylene))bis(3-(3,5-bis(trifluoromethyl)phenyl)urea) (7):

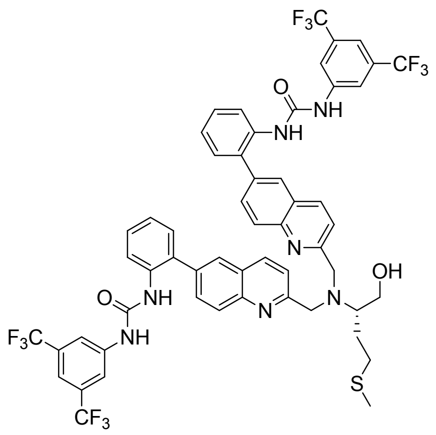

To a solution of 50 mg (0.37 mmol) L-methioninol in 2 mL MeOH at 0 °C was added a solution of 650 mg (1.3 mmol) of **6** in 10 mL MeOH. The mixture was stirred under argon for 10 min before 1.2 mL of AcOH and 80 mg (0.74 mmol) of 2-picoline borane were added at 0 °C. The reaction mixture was allowed to warm to room temperature and stirred for 16 h before the solvent was removed *in vacuo*. The crude reaction mixture was then cooled to 0 °C and treated with 10% aq. HCl (10 mL) and allowed to stir at room temperature for 30 minutes. The mixture was then made alkaline using 25% aq. Na_2CO_3 (50 mL). Product was then extracted into EtOAc (3 x 50 mL) and the organics were combined and washed with H_2O and brine before drying over Na_2SO_4. Solvent was removed *in vacuo* and crude product was chromatographed (silica gel, 1:1 EtOAc/CH_2Cl_2 then 10:1 CH_2Cl_2/MeOH) to obtain ligand **7** (386 mg, 94%). Analytical data for title compound: ^{1}H NMR (400 MHz, MeOD) δ 8.17 (d, *J* = 8.6 Hz, 2H), 8.02 (d, *J* = 8.7 Hz, 2H), 7.87 (d, *J* = 12.5 Hz, 9H), 7.76 (d, *J* = 8.5 Hz, 2H), 7.65 (d, *J* = 8.5 Hz, 2H), 7.51 – 7.39 (m, 5H), 7.37 (d, *J* = 7.1 Hz, 2H), 7.26 (t, *J* = 7.6 Hz, 2H), 4.25 (d, *J* = 3.1 Hz, 4H), 3.91 – 3.72 (m, 2H), 3.15 (bs, 1H), 2.73 – 2.58 (m, 2H), 2.07-1.97 (m, 4H), 1.80 (d, *J* = 7.5 Hz, 1H); ^{13}C NMR (100 MHz, MeOD) δ 162.8, 155.0, 147.4, 142.9, 138.6, 138.4, 136.3, 135.5, 133.7, 133.3, 133.0, 132.5, 131.9, 129.8, 129.3, 129.0, 126.2, 125.3, 123.5, 123.2, 120.7, 119.2, 116.0, 63.6, 63.3,

58.2, 32.8, 28.9, 15.4; HRMS (EI) calc. for $[C_{55}H_{43}F_{12}N_7O_3S]^+$ 1109.2956, found 1109.2952; IR

(NaCl) ν 3127, 1[illegible]

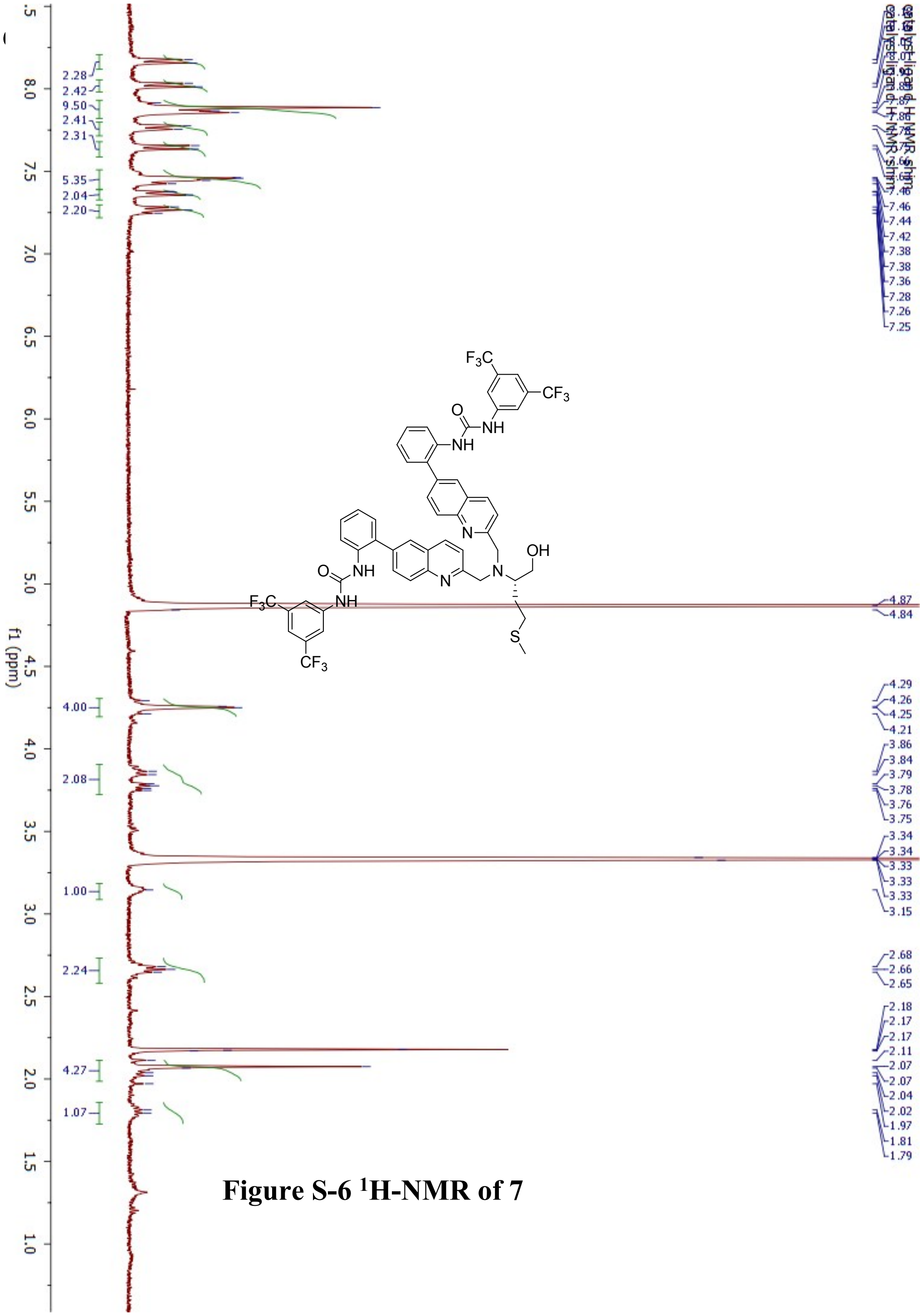

Figure S-6 ^{1}H-NMR of 7

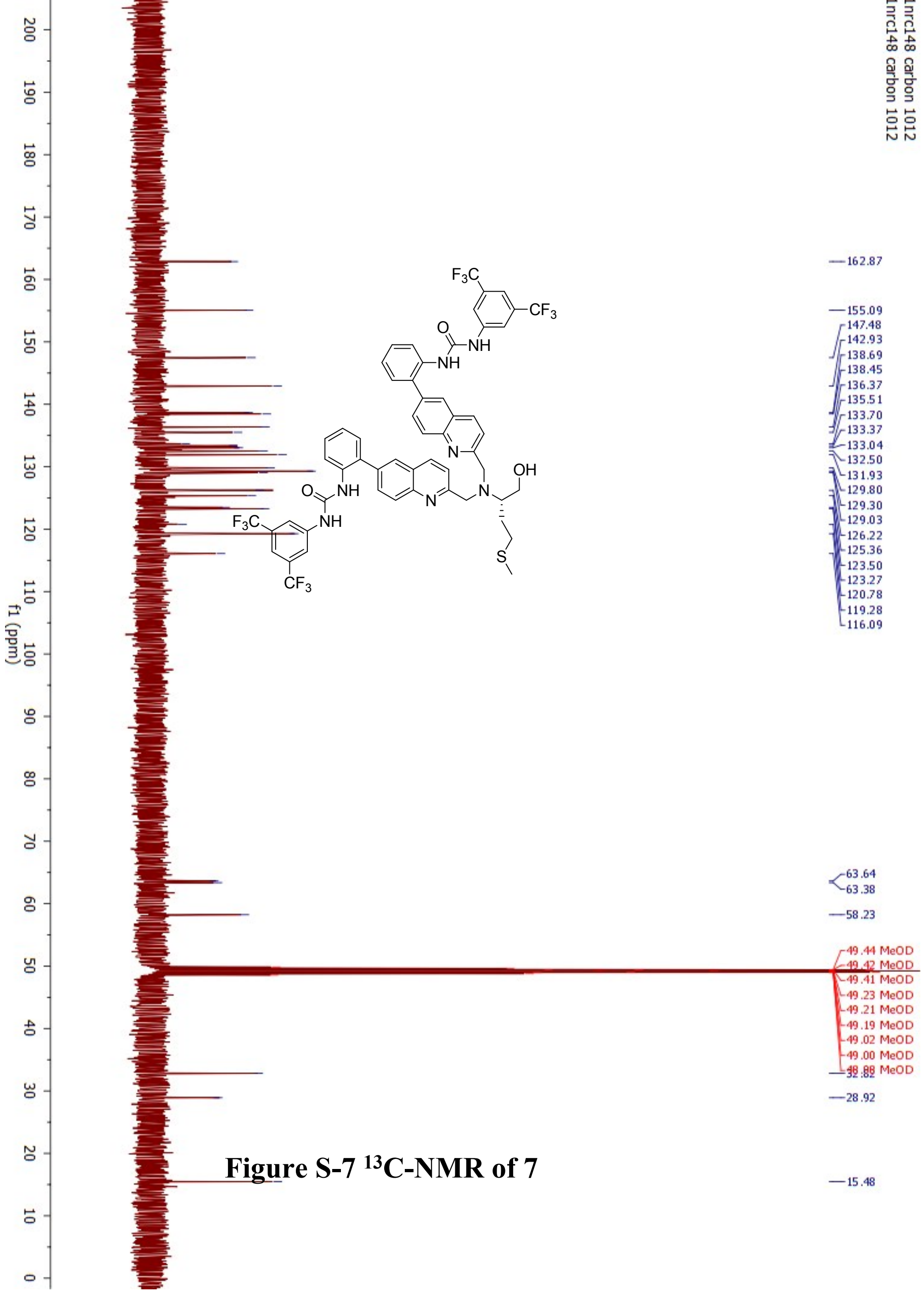

Figure S-7 ^{13}C-NMR of 7

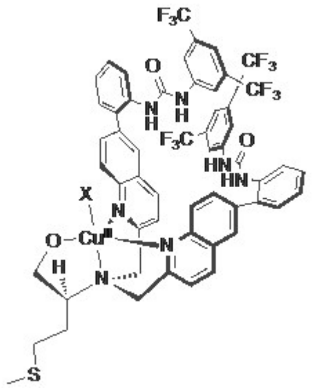

[Cu^{II}(**7**)](ClO_4)$_2$ ● 2(H_2O) (abb. as **Δ-1**): A solution of ligand **7** (100 mg, 0.09 mmol) in 210 μL of MeOH was stirred at room temperature for 5 min before a solution of Cu^{II}(ClO_4)$_2$● 6(H_2O) (40 mg, 1.0 mmol) in 300 μL of MeOH was added. The reaction mixture was stirred at room temperature for 1 h until a blue precipitate formed. The solid was obtained by filtration and washed with diethyl ether. The product was then recrystallized twice using acetonitrile to obtain **Δ-1** (101 mg, 80%) as a light blue solid. Analytical data for title compound: LRMS (EI): 1172 (M+), 622, 505 Anal. Calcd (mass%) for $C_{55}H_{47}Cl_2CuF_{12}N_7O_{13}S$: C, 46.90; H, 3.36; N, 6.96; Found C, 46.66; H, 2.99; N, 6.95.

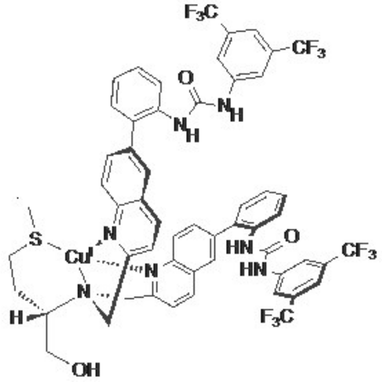

[Cu^{I}(**7**)]PF_6 (abb. as **Λ-1**): Compound **7** (197 mg, 0.18 mmol) was dissolved in 10 mL of CH_2Cl_2 and purged with N_2 before placing into a glovebox. Cu(MeCN)$_4$$PF_6$ (71 mg, 0.19 mmol) was added to the reaction mixture in the glovebox. The reaction was stirred for 30 minutes and the

solvent was removed *in vacuo* to obtain **Λ-1** (210 mg, 88%) as a yellow solid. LRMS (EI): 1172 (M+), 698, 505 : ^{1}H NMR (400 MHz, MeOD) δ 8.70 (d, *J* = 8.0 Hz, 1H), 8.52 (d, *J* = 8.0 Hz, 1H), 8.41 (d, *J* = 8.0 Hz, 1H), 8.36 (d, *J* = 8.0 Hz, 1H), 7.96-7.93 (m, 3H), 7.85 – 7.81 (m, 2H), 7.72-7.63 (m, 6H), 7.58 (d, *J* = 8.0 Hz, 1H), 7.46 (d, *J* = 8.0 Hz, 1H), 7.38 – 7.18 (m, 9H), 4.36 (d, *J* = 12.0 Hz, 1H), 4.0-3.99 (dd, *J* = 4.0 Hz, 1H), 3.96 (d, *J* = 4.0 Hz, 1H), 3.78-3.75 (m, 1H), 2.98-2.89 (m, 3H), 2.20 (s, 3H), 1.88-1.83 (m, 2H).

General procedure for enantioselective Michael addition of malonates to nitrostyrenes:

trans-β-Nitrostyrene (**8**) (50 mg, 0.34 mmol), diethyl malonate (**9**) (102 μL, 0.67 mmol), and acetonitrile (1 mL) were added to a scintillation vial equipped with a magnetic stir bar. Catalyst **Δ-1** (24 mg, 0.017 mmol) was added to the vial and stirred for 5 min until NEt_3 (4 μL, 0.034 mmol) was added to activate the reaction. The reaction was stirred for 24 h at room temperature at which point the solvent was removed *in vacuo*. The crude product was then chromatographed (silica gel, 15:85 EtOAc/hexane) to obtain (*S*)-**10** (58 mg, 55%). Spectral data for compounds (*S*)/(*R*)-**10a-h** matched those found in previous literature [1].

(*S*)-diethyl 2-(2-nitro-1-phenylethyl)malonate ((*S*)-10):

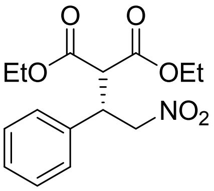

Analytical data for title compound: $[\alpha]_D^{25}$ = +5.8 (*c* 1.03, $CHCl_3$); ^{1}H NMR (400 MHz, $CDCl_3$) δ 7.29 – 7.10 (m, 5H), 4.88 – 4.73 (m, 2H), 4.21 – 4.07 (m, 3H), 3.92 (q, *J* = 7.1 Hz, 2H), 3.73 (d, *J* = 9.4 Hz, 1H), 1.21 – 1.13 (m, 3H), 0.96 (td, *J* = 7.0, 0.8 Hz, 3H); ^{13}C NMR (100 MHz, $CDCl_3$) δ 167.4, 166.7, 136.2, 128.9, 128.3, 128.0, 77.6, 62.1, 61.8, 54.9, 42.9, 13.9, 13.7. HRMS (EI) calc. for [$C_{15}H_{19}N$ [illegible]e/ethanol= 97/3, 1.00 mL/min, λ= [illegible].4 min, 72% ee).

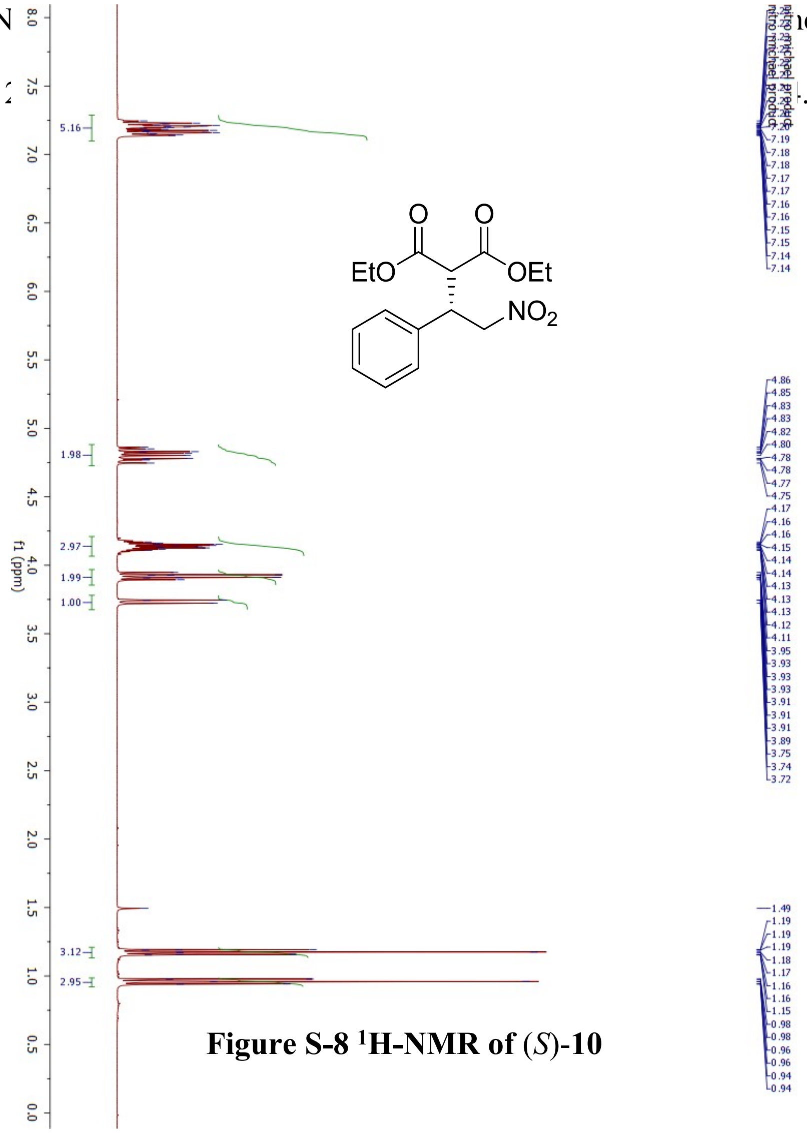

Figure S-8 ^{1}H-NMR of (*S*)-10

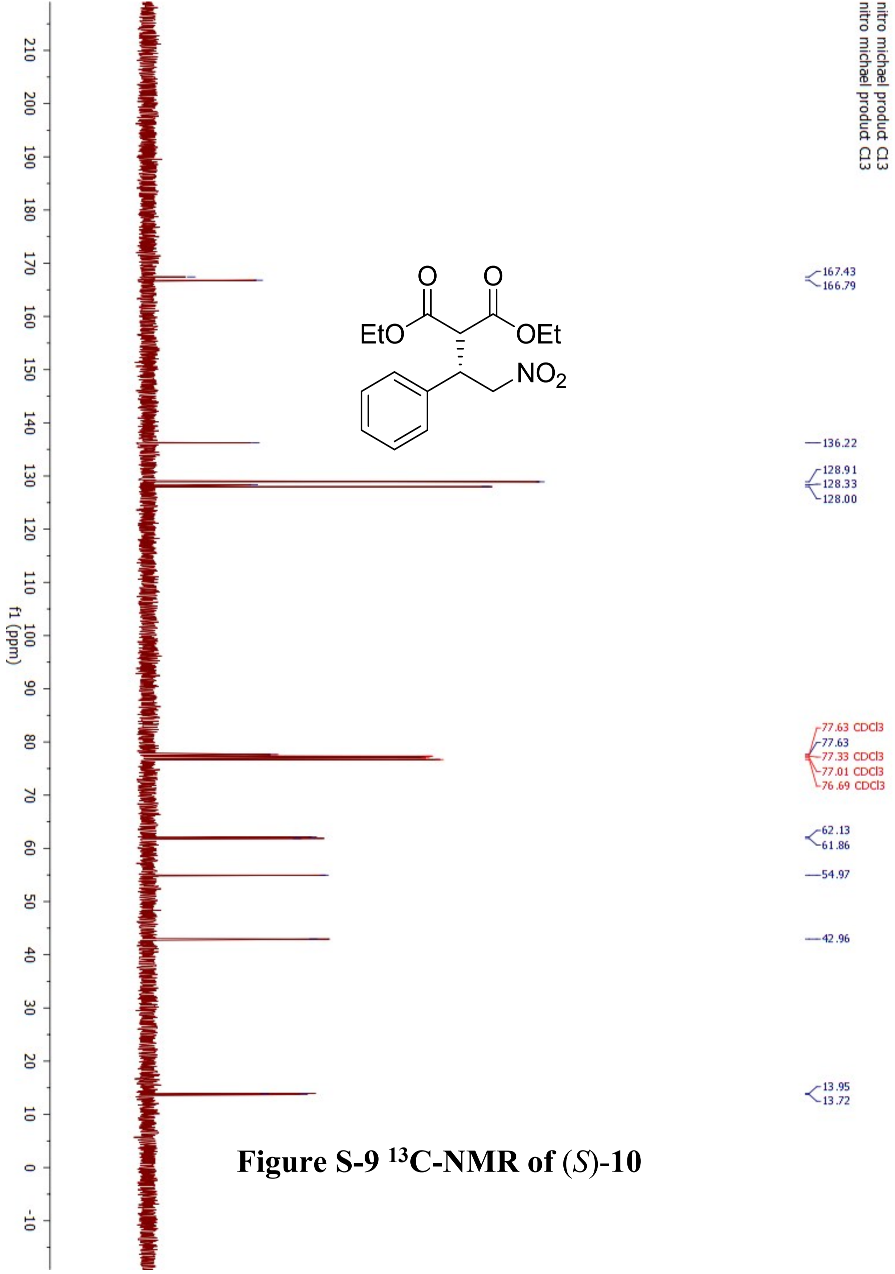

Figure S-9 ^{13}C-NMR of (*S*)-10

Data File C:\CHEM32\1\DATA\SM3088\SM3088 2011-07-15 18-10-38\071-0101.D
Sample Name: racemic

Acq. Operator : Shahab Seq. Line : 1
Acq. Instrument : Instrument 1 Location : Vial 71
Injection Date : 7/15/2011 6:11:47 PM Inj : 1
Inj Volume : 4 µl
Acq. Method : C:\Chem32\1\DATA\SM3088\SM3088 2011-07-15 18-10-38\SHAHAB.M
Last changed : 7/15/2011 6:10:36 PM by Shahab
Analysis Method : C:\CHEM32\1\METHODS\SHAHAB.M
Last changed : 7/15/2011 6:10:36 PM by Shahab
Method Info : 97:3 hexane/EtOH

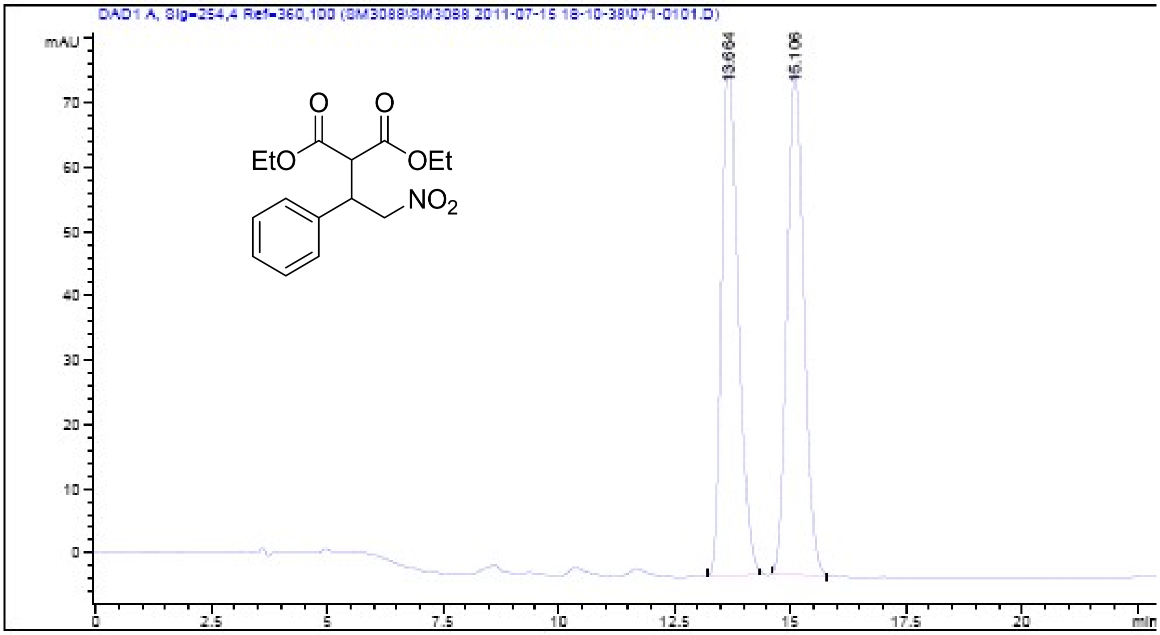

Area Percent Report

Sorted By : Signal
Multiplier : 1.0000
Dilution : 1.0000
Use Multiplier & Dilution Factor with ISTDs

Signal 1: DAD1 A, Sig=254,4 Ref=360,100

Peak #	RetTime [min]	Type	Width [min]	Area [mAU*s]	Height [mAU]	Area %
1	13.664	BB	0.3801	1984.53040	80.36646	50.0886
2	15.106	BB	0.3914	1977.50781	78.64338	49.9114
Totals :				3962.03821	159.00983	

Figure S-10 HPLC trace of racemic 10

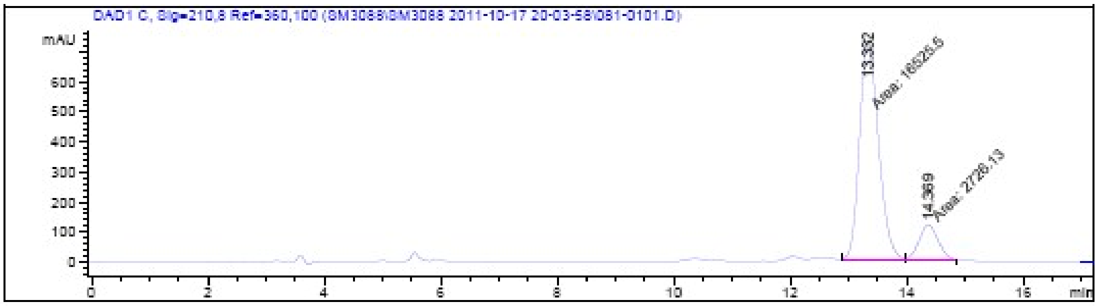

Area Percent Report

Sorted By : Signal
Multiplier : 1.0000
Dilution : 1.0000
Use Multiplier & Dilution Factor with ISTDs

Signal 1: DAD1 A, Sig=254,4 Ref=360,100

Signal 2: DAD1 C, Sig=210,8 Ref=360,100

Peak #	RetTime [min]	Type	Width [min]	Area [mAU*s]	Height [mAU]	Area %
1	13.332	MF	0.3809	1.65255e4	723.15735	85.8395
2	14.369	FM	0.3818	2726.12598	118.99001	14.1605

Figure S-11 HPLC trace of (*S*)-10

(*R*)-diethyl 2-(2-nitro-1-phenylethyl)malonate ((*R*)-10):

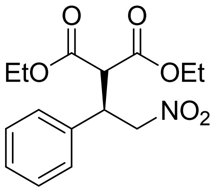

The same procedure was used as for the synthesis of (*S*)-31 except **Λ-1** (22 mg, 0.017 mmol) was used as the catalyst. Analytical data for title compound: (42 mg, 40%), $[\alpha]_D^{25}$ = -4.9 (*c* 1.00, $CHCl_3$); ^{1}H NMR (400 MHz, $CDCl_3$) δ 7.29 – 7.10 (m, 5H), 4.88 – 4.73 (m, 2H), 4.21 – 4.07 (m, 3H), 3.92 (q, *J* = 7.1 Hz, 2H), 3.73 (d, *J* = 9.4 Hz, 1H), 1.21 – 1.13 (m, 3H), 0.96 (td, *J* = 7.0, 0.8 Hz, 3H); ^{13}C NMR (100 MHz, $CDCl_3$) δ 167.43, 166.79, 136.22, 128.91, 128.33, 128.00, 77.63, 62.13, 61.86, 54.97, 42.96, 13.95, 13.72. HRMS (EI) calc. for $[C_{15}H_{19}NO_6]^+$ 309.1212, found 309.1196; HPLC (Chiralcel OD, hexane/ethanol= 97/3, 1.00 mL/min, λ= 210 nm, retention times: (S) (Minor) 13.3 min, (R) (Major) 14.4 min, 70% ee).

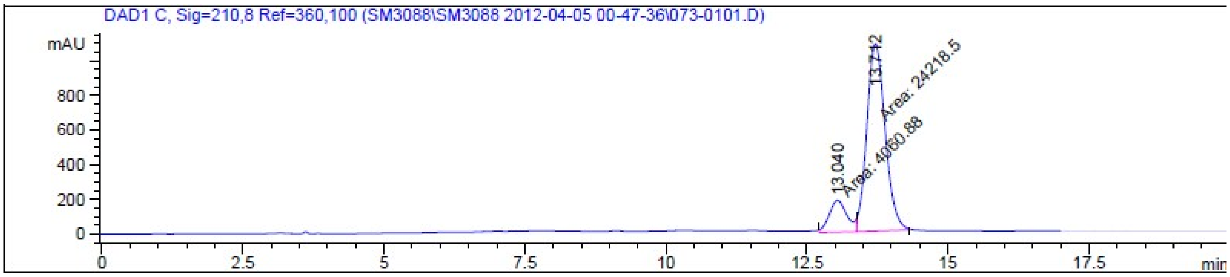

Signal 2: DAD1 C, Sig=210,8 Ref=360,100

Peak #	RetTime [min]	Type	Width [min]	Area [mAU*s]	Height [mAU]	Area %
1	13.040	MF	0.3675	4060.87866	184.18996	14.3599
2	13.712	FM	0.3739	2.42185e4	1079.56470	85.6401
Totals :				2.82793e4	1263.75465	

Figure S-12 HPLC trace of (*R*)-10

(*S*)-diethyl 2-(2-nitro-1-(p-tolyl)ethyl)malonate ((*S*)-10a):

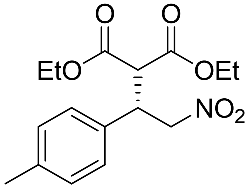

Δ-1 used as catalyst. Analytical data for title compound: (74 mg, 67%), $[\alpha]_D^{25}$ = +3.8 (*c* 0.52, $CHCl_3$); ^{1}H NMR (400 MHz, $CDCl_3$) δ 7.06 – 7.02 (m, 4H), 4.79 (dd, *J* = 11.6, 5.2 Hz, 1H), 4.77 (dd, *J* = 9.2, 6.2 Hz,1H), 4.15 – 4.10 (m, 3H), 3.94 (q, *J* = 7.2 Hz, 2H), 3.72 (d, *J* = 9.6 Hz, 1H), 2.21 (s, 3H), 1.21 (t, *J* = 7.1 Hz, 3H), 1.00 (t, *J* = 7.3 Hz, 3H); ^{13}C NMR (100 MHz, $CDCl_3$) δ 167.5, 166.9, 138.1, 133.1, 129.6, 127.8, 77.8, 62.1, 61.9, 55.1, 42.6, 21.1, 13.9, 13.7. HRMS (EI) calc. for $[C_{16}H_{21}NNaO_6]^+$ 346.1267, found 346.1268; HPLC (Chiralcel OD, hexane/isopropanol= 95/5, 0.8 mL/min, λ= 210 nm, retention times: (R) (Minor) 18.7 min, (S) (Major) 17.1 min, 70% ee).

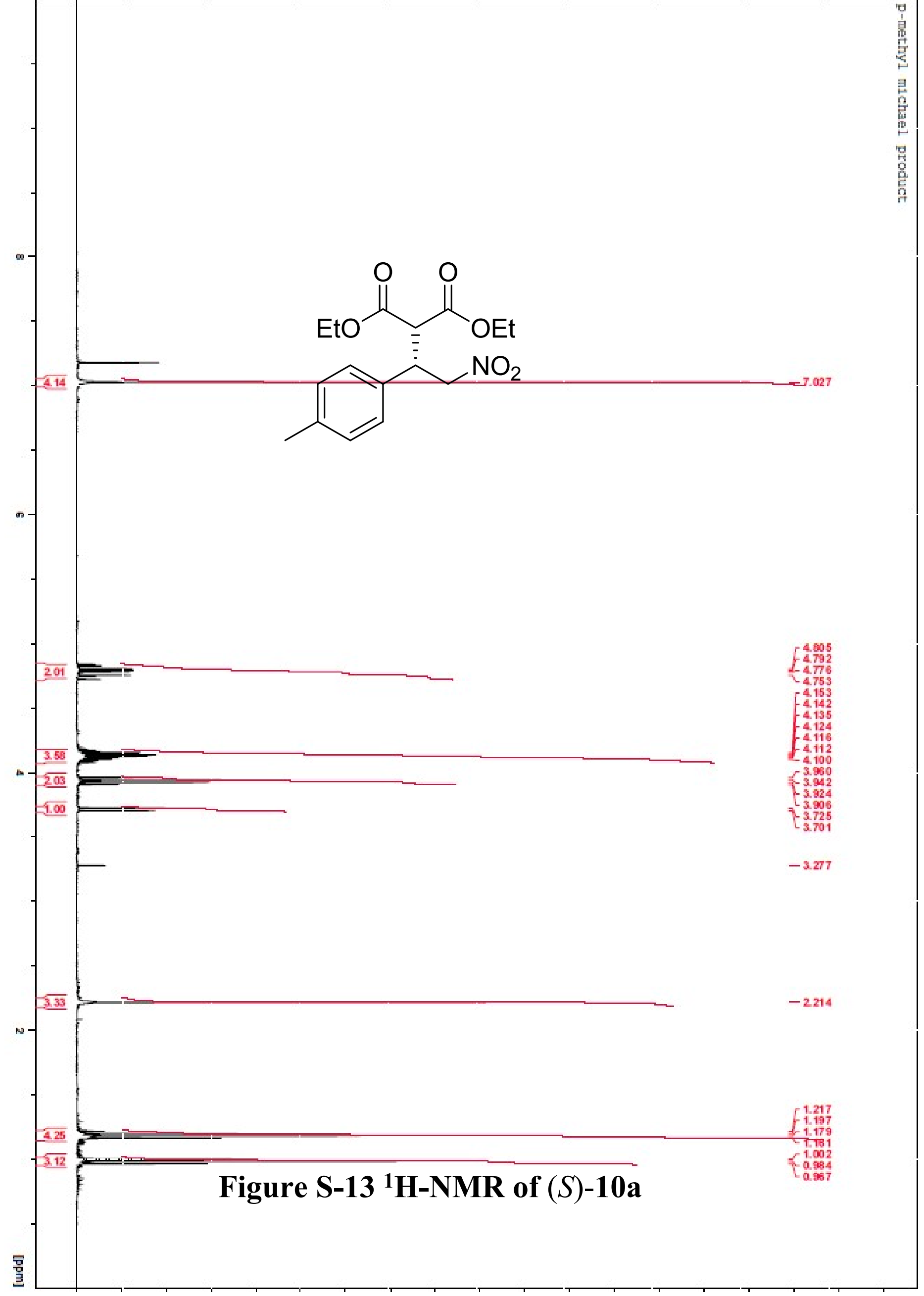

Figure S-13 ¹H-NMR of (*S*)-10a

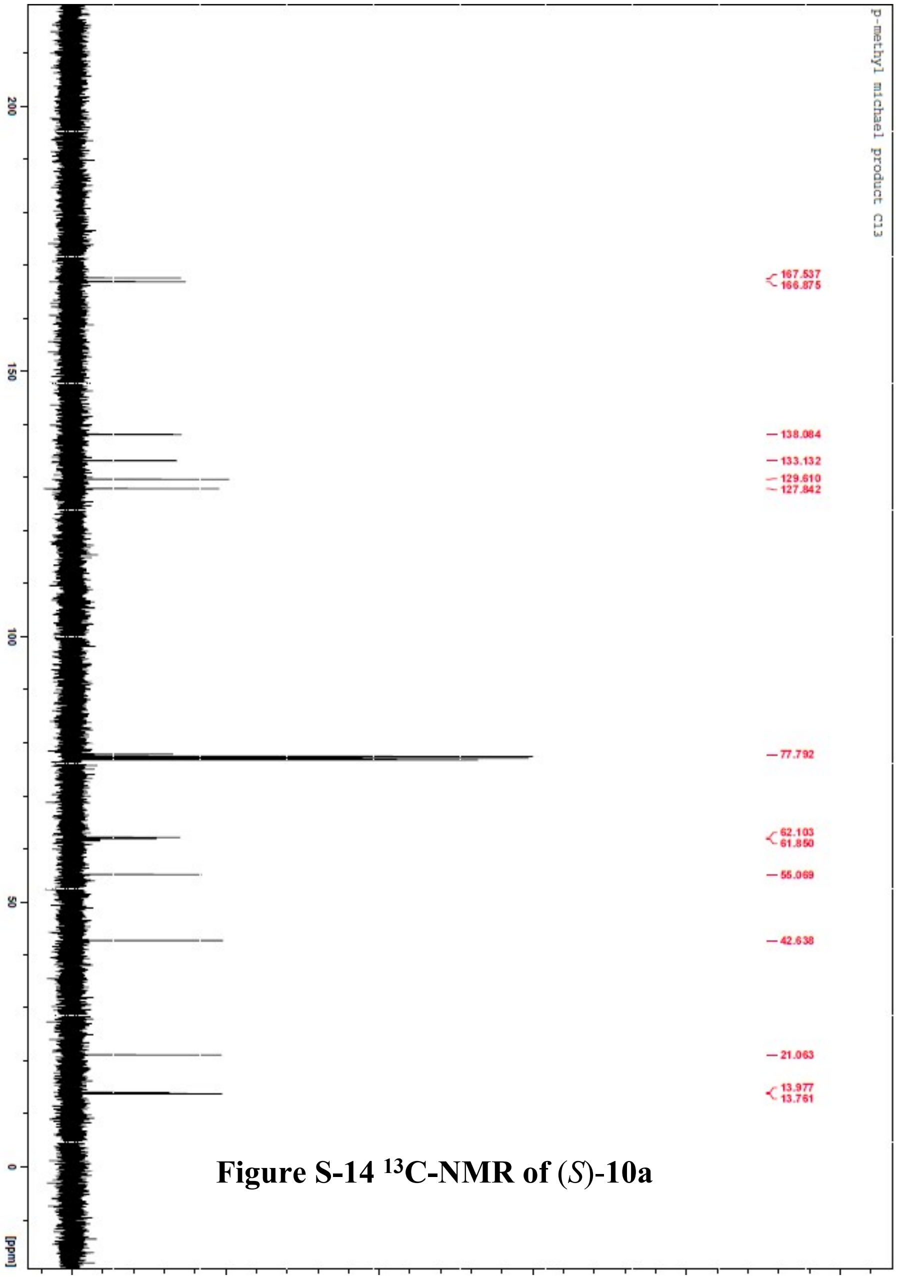

Figure S-14 ^{13}C-NMR of (*S*)-10a

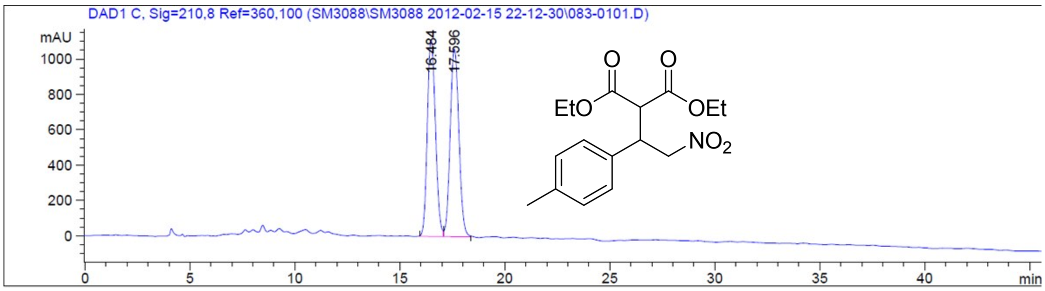

Signal 2: DAD1 C, Sig=210,8 Ref=360,100

Peak #	RetTime [min]	Type	Width [min]	Area [mAU*s]	Height [mAU]	Area %
1	16.484	BV	0.4272	3.00606e4	1112.78906	49.5339
2	17.596	VB	0.4526	3.06263e4	1061.36938	50.4661
Totals :				6.06869e4	2174.15845	

Figure S-15 HPLC trace of racemic 10a

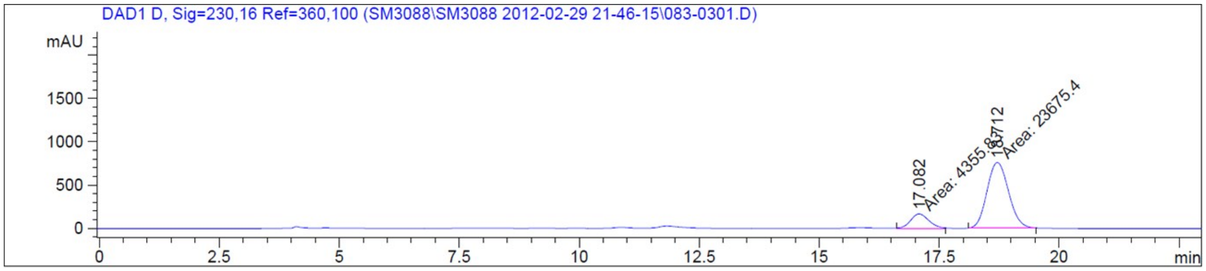

Signal 3: DAD1 D, Sig=230,16 Ref=360,100

Peak #	RetTime [min]	Type	Width [min]	Area [mAU*s]	Height [mAU]	Area %
1	17.082	MM	0.4381	4355.83057	165.71893	15.5392
2	18.712	MM	0.5206	2.36754e4	757.89734	84.4608
Totals :				2.80312e4	923.61627	

Figure S-16 HPLC trace of (*S*)-10a

(*R*)-diethyl 2-(2-nitro-1-(p-tolyl)ethyl)malonate ((*R*)-10a):

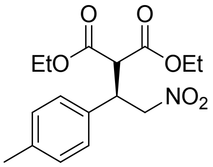

Λ-1 used as catalyst. Analytical data for title compound: (61 mg, 55%), $[\alpha]_D^{25}$ = -2.3 (*c* 1.06, $CHCl_3$); HPLC (Chiralcel OD, hexane/isopropanol= 95/5, 0.8 mL/min, λ= 210 nm, retention times: (R) (Major) 14.9 min, (S) (Minor) 16.7 min, 67% ee).

Signal 3: DAD1 D, Sig=230,16 Ref=360,100

Peak #	RetTime [min]	Type	Width [min]	Area [mAU*s]	Height [mAU]	Area %
1	14.921	VV	0.4160	2.09741e4	784.34772	83.2229
2	16.694	VV	0.4440	4228.22021	145.08745	16.7771
Totals :				2.52023e4	929.43517	

Figure S-17 HPLC trace of (*R*)-10a

(*S*)-diethyl 2-(1-(4-methoxyphenyl)-2-nitroethyl)malonate ((*S*)-10b):

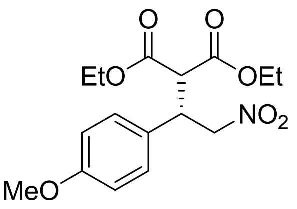

Δ-1 used as catalyst. Analytical data for title compound: (53 mg, 46%), $[\alpha]_D^{25}$ = +2.0 (*c* 0.5, $CHCl_3$); ^1H NMR (400 MHz, $CDCl_3$) δ 7.07 (d, *J* = 8.4 Hz, 2H), 6.76 (d, *J* = 8.8 Hz, 2H), 4.79 (dd, *J* = 12.8, 8.0 Hz, 1H), 4.72 (dd, 1H, *J* = 12.4, 9.2 Hz), 4.16 – 4.08 (m, 3H), 3.94 (q, *J* = 6.8 Hz, 2H), 3.71-3.68 (m, 4H), 1.18 (t, *J* = 7.2 Hz, 3H), 0.99 (t, *J* = 7.2 Hz, 3H); ^{13}C NMR (100 MHz, $CDCl_3$) δ 167.5, 166.9, 159.4, 129.2, 128.0, 114.3, 77.9, 62.1, 61.8, 55.2, 55.1, 42.3, 13.9, 13.8. HRMS (EI) calc. for $[C_{16}H_{21}NNaO_7]^+$ 362.1216, found 362.1225; HPLC (Chiralcel OD, hexane/isopropanol= 95/5, 0.8 mL/min, λ= 210 nm, retention times: (R) (Minor) 26.0 min, (S) (Major) 28.4 mi

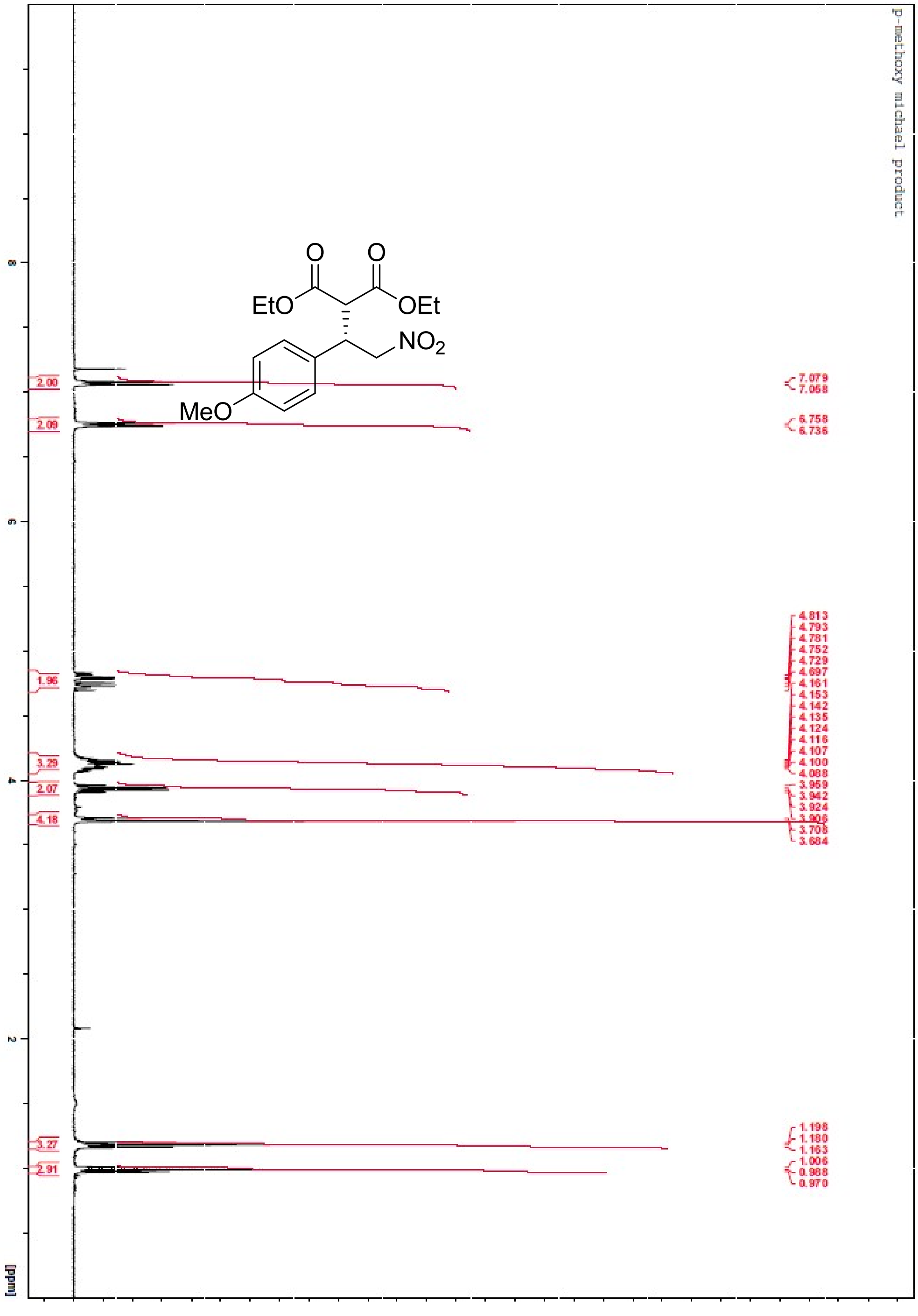

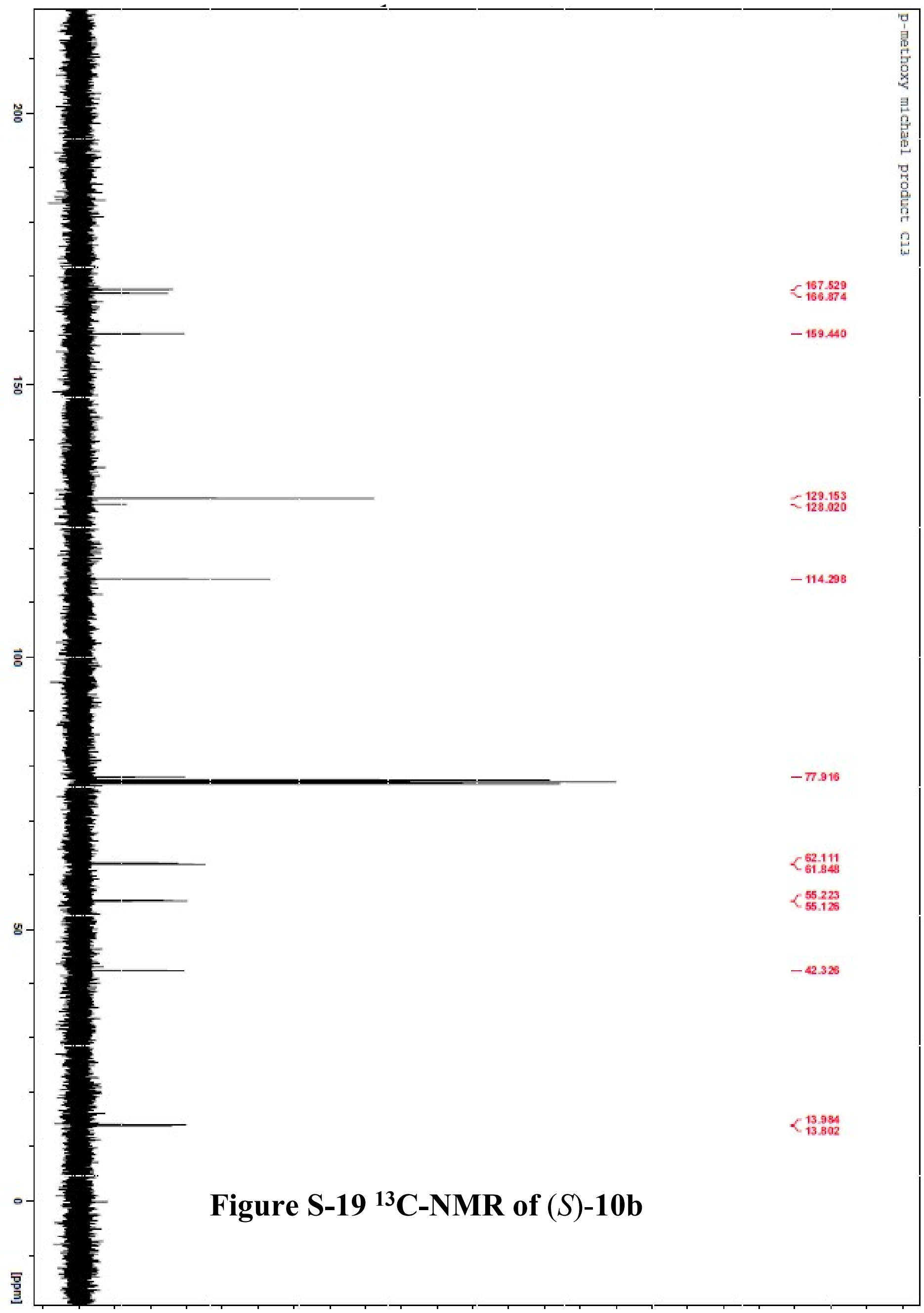

Figure S-19 ^{13}C-NMR of (*S*)-10b

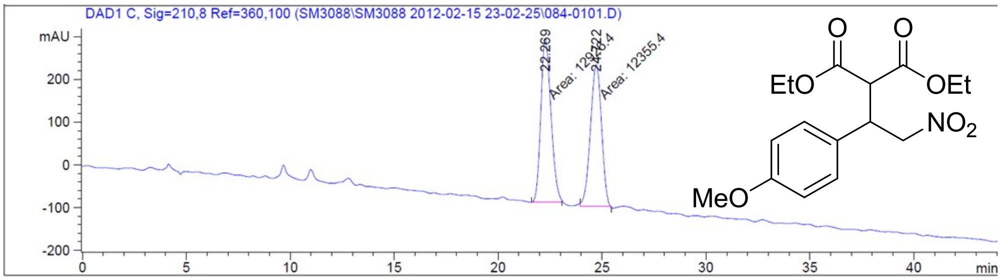

Signal 2: DAD1 C, Sig=210,8 Ref=360,100

Peak #	RetTime [min]	Type	Width [min]	Area [mAU*s]	Height [mAU]	Area %
1	22.269	MM	0.5646	1.29264e4	381.57730	51.1293
2	24.722	MM	0.6199	1.23554e4	332.19260	48.8707
Totals :				2.52818e4	713.76990	

Figure S-20 HPLC trace of racemic 10b

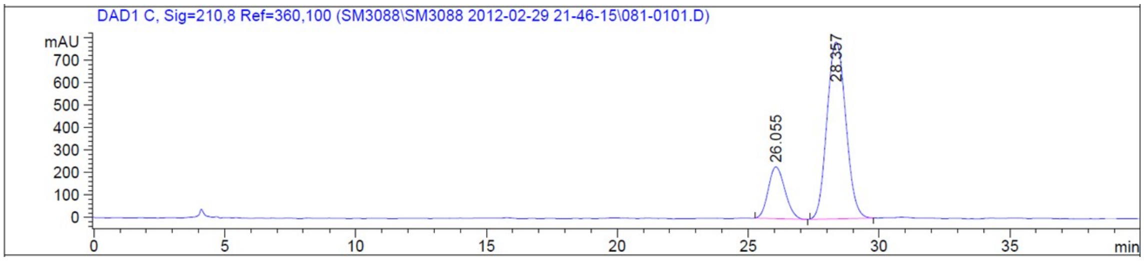

Signal 2: DAD1 C, Sig=210,8 Ref=360,100

Peak #	RetTime [min]	Type	Width [min]	Area [mAU*s]	Height [mAU]	Area %
1	26.055	BB	0.6983	1.01666e4	229.71080	20.5318
2	28.357	BB	0.7978	3.93496e4	786.29913	79.4682
Totals :				4.95162e4	1016.00993	

Figure S-21 HPLC trace of (*S*)-10b

(*R*)-diethyl 2-(1-(4-methoxyphenyl)-2-nitroethyl)malonate ((*R*)-10b):

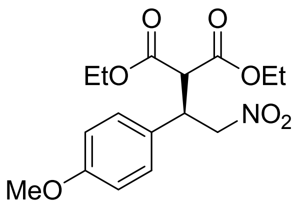

Λ-1 used as catalyst. Analytical data for title compound: (23 mg, 20%), $[\alpha]_D^{25}$ = -5.5 (*c* 0.4, $CHCl_3$); HPLC (Chiralcel OD, hexane/isopropanol= 95/5, 0.8 mL/min, λ= 210 nm, retention times: (R) (Major) 22.6 min, (S) (Minor) 25.7 min, 73% ee).

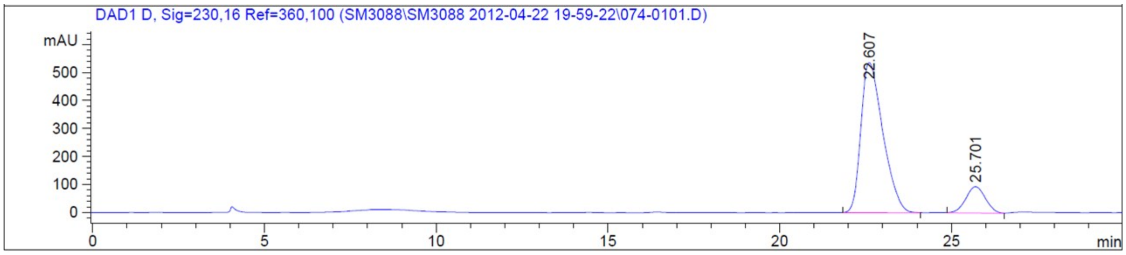

Signal 3: DAD1 D, Sig=230,16 Ref=360,100

Peak #	RetTime [min]	Type	Width [min]	Area [mAU*s]	Height [mAU]	Area %
1	22.607	BB	0.6811	2.38597e4	536.24268	86.5882
2	25.701	BV	0.6149	3695.68262	93.94788	13.4118
Totals :				2.75554e4	630.19056	

Figure S-22 HPLC trace of (*R*)-10b

(*S*)-diethyl 2-(1-(2,3-dimethoxyphenyl)-2-nitroethyl)malonate ((*S*)-10c):

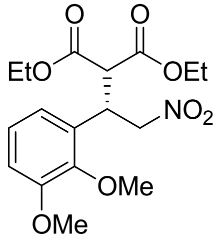

Δ-1 used as catalyst. Analytical data for title compound: (121 mg, 97%), $[\alpha]_D^{25}$ = +4.9 (*c* 0.85, $CHCl_3$); ^1H NMR (400 MHz, $CDCl_3$) δ 6.87 (t, *J* = 8.0 Hz, 1H), 6.76 (d, *J* = 8.0 Hz, 1H), 6.65 (d, *J* = 7.6 Hz, 1H), 4.91 (dd, *J* = 13.2, 9.8 Hz, 1H), 4.83 (dd, 1H, *J* = 13.2, 4.2 Hz), 4.41 – 4.36 (m, 3H), 4.12 - 4.08 (m, 2H), 3.97 - 3.90 (m, 3H), 3.86 (s, 3H), 3.74 (s, 3H). 1.15 (t, *J* = 7.6 Hz, 3H), 0.97 (t, *J* = 6.8 Hz, 3H); ^{13}C NMR (100 MHz, $CDCl_3$) δ 167.8, 167.2, 152.7, 129.3, 123.8, 120.9, 112.7, 76.6, 61.9, 61.7, 60.7, 55.7, 53.6, 38.6, 13.9, 13.8. HRMS (EI) calc. for $[C_{17}H_{24}NO_8]^+$ 370.1502, found 370.1530; HPLC (Chiralcel OD, hexane/isopropanol= 95/5, 0.8 mL/min, λ= 210 nm, retention times: (S) (Major) 16.1 min, (R) (Minor) 19.0 min, 48% ee).

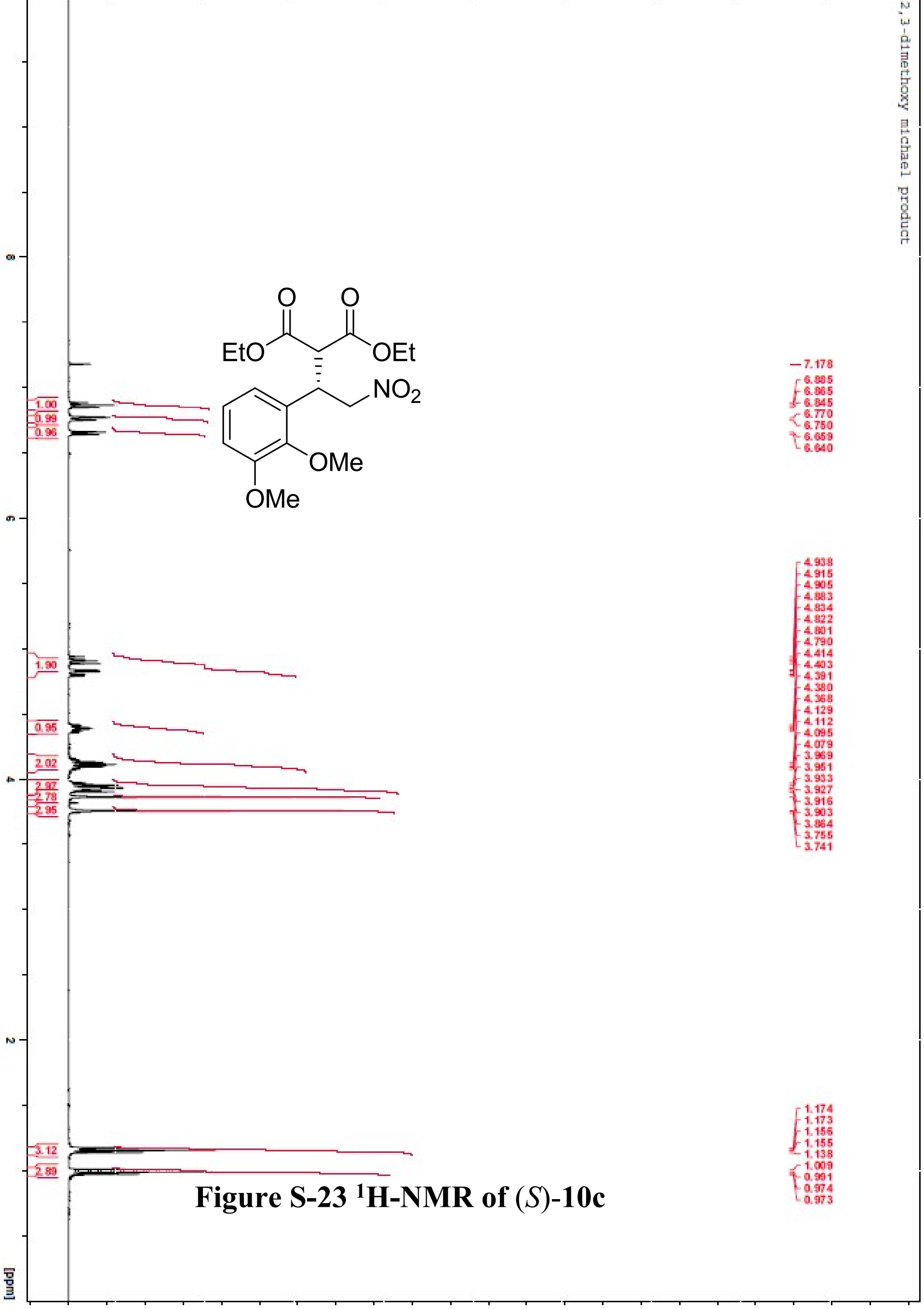

Figure S-23 ^{1}H-NMR of (*S*)-10c

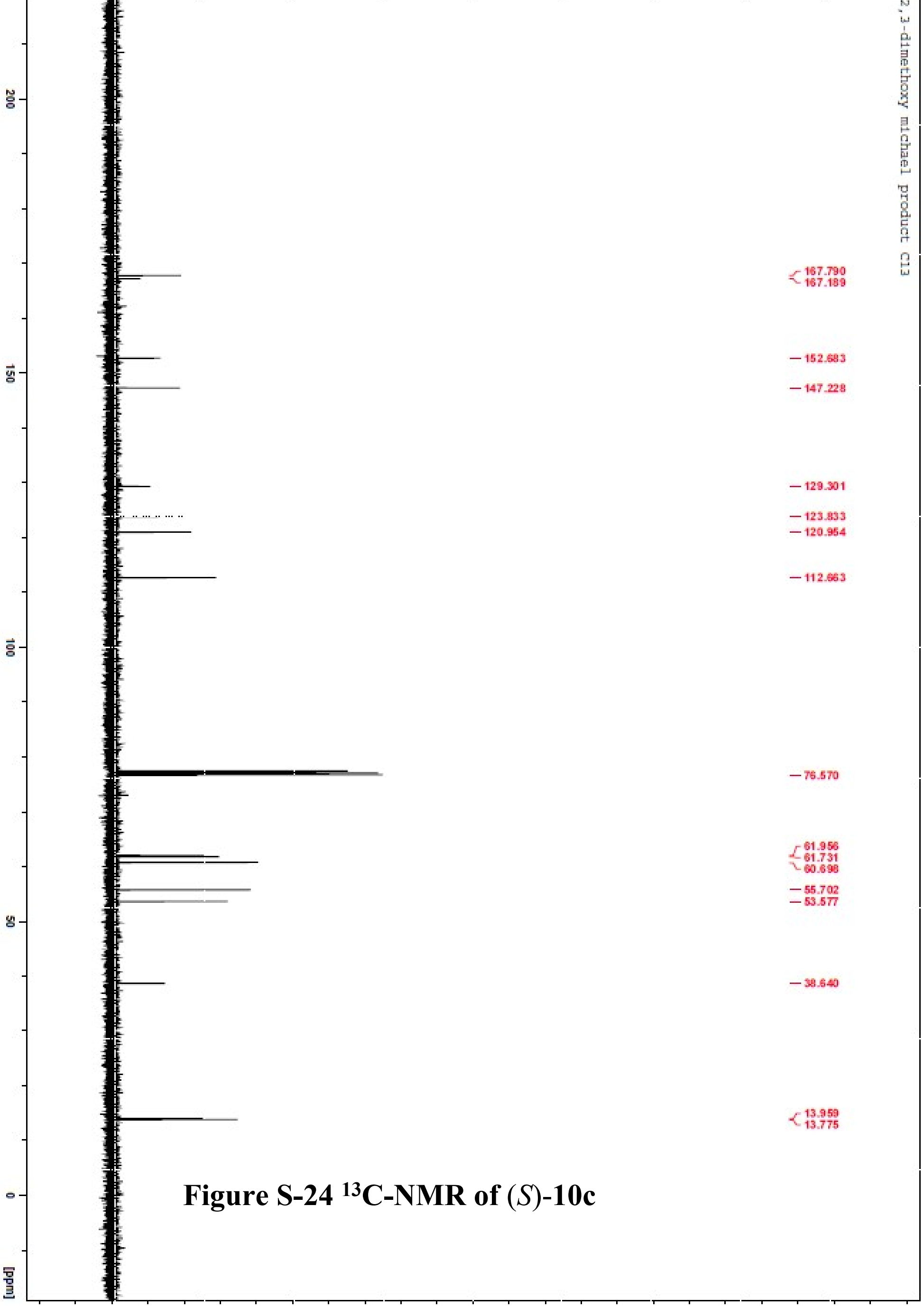

Figure S-24 ^{13}C-NMR of (*S*)-10c

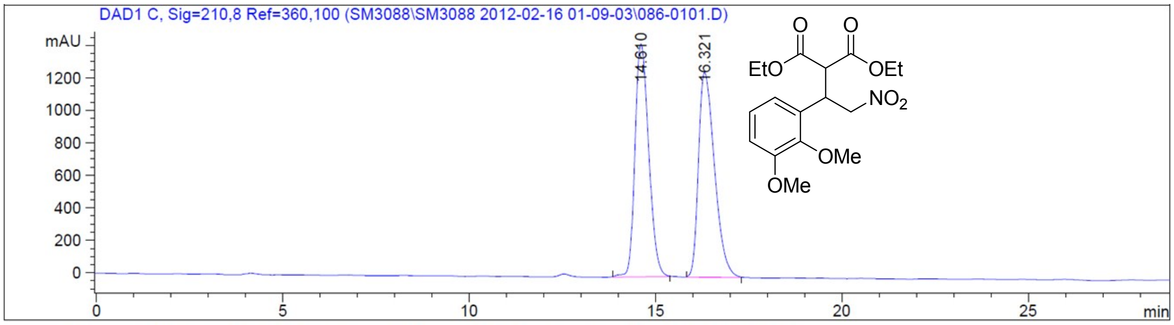

Signal 2: DAD1 C, Sig=210,8 Ref=360,100

Peak #	RetTime [min]	Type	Width [min]	Area [mAU*s]	Height [mAU]	Area %
1	14.610	BB	0.4046	3.69644e4	1434.53784	49.4978
2	16.321	BB	0.4629	3.77144e4	1261.07422	50.5022
Totals :				7.46788e4	2695.61206	

Figure S-25 HPLC trace of racemic 10c

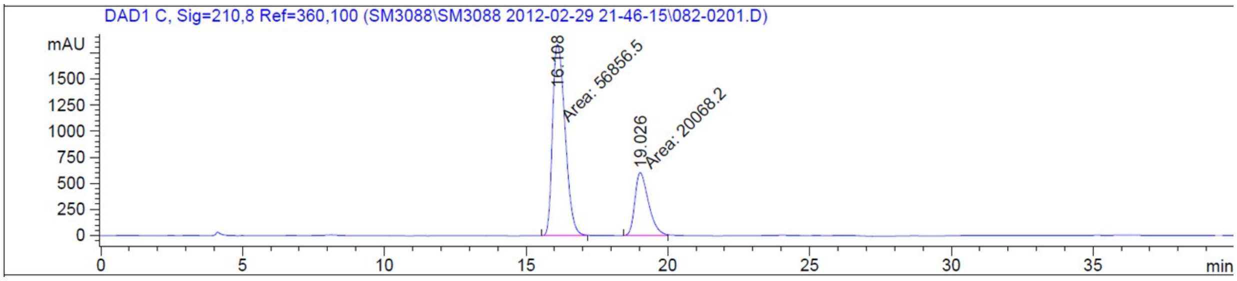

Signal 2: DAD1 C, Sig=210,8 Ref=360,100

Peak #	RetTime [min]	Type	Width [min]	Area [mAU*s]	Height [mAU]	Area %
1	16.108	MM	0.5160	5.68565e4	1836.35291	73.9119
2	19.026	MM	0.5556	2.00682e4	602.04913	26.0881

Figure S-26 HPLC trace of (*S*)-10c(*R*)-diethyl 2-(1-(2,3-dimethoxyphenyl)-2-nitroethyl)malonate ((*R*)-10c):

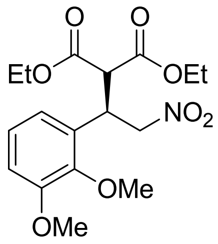

Λ-1 used as catalyst. Analytical data for title compound: (122 mg, 98%), $[\alpha]_D^{25}$ = -10.9 (*c* 0.87, $CHCl_3$); HPLC (Chiralcel OD, hexane/isopropanol= 95/5, 0.8 mL/min, λ= 210 nm, retention times: (S) (Minor) 14.3 min, (R) (Major) 16.0 min, 57% ee).

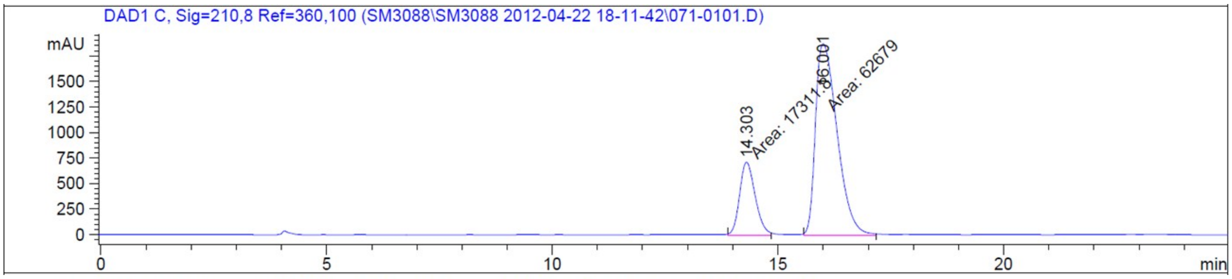

Signal 2: DAD1 C, Sig=210,8 Ref=360,100

Peak #	RetTime [min]	Type	Width [min]	Area [mAU*s]	Height [mAU]	Area %
1	14.303	MM	0.4020	1.73118e4	717.68866	21.6422
2	16.001	MM	0.5567	6.26790e4	1876.55286	78.3578

Figure S-27 HPLC trace of (*R*)-10c

(*S*)-diethyl 2-(1-(4-bromophenyl)-2-nitroethyl)malonate ((*S*)-10d):

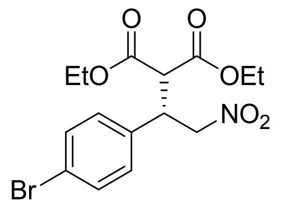

Δ-1 used as catalyst. Analytical data for title compound: (59 mg, 45%), $[\alpha]_D^{25}$ = +1.3 (*c* 0.5, $CHCl_3$); ^{1}H NMR (400 MHz, $CDCl_3$) δ 7.36 (d, *J* = 8.4 Hz, 2H), 7.04 (d, *J* = 8.4 Hz, 2H), 4.82 (dd, *J* = 13.2, 8.4 Hz, 1H), 4.78 (dd, *J* = 13.2, 9.4 Hz, 1H), 4.16 - 4.10 (m, 3H), 3.98 - 3.93 (m, 2H), 3.69 (d, *J* = 9.2 Hz, 1H). 1.17 (t, *J* = 7.2 Hz, 3H), 0.99 (t, *J* = 7.1 Hz, 3H); ^{13}C NMR (100 MHz, $CDCl_3$) δ 167.2, 166.6, 132.1, 129.7, 123.8, 62.3, 62.1, 54.7, 42.4, 13.9, 13.8. HRMS (EI) calc. for $[C_{15}H_{18}BrNNaO_6]^+$ 410.0215, found 410.0219; HPLC (Chiralcel AD-H, hexane/isopropanol= 90/10, 0.8 mL/min, λ= 210 nm, retention times: (R) (Minor) 25.4 min, (S) (Major) 64.1 min, 24% ee).

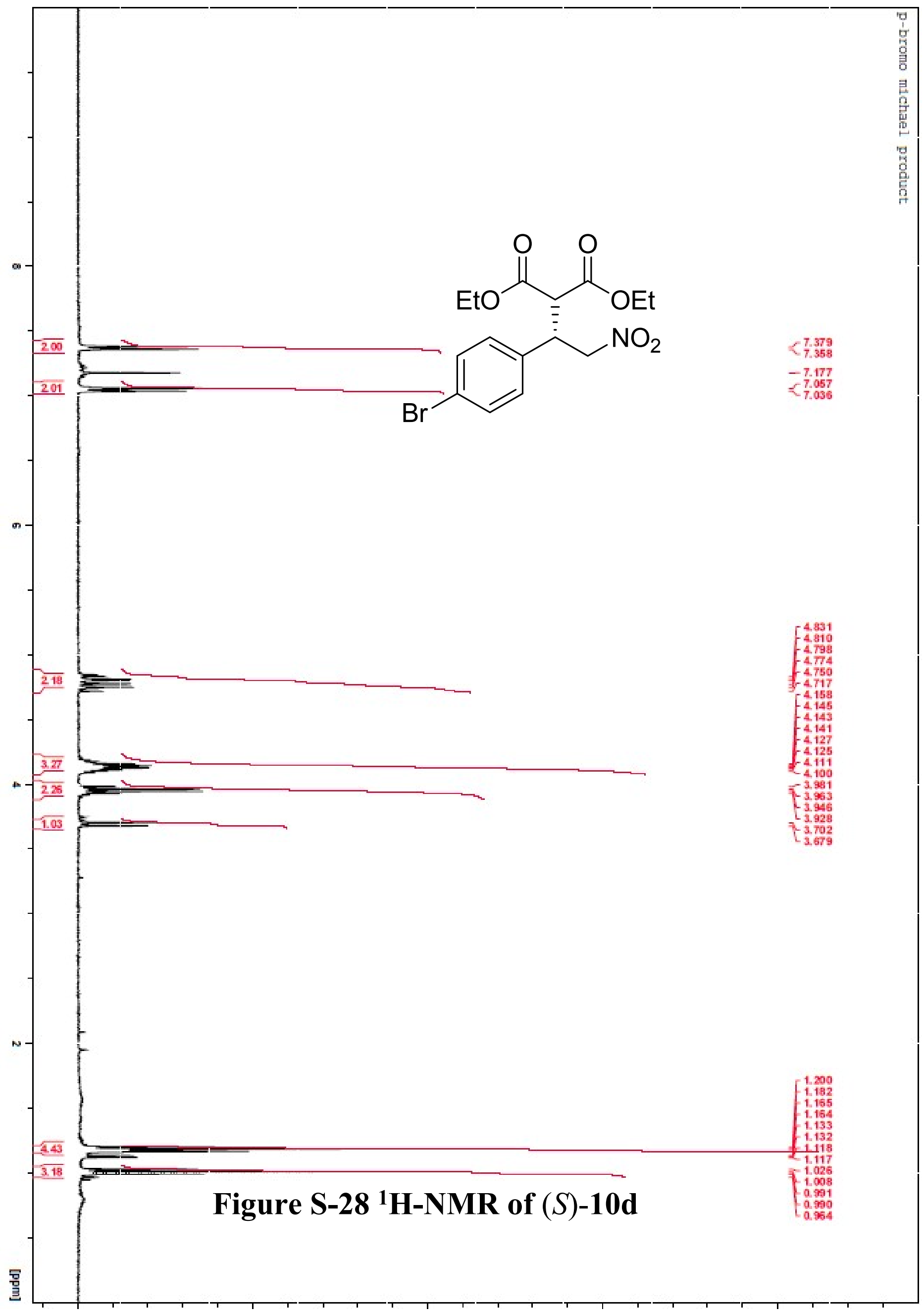

Figure S-28 ^{1}H-NMR of (*S*)-10d

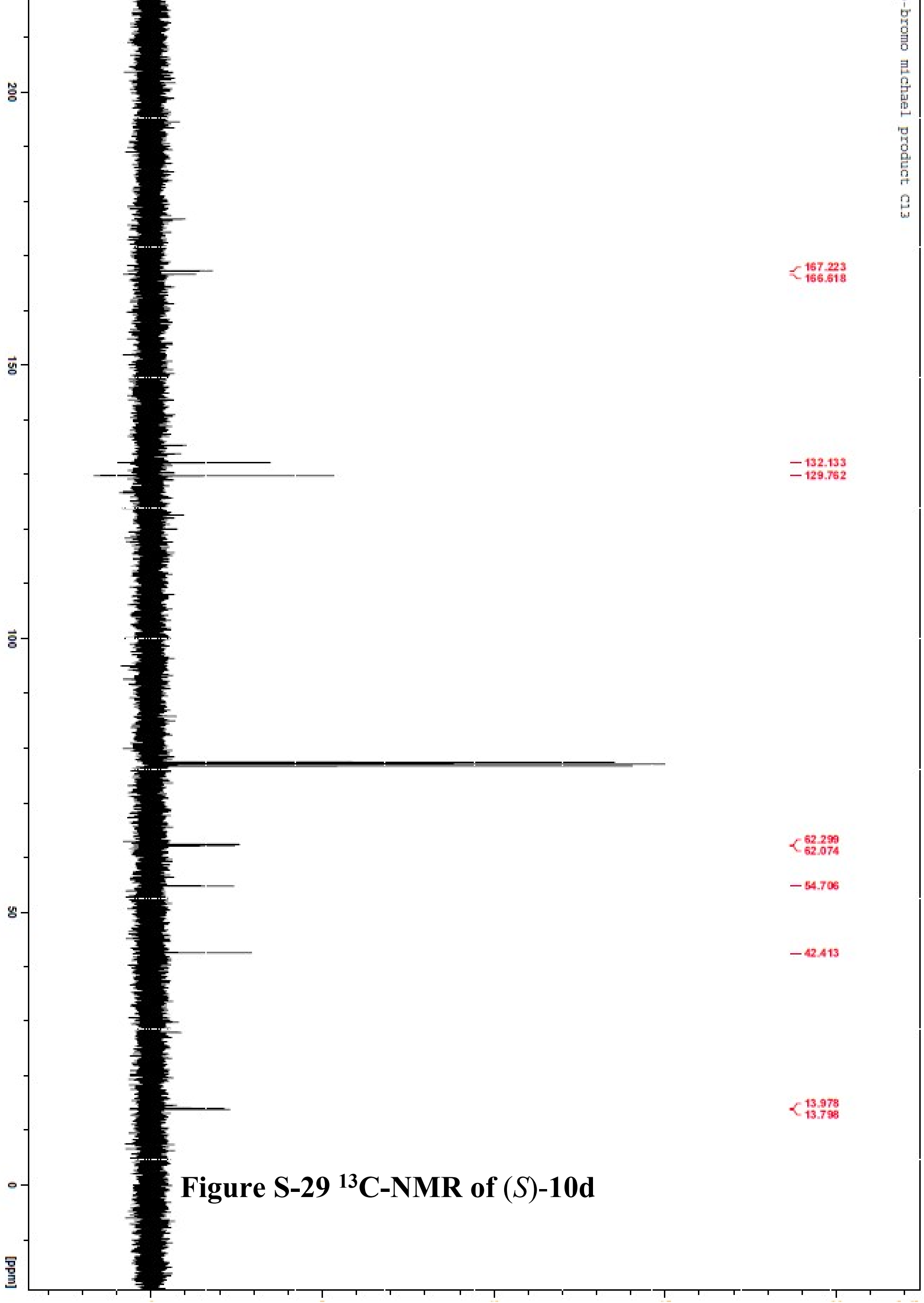

Figure S-29 ^{13}C-NMR of (*S*)-10d

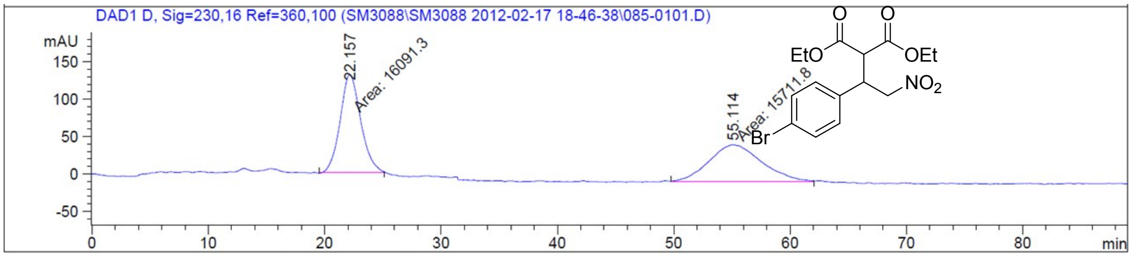

Signal 3: DAD1 D, Sig=230,16 Ref=360,100

Peak #	RetTime [min]	Type	Width [min]	Area [mAU*s]	Height [mAU]	Area %
1	22.157	MM	2.0478	1.60913e4	130.96414	50.5967
2	55.114	MM	5.2701	1.57118e4	49.68838	49.4033
Totals :				3.18030e4	180.65252	

Figure S-30 HPLC trace of racemic 10d

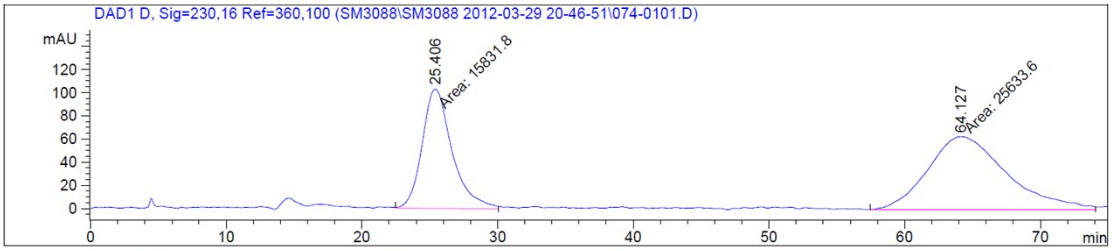

Signal 3: DAD1 D, Sig=230,16 Ref=360,100

Peak #	RetTime [min]	Type	Width [min]	Area [mAU*s]	Height [mAU]	Area %
1	25.406	MM	2.5605	1.58318e4	103.05199	38.1807
2	64.127	MM	6.7607	2.56336e4	63.19254	61.8193
Totals :				4.14654e4	166.24453	

Figure S-31 HPLC trace of (*S*)-10d

(*R*)-diethyl 2-(1-(4-bromophenyl)-2-nitroethyl)malonate ((*R*)-10d):

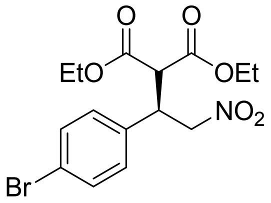

Λ-1 used as catalyst. Analytical data for title compound: (63 mg, 48%), $[\alpha]_D^{25}$ = -6.5 (*c* 0.82, $CHCl_3$); HPLC (Chiralcel AD-H, hexane/isopropanol= 90/10, 0.8 mL/min, λ= 210 nm, retention times : (R) (Major) 22.8 min, (S) (Minor) 55.7 min, 72% ee).

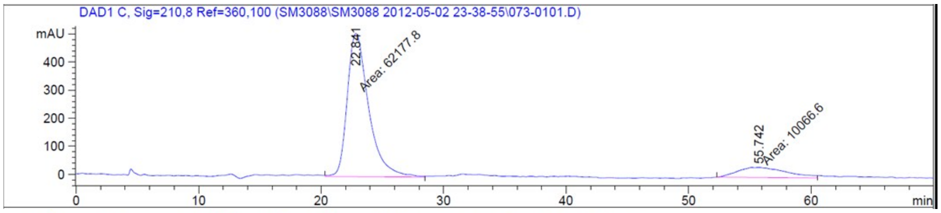

Signal 2: DAD1 C, Sig=210,8 Ref=360,100

Peak #	RetTime [min]	Type	Width [min]	Area [mAU*s]	Height [mAU]	Area %
1	22.841	MM	2.0304	6.21778e4	510.38382	86.0659
2	55.742	MM	4.6695	1.00666e4	35.93021	13.9341

Figure S-32 HPLC trace of (*R*)-10d

(*S*)-diethyl 2-(1-(4-hydroxyphenyl)-2-nitroethyl)malonate ((*S*)-10e):

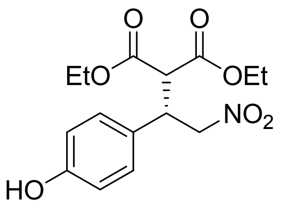

Δ-1 used as catalyst. Analytical data for title compound: (60 mg, 51%), $[\alpha]_D^{25}$ = +5.1 (*c* 0.86, $CHCl_3$); ^{1}H NMR (400 MHz, $CDCl_3$) δ 7.01 (d, *J* = 8.4 Hz, 2H), 6.64 (d, *J* = 8.4 Hz, 2H), 4.95 (bs, 1H), 4.81 (dd, *J* = 12.8, 4.8 Hz, 1H), 4.78 (dd, *J* = 17.2, 4.8 Hz, 1H), 4.16 - 4.09 (m, 3H), 3.95 (q, *J* = 7.2Hz, 2H), 3.69 (d, *J* = 9.6 Hz, 1H). 1.19 (t, *J* = 6.8 Hz, 3H), 0.99 (t, *J* = 7.2 Hz, 3H); ^{13}C NMR (100 MHz, $CDCl_3$) δ 167.5, 166.4, 129.4, 128.1, 115.8, 77.9, 62.2, 61.9, 55.1, 42.3, 13.9, 13.8. HRMS (EI) calc. for $[C_{17}H_{24}NO_8]^+$ 348.1059, found 348.1087; HPLC (Chiralcel AD-H, hexane/isopropanol= 90/10, 0.8 mL/min, λ= 210 nm, retention times: (R) (Minor) 26.5 min, (S) (Major) 39.9 min, 40% ee).

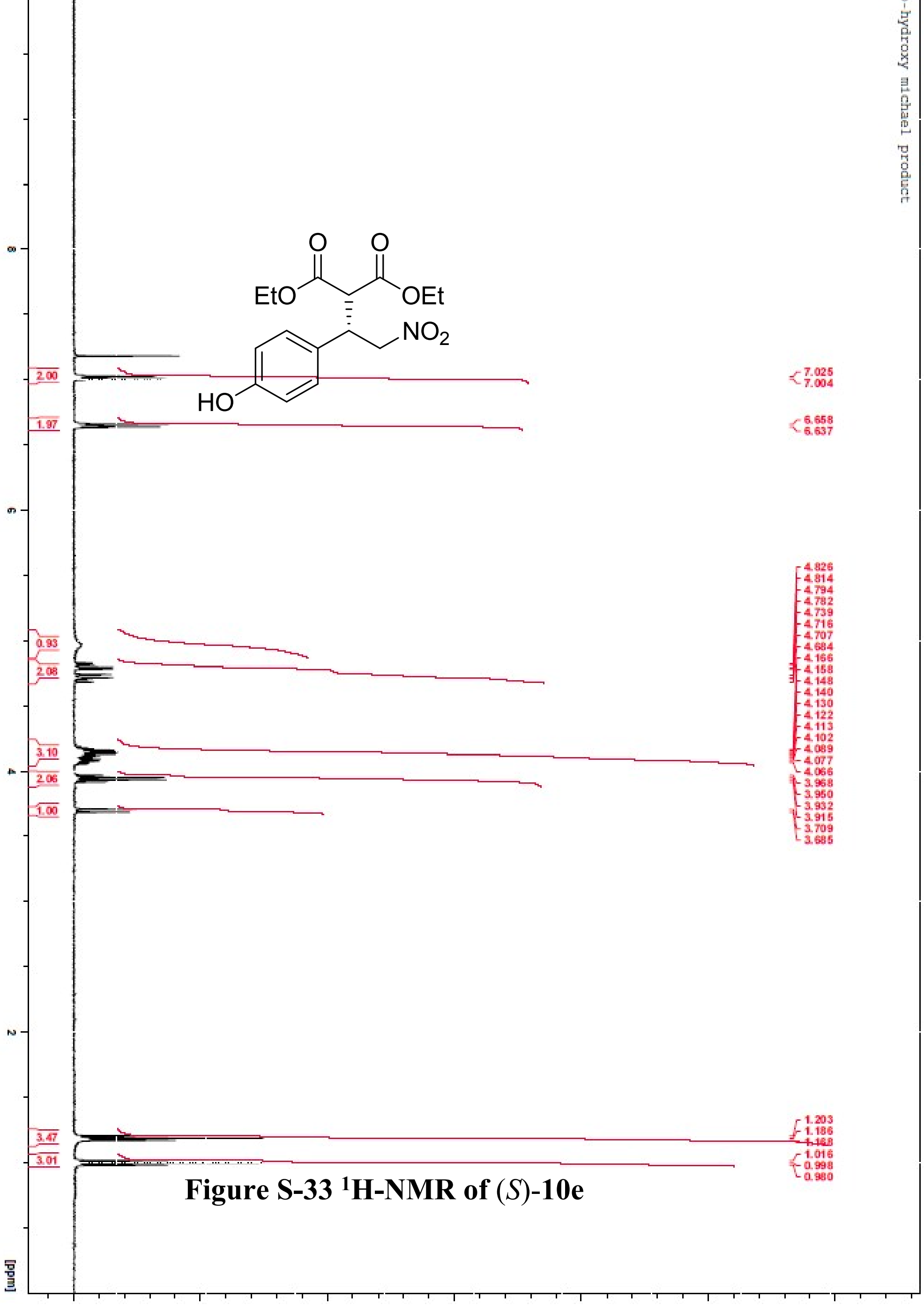

Figure S-33 ^{1}H-NMR of (*S*)-10e

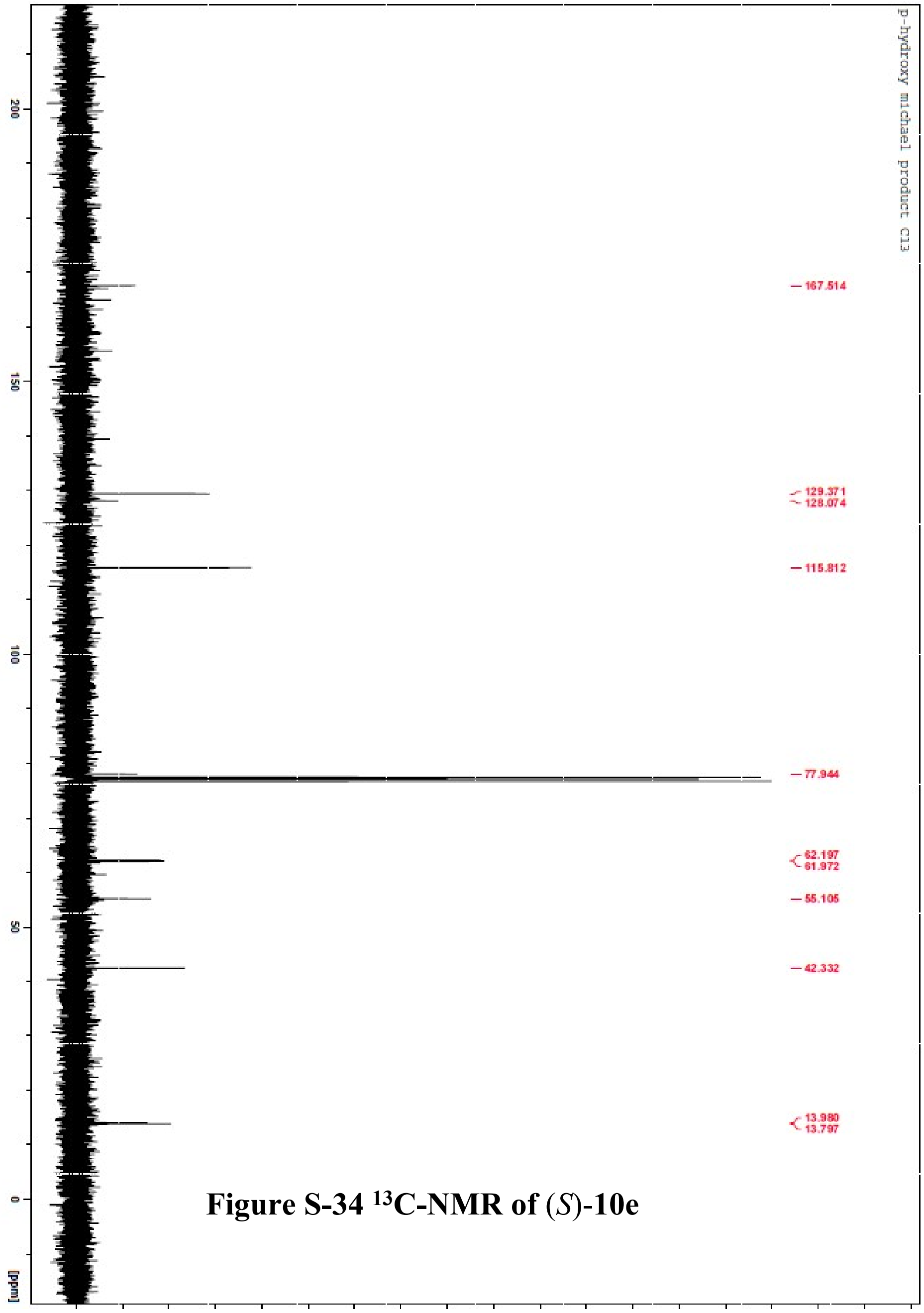

Figure S-34 ^{13}C-NMR of (*S*)-10e

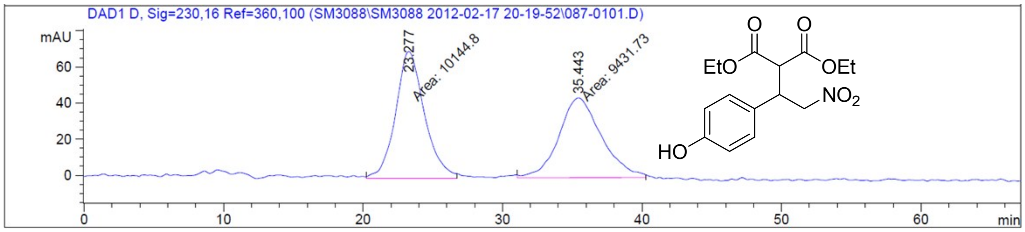

Signal 3: DAD1 D, Sig=230,16 Ref=360,100

Peak #	RetTime [min]	Type	Width [min]	Area [mAU*s]	Height [mAU]	Area %
1	23.277	MM	2.4070	1.01448e4	70.24554	51.8213
2	35.443	MM	3.5493	9431.73145	44.28881	48.1787
Totals :				1.95766e4	114.53436	

Figure S-35 HPLC trace of racemic 10e

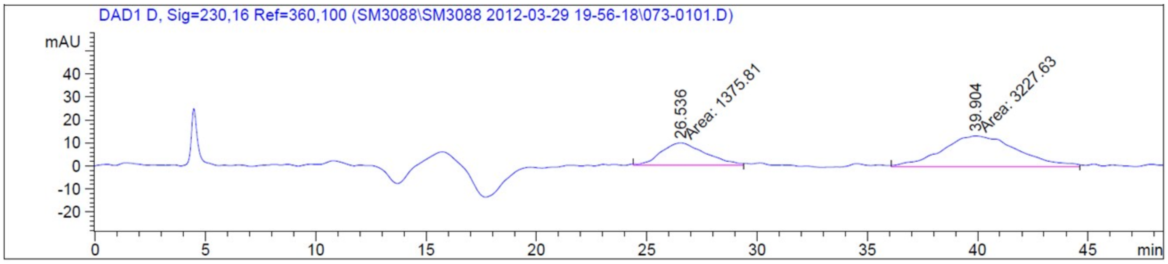

Signal 3: DAD1 D, Sig=230,16 Ref=360,100

Peak #	RetTime [min]	Type	Width [min]	Area [mAU*s]	Height [mAU]	Area %
1	26.536	MM	2.3548	1375.81091	9.73760	29.8865
2	39.904	MM	4.0069	3227.63477	13.42546	70.1135
Totals :				4603.44568	23.16306	

Figure S-36 HPLC trace of (*S*)-10e

(*R*)-diethyl 2-(1-(4-hydroxyphenyl)-2-nitroethyl)malonate ((*R*)-10e):

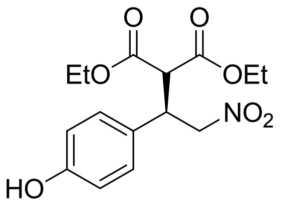

Λ-1 used as catalyst. Analytical data for title compound: (52 mg, 44%), $[\alpha]_D^{25}$ = -1.7 (*c* 0.84, $CHCl_3$); HPLC (Chiralcel AD-H, hexane/isopropanol= 90/10, 0.8 mL/min, λ= 210 nm, retention times : (R) (Major) 23.9 min, (S) (Minor) 35.2 min, 53% ee).

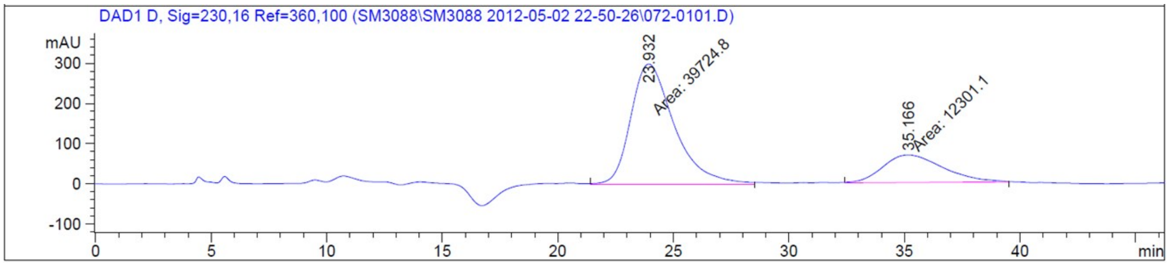

Signal 3: DAD1 D, Sig=230,16 Ref=360,100

Peak #	RetTime [min]	Type	Width [min]	Area [mAU*s]	Height [mAU]	Area %
1	23.932	MM	2.2030	3.97248e4	300.52914	76.3558
2	35.166	MM	2.9988	1.23011e4	68.36698	23.6442
Totals :				5.20259e4	368.89613	

Figure S-37 HPLC trace of (*R*)-10e

(*S*)-dimethyl 2-(2-nitro-1-phenylethyl)malonate ((*S*)-**10f**):

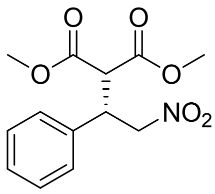

Δ-1 used as catalyst. Analytical data for title compound: (34 mg, 35%), $[\alpha]_D^{25}$ = +6.8 (*c* 0.68, $CHCl_3$); ^{1}H NMR (400 MHz, $CDCl_3$) δ 7.26-7.13 (m, 5H), 4.83 (dd, *J* = 13.2, 5.2 Hz, 1H), 4.79 (dd, *J* = 13.2, 8.8 Hz, 1H), 4.19 - 4.13 (m, 1H), 3.78 (d, *J* = 9.2Hz, 1H), 3.67 (s, 3H). 3.48 (s, 3H) ; ^{13}C NMR (100 MHz, $CDCl_3$) δ 167.8, 167.2, 136.1, 129.0, 128.4, 127.9, 77.4, 54.8, 53.0, 52.8, 42.9, 30.9. HRMS (EI) calc. for $[C_{13}H_{15}NNaO_6]^+$ 304.0797, found 304.0794; HPLC (Chiralcel OD, hexane/isopropanol= 95/5, 0.8 mL/min, λ= 210 nm, retention times: (R) (Minor) 19.3 min, (S) (Major) 27.0 min, 57% ee).

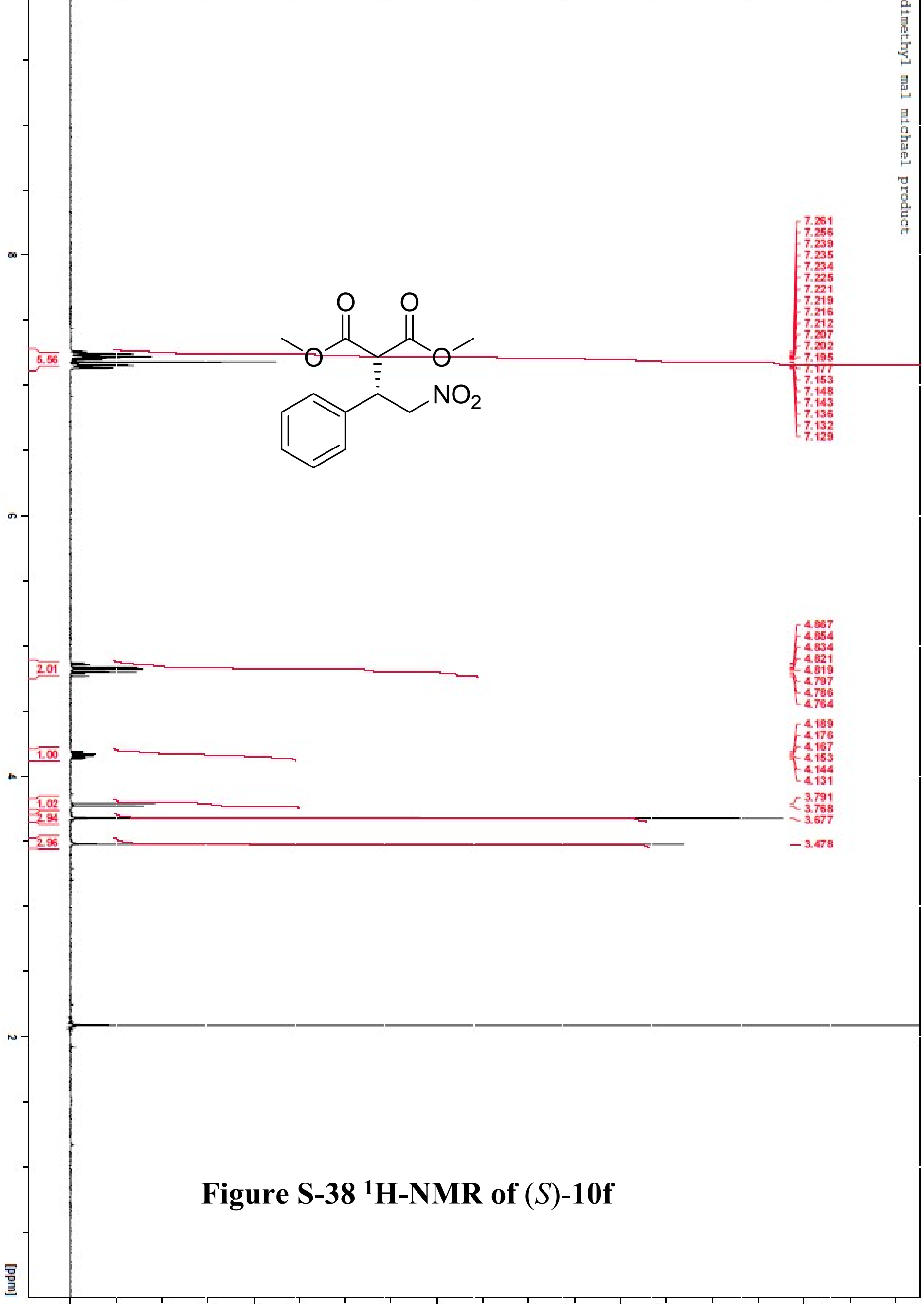

Figure S-38 ^{1}H-NMR of (*S*)-10f

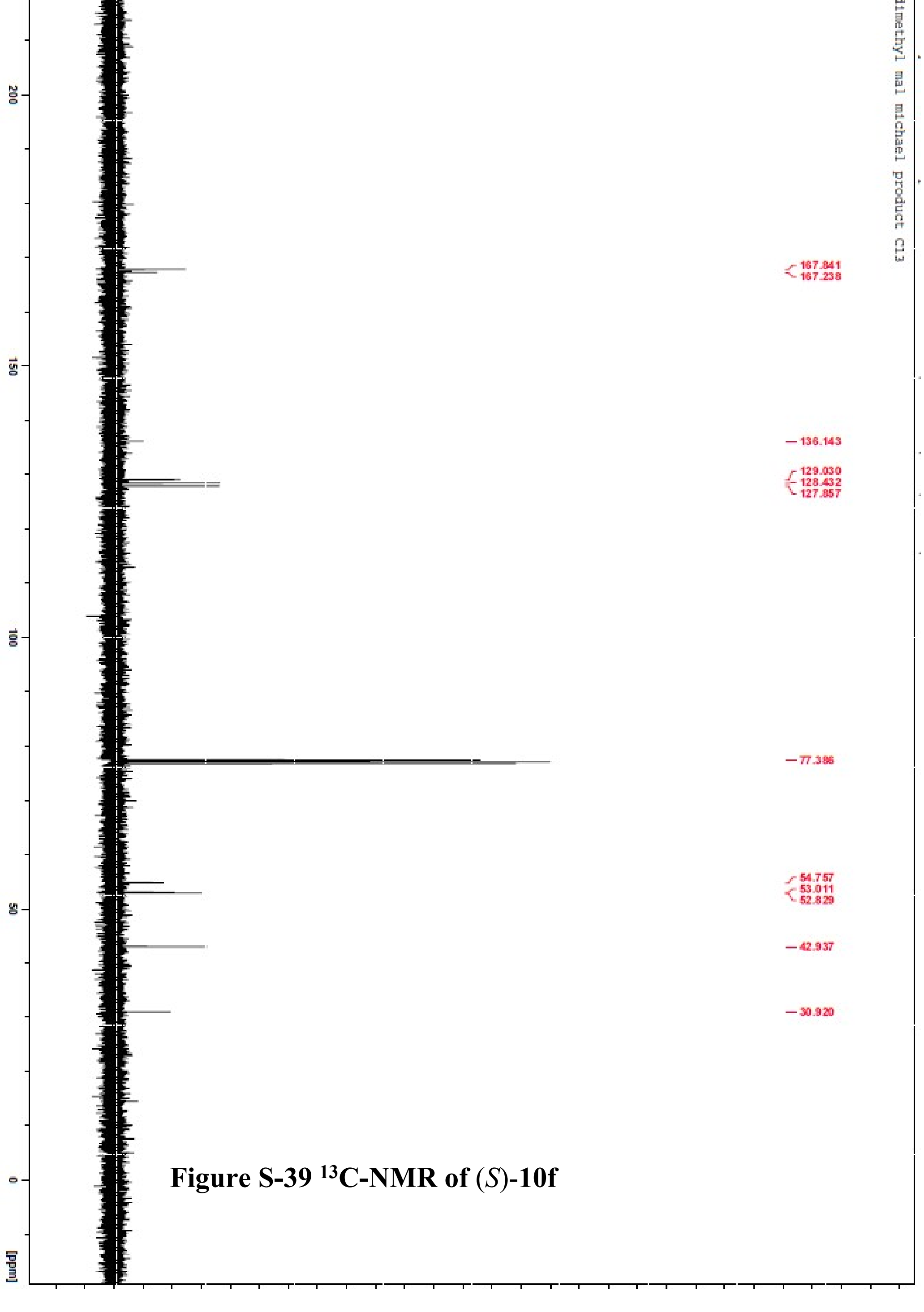

Figure S-39 ^{13}C-NMR of (*S*)-10f

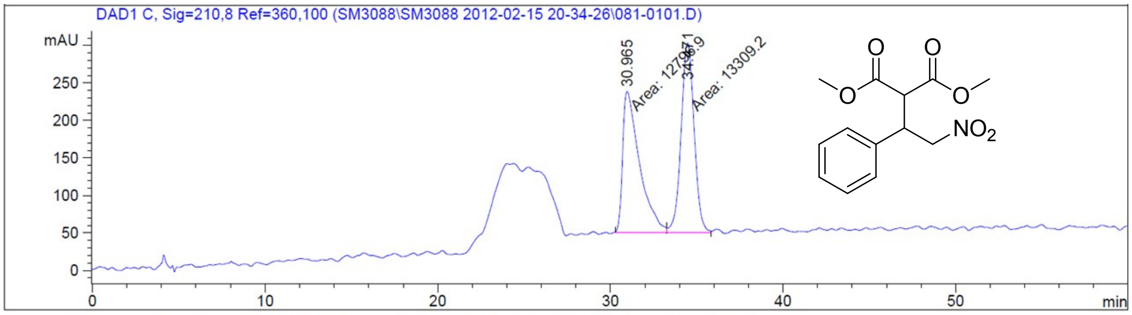

Signal 2: DAD1 C, Sig=210,8 Ref=360,100

Peak #	RetTime [min]	Type	Width [min]	Area [mAU*s]	Height [mAU]	Area %
1	30.965	MM	1.1315	1.27969e4	188.49965	49.0187
2	34.471	MM	0.8766	1.33092e4	253.03561	50.9813
Totals :				2.61061e4	441.53526	

Figure S-40 HPLC trace of racemic 10f

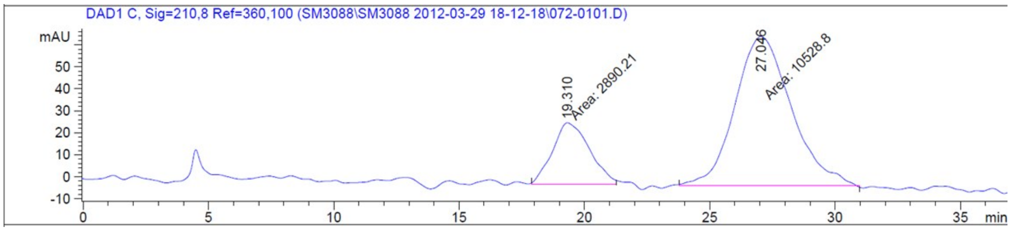

Signal 2: DAD1 C, Sig=210,8 Ref=360,100

Peak #	RetTime [min]	Type	Width [min]	Area [mAU*s]	Height [mAU]	Area %
1	19.310	MM	1.7193	2890.21338	28.01811	21.5381
2	27.046	MM	2.5972	1.05288e4	67.56586	78.4619

Figure S-41 HPLC trace of (*S*)-10f

(*R*)-dimethyl 2-(2-nitro-1-phenylethyl)malonate ((*R*)-**10f**):

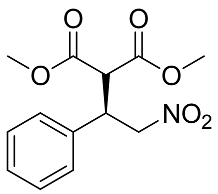

Λ-1 used as catalyst. Analytical data for title compound: (73 mg, 77%), $[\alpha]_D^{25}$ = -4.9 (*c* 0.54, $CHCl_3$); HPLC (Chiralcel OD, hexane/isopropanol= 95/5, 0.8 mL/min, λ= 210 nm, retention times: (R) (Major) 18.1 min, (S) (Minor) 24.2 min, 64% ee).

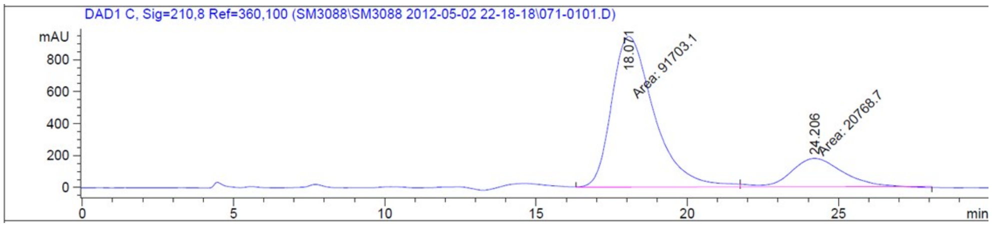

Signal 2: DAD1 C, Sig=210,8 Ref=360,100

Peak #	RetTime [min]	Type	Width [min]	Area [mAU*s]	Height [mAU]	Area %
1	18.071	MF	1.6242	9.17031e4	941.01575	81.5343
2	24.206	FM	1.9622	2.07687e4	176.40268	18.4657

Figure S-42 HPLC trace of (*R*)-10f

(*S*)-diisopropyl 2-(2-nitro-1-phenylethyl)malonate ((*S*)-10g):

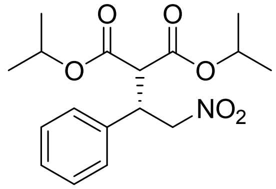

Δ-1 used as catalyst. Analytical data for title compound: (34 mg, 30%), $[\alpha]_D^{25}$ = +6.8 (*c* 0.68, $CHCl_3$); ^{1}H NMR (400 MHz, $CDCl_3$) δ 7.25-7.14 (m, 5H), 5.02 (m, 1H), 4.86-4.72 (m, 3H), 4.15 - 4.09 (m, 1H), 3.67 (d, *J* = 9.6 Hz, 1H), 1.16 (d, *J* = 6.3 Hz, 3H), 1.15 (d, *J* = 6.3 Hz, 3H), 0.97 (d, *J* = 6.3 Hz, 3H), 0.94 (d, *J* = 6.3 Hz, 3H); ^{13}C NMR (100 MHz, $CDCl_3$) δ 167.1, 166.3, 136.3, 128.9, 128.3, 128.1, 77.9, 69.9, 69.6, 55.2, 42.9, 21.6, 21.5, 21.3, 21.2. HRMS (EI) calc. for $[C_{13}H_{15}NNaO_6]^+$ 360.1423, found 360.1432; HPLC (Chiralcel OD, hexane/isopropanol= 95/5, 0.8 mL/min, λ= 210 nm, retention times: (R) (Minor) 9.8 min, (S) (Major) 11.1 min, 73% ee).

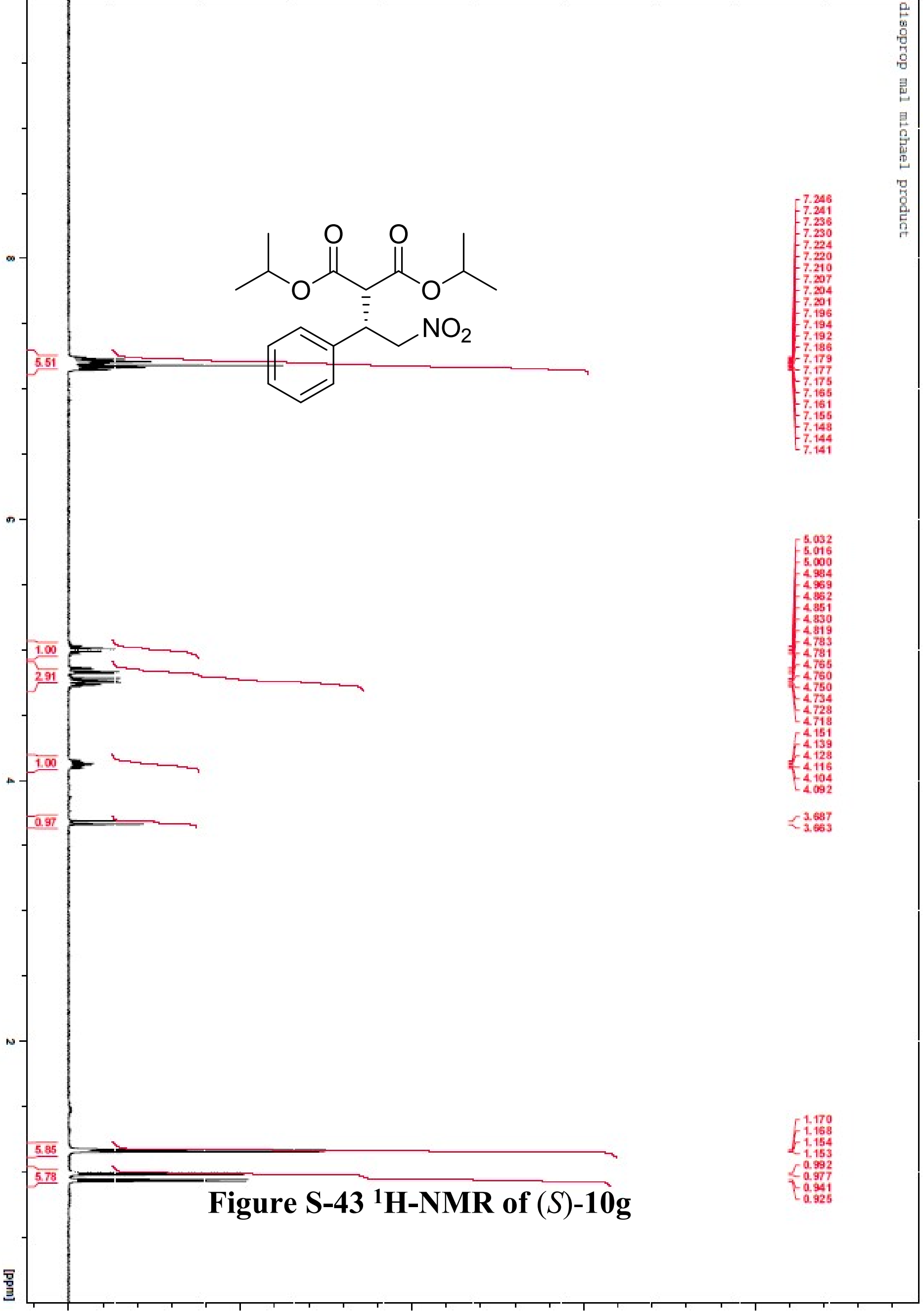

Figure S-43 ^{1}H-NMR of (*S*)-10g

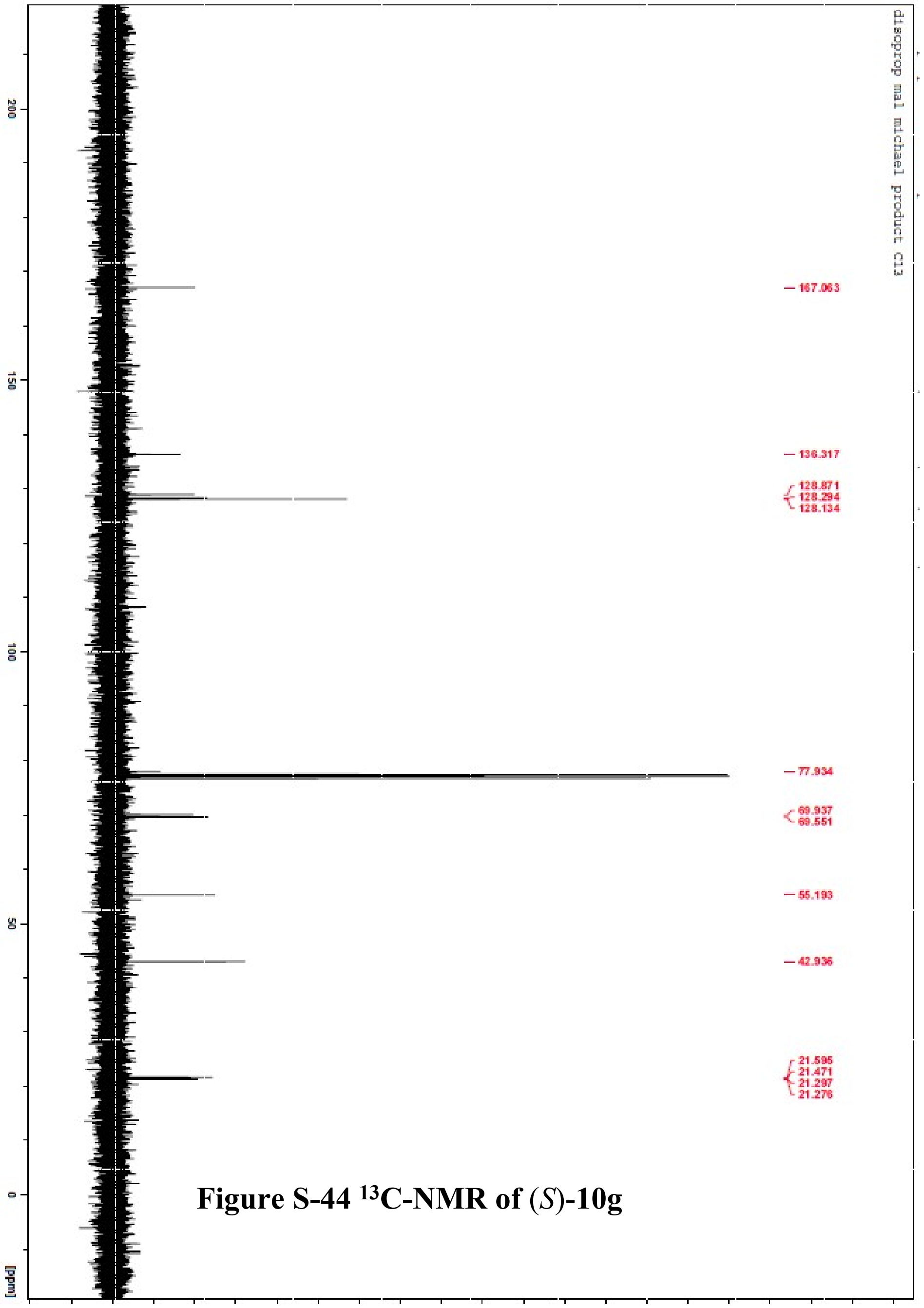

Figure S-44 ^{13}C-NMR of (*S*)-10g

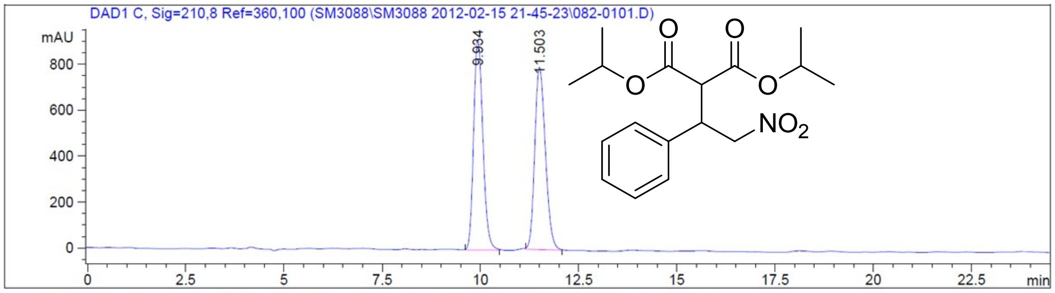

Signal 2: DAD1 C, Sig=210,8 Ref=360,100

Peak #	RetTime [min]	Type	Width [min]	Area [mAU*s]	Height [mAU]	Area %
1	9.934	BB	0.2568	1.49436e4	913.58557	50.0395
2	11.503	BB	0.2934	1.49200e4	793.34052	49.9605
Totals :				2.98637e4	1706.92609	

Figure S-45 HPLC trace of racemic 10g

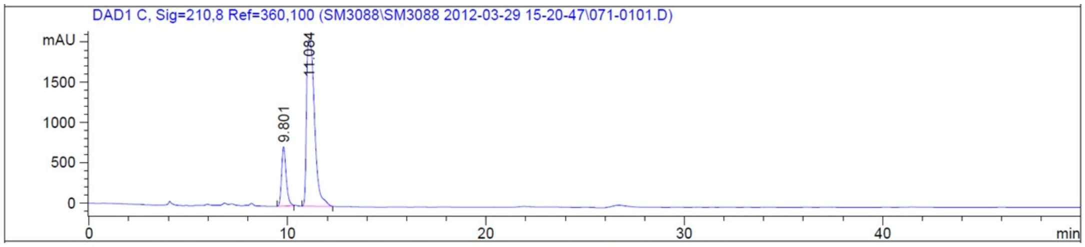

Signal 2: DAD1 C, Sig=210,8 Ref=360,100

Peak #	RetTime [min]	Type	Width [min]	Area [mAU*s]	Height [mAU]	Area %
1	9.801	BB	0.2435	1.18447e4	736.83130	17.0142
2	11.084	BB	0.4416	5.77717e4	2070.19482	82.9858
Totals :				6.96164e4	2807.02612	

Figure S-46 HPLC trace of (*S*)-10g

(*R*)-diisopropyl 2-(2-nitro-1-phenylethyl)malonate ((*R*)-10g):

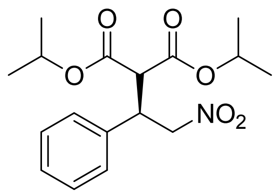

Λ-1 used as catalyst. Analytical data for title compound: (34 mg, 30%), $[\alpha]_D^{25}$ = -3.8 (*c* 0.68, $CHCl_3$); HPLC (Chiralcel OD, hexane/isopropanol= 95/5, 0.8 mL/min, λ= 210 nm, retention times: (R) (Major) 9.5 min, (S) (Minor) 10.8 min, 65% ee).

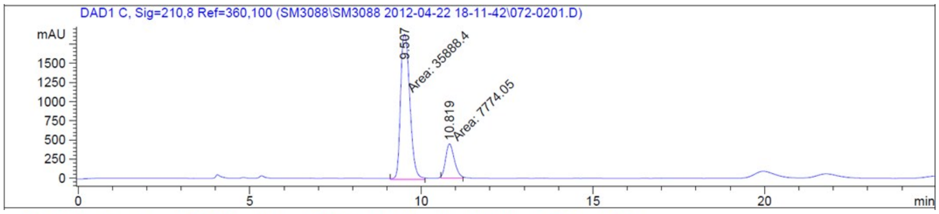

Signal 2: DAD1 C, Sig=210,8 Ref=360,100

Peak #	RetTime [min]	Type	Width [min]	Area [mAU*s]	Height [mAU]	Area %
1	9.507	MM	0.3145	3.58884e4	1901.91711	82.1951
2	10.819	MM	0.2926	7774.04590	442.73987	17.8049

Figure S-47 HPLC trace of (*R*)-10g

1-benzyl 3-ethyl 2-((*S*)-2-nitro-1-phenylethyl)malonate ((*S*)-**10j**):

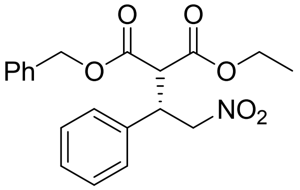

Δ-1 used as catalyst and (*S*)-**10j** was obtained as a mixture of inseparable diastereomers.

Analytical data for title compound: (26 mg, 41%, dr~50:50) , $[\alpha]_D^{25}$ = +0.73 (*c* 0.52, $CHCl_3$); ^1H NMR (400 MHz, $CDCl_3$) δ 7.29-7.04 (m, 25H), 5.10 (dd, *J* = 7.3, 5.2 Hz, 2.5H), 4.81 (s, 1.7H), 4.79 - 4.75 (m, 3.6H), 4.17-4.09 (m, 4.2H), 3.89 (q, *J* = 7.2 Hz, 2H), 3.80 (dd, *J* = 9.6, 7.2 Hz, 1.8H), 1.12 (t, *J* = 7.2 Hz, 3H), 0.92 (d, *J* = 7.2 Hz, 2.7H), ^{13}C NMR (100 MHz, $CDCl_3$) δ 167.3, 166.1, 134.9, 129.0, 128.3, 128.9, 128.6, 128.5, 128.4, 128.37, 128.30, 127.9, 77.6, 77.4, 67.7, 67.5, 62.2, 61.9, 55.0, 54.9, 43.0, 42.9, 13.8, 13.6. HRMS (EI) calc. for $[C_{20}H_{21}NNaO_6]^+$ 394.1267, found 394.1279; HPLC (Chiralcel OD-H, hexane/isopropanol= 99/1, 1.0 mL/min, λ= 210 nm, retention times: First diastereomer: major enantiomer t_r = 50.2 min, minor enantiomer t_r = 54.7 min; 26% ee; Second diastereomer: minor enantiomer t_r = 59.3 min, major enantiomer t_r = 69.8 min; 30% ee.

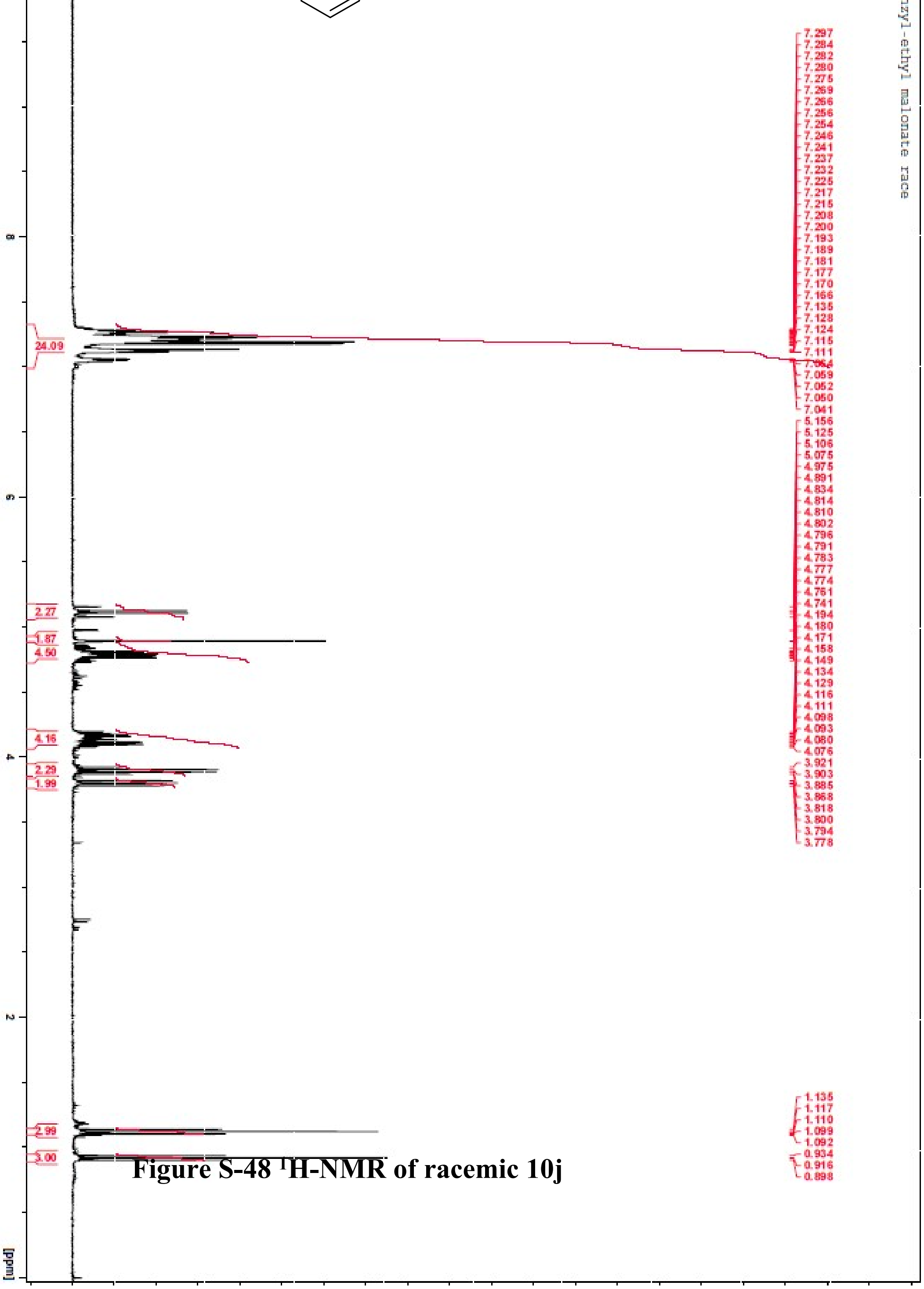

Figure S-48 ^{1}H-NMR of racemic 10j

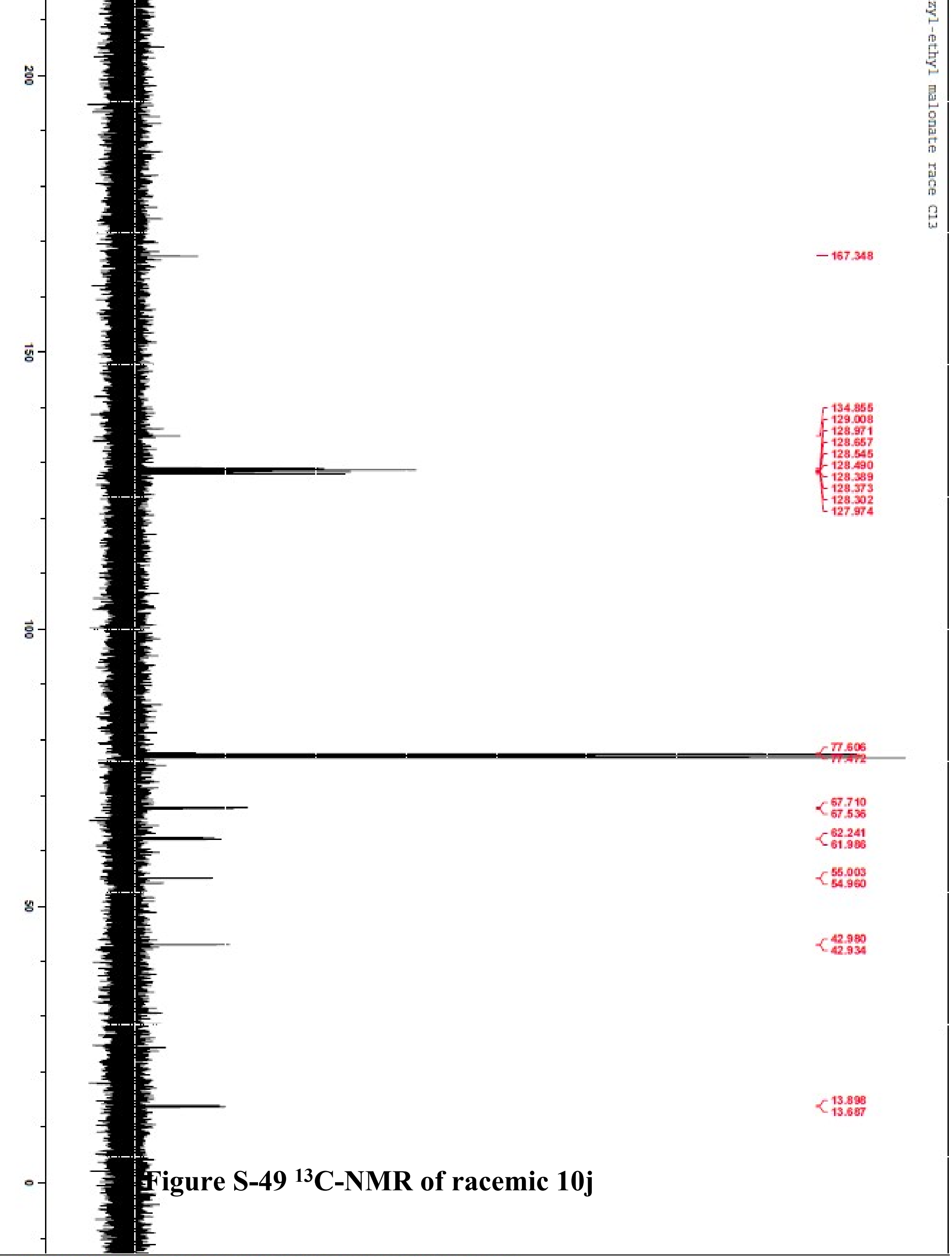

Figure S-49 ^{13}C-NMR of racemic 10j

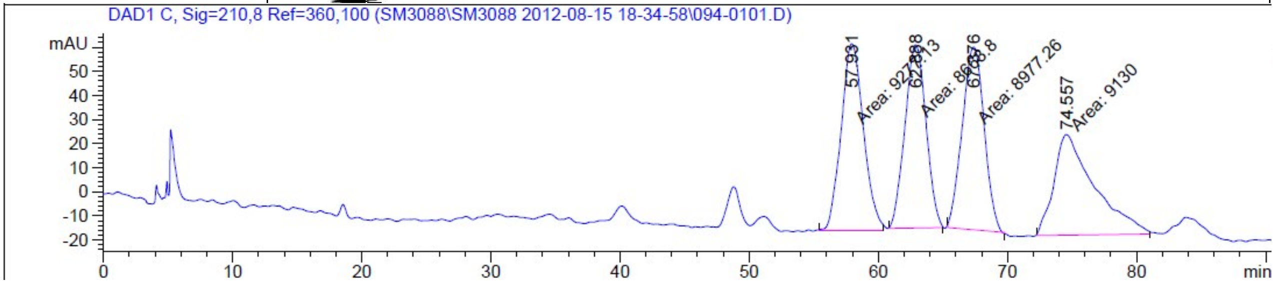

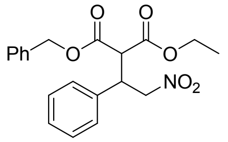

Signal 2: DAD1 C, Sig=210,8 Ref=360,100

Peak #	RetTime [min]	Type	Width [min]	Area [mAU*s]	Height [mAU]	Area %
1	57.931	MM	1.9961	9277.13281	77.46063	25.7354
2	62.888	MM	1.8950	8663.80176	76.20068	24.0339

Peak #	RetTime [min]	Type	Width [min]	Area [mAU*s]	Height [mAU]	Area %
3	67.376	MM	1.9694	8977.25879	75.97376	24.9035
4	74.557	MM	3.6380	9130.00098	41.82699	25.3272

Totals : 3.60482e4 271.46207

Figure S-50 HPLC trace of racemic 10j

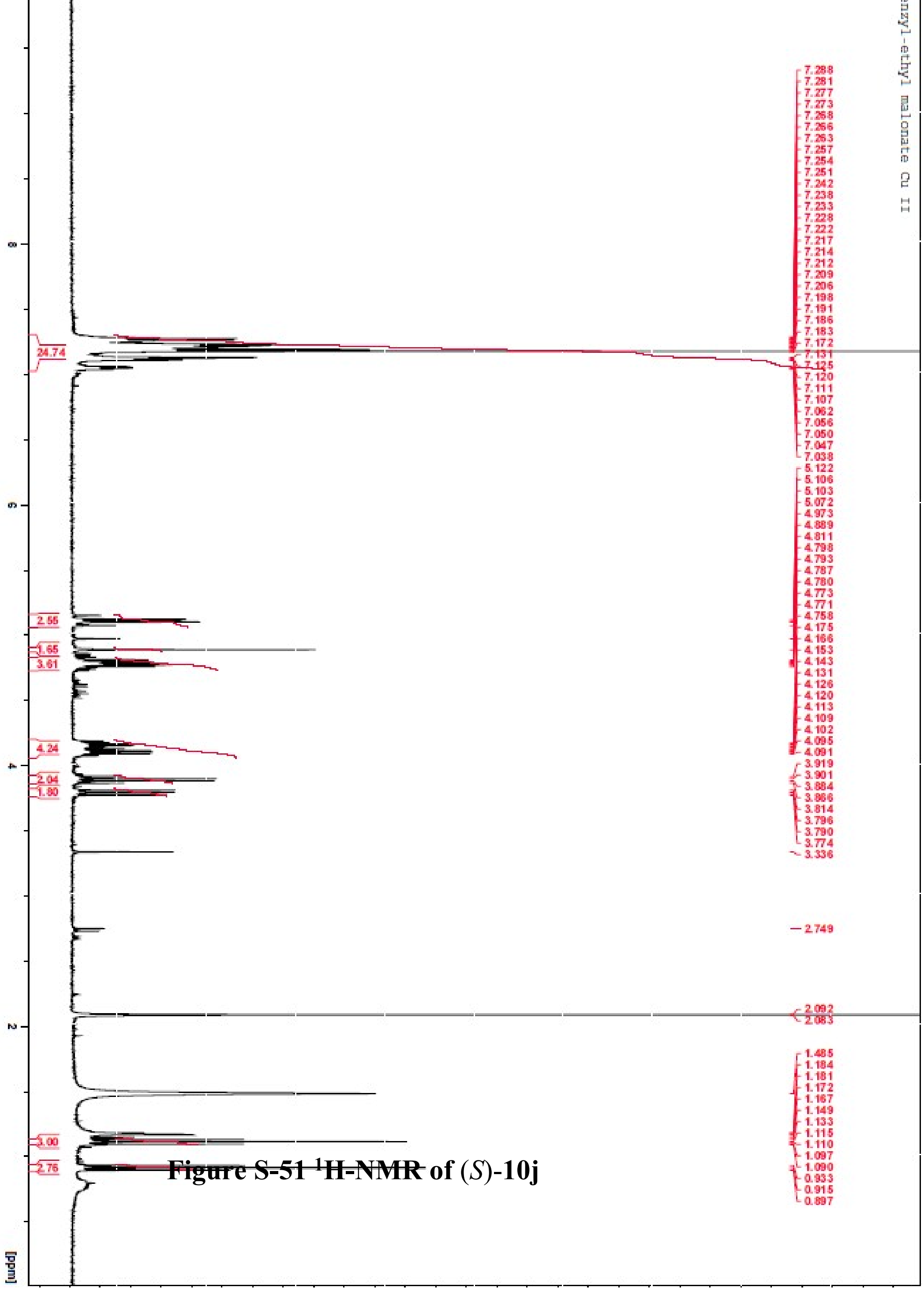

Figure S-51 ^{1}H-NMR of (*S*)-10j

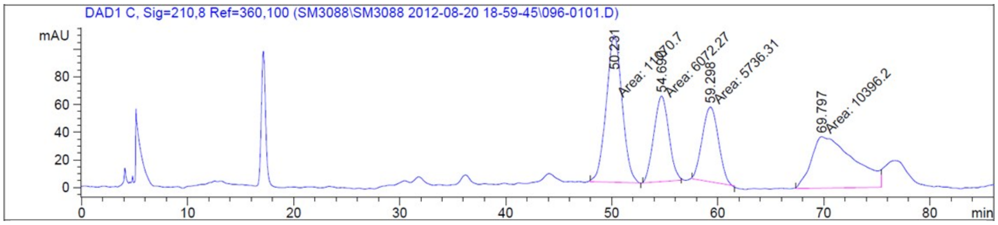

Peak #	RetTime [min]	Type	Width [min]	Area [mAU*s]	Height [mAU]	Area %
1	50.231	MM	1.7358	1.10707e4	106.30018	33.2699
2	54.690	MM	1.6339	6072.27002	61.93858	18.2485

Peak #	RetTime [min]	Type	Width [min]	Area [mAU*s]	Height [mAU]	Area %
3	59.298	MM	1.7702	5736.30664	54.00894	17.2388
4	69.797	MM	4.6542	1.03962e4	37.22889	31.2428

Totals : 3.32755e4 259.47659

Figure S-52 HPLC trace of (*S*)-10j

1-benzyl 3-ethyl 2-((*R*)-2-nitro-1-phenylethyl)malonate ((*R*)-**10j**):

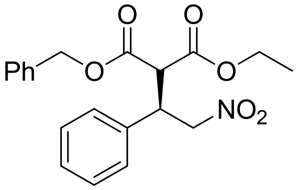

Λ-1 used as catalyst, (24 mg, 40%, dr~50:50) , $[\alpha]_D^{25}$ = -3.7 (*c* 0.50, $CHCl_3$); HPLC (Chiralcel OD-H, hexane/isopropanol= 99/1, 1.0 mL/min, λ= 210 nm, retention times: First diastereomer: minor enantiom[illegible]

major enantiom[illegible]

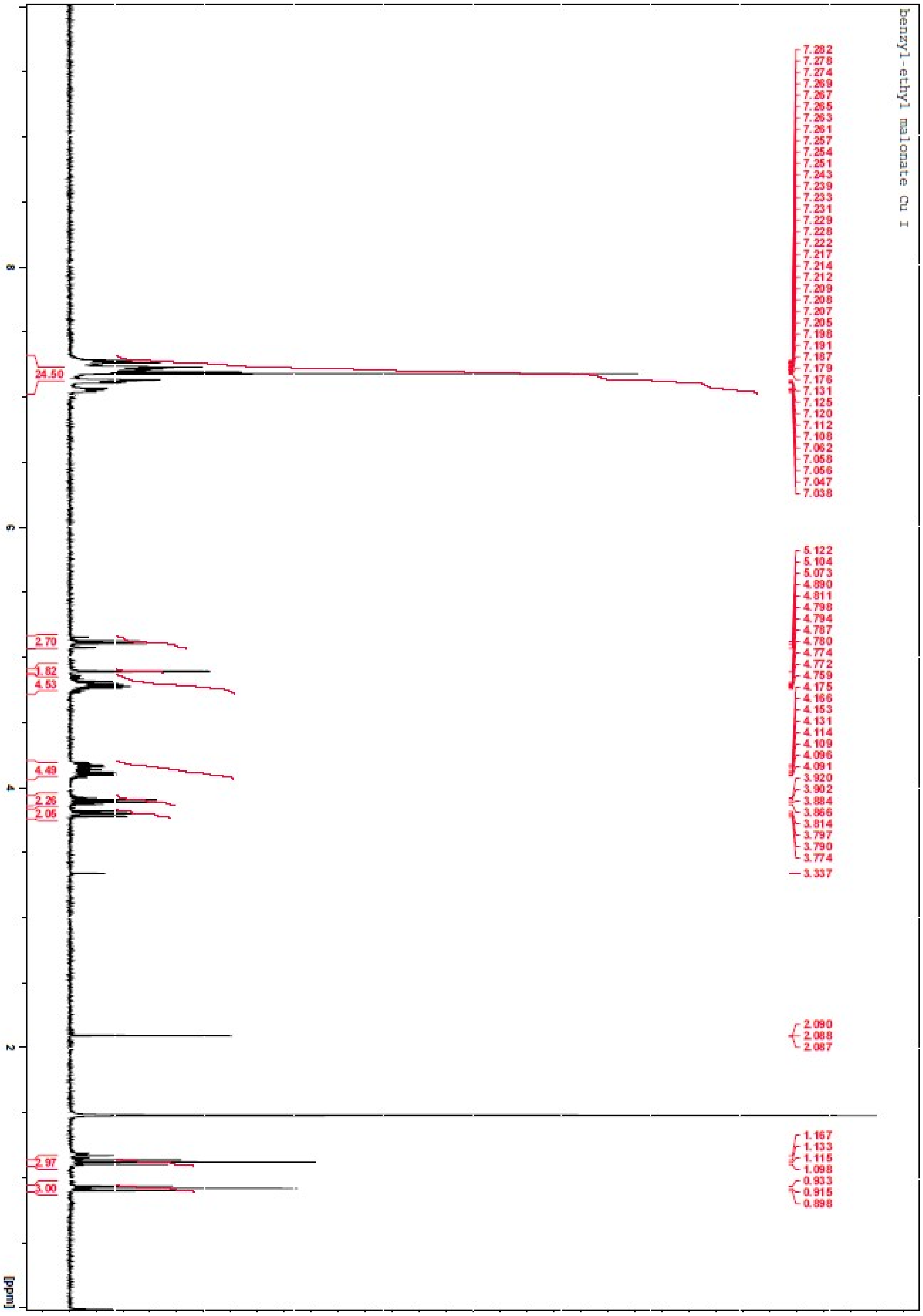

Figure S-53 ^{1}H-NMR of (*R*)-10j

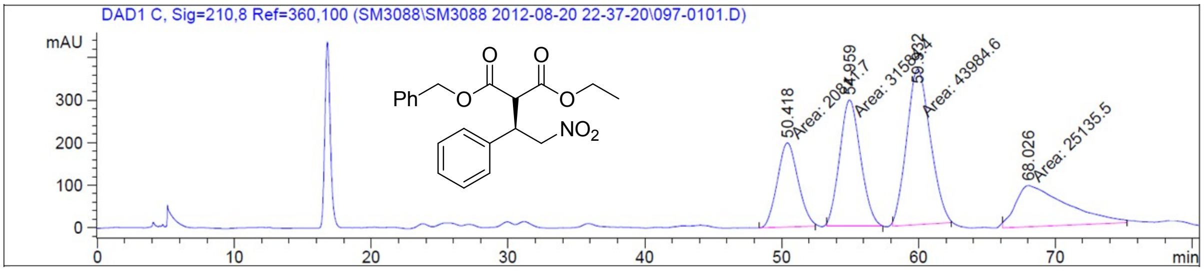

Peak #	RetTime [min]	Type	Width [min]	Area [mAU*s]	Height [mAU]	Area %
1	50.418	MM	1.7522	2.08117e4	197.95921	17.1267
2	54.959	MM	1.7730	3.15844e4	296.89551	25.9920

Peak #	RetTime [min]	Type	Width [min]	Area [mAU*s]	Height [mAU]	Area %
3	59.932	MM	2.0041	4.39846e4	365.78619	36.1965
4	68.026	MM	4.3151	2.51355e4	97.08342	20.6849
Totals :				1.21516e5	957.72433	

Figure S-54 HPLC trace of (*R*)-10j

1-benzyl 3-tert-butyl 2-((*S*)-2-nitro-1-phenylethyl)malonate ((*S*)-10l):

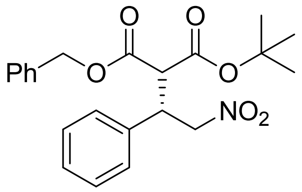

Δ-1 used as catalyst and (*S*)-**10l** was obtained as a mixture of inseparable diastereomers.

Analytical data for title compound: (34 mg, 51%, dr~67:32) , $[\alpha]_D^{25}$ = -2.2 (*c* 0.66, $CHCl_3$); ^1H NMR (400 MHz, $CDCl_3$) δ 7.28-7.07 (m, 15H), 5.08 (dd, *J* = 52.8, 12.0 Hz, 2.2H), 4.90-4.68 (m, 4.2H), 4.12-4.07 (m, 1.6H), 3.69 (d, *J* = 10.0 Hz, 1.5H), 1.29 (s, 4.2H), 1.13 (s, 9H), ^{13}C NMR (100 MHz, $CDCl_3$) δ 167.7, 167.4, 165.5, 136.2, 134.97, 134.91, 128.9, 128.8, 128.6, 128.5, 128.44, 128.43, 128.3, 128.2, 128.0, 82.9, 78.0, 67.5, 67.4, 55.8, 55.6, 43.1, 42.9, 27.7, 27.4. HRMS (EI) calc. for $[C_{22}H_{25}NNaO_6]^+$ 422.1580, found 422.1584; HPLC (Chiralcel AD-H, hexane/isopropanol= 95/5, 0.8 mL/min, λ= 210 nm, retention times: First diastereomer: minor enantiomer t_r = 35.8 min, major enantiomer t_r = 65.6 min; 37% ee; Second diastereomer: minor enantiomer t_r = 53.8 min, major enantiomer t_r = 74.4 min; 70% ee.

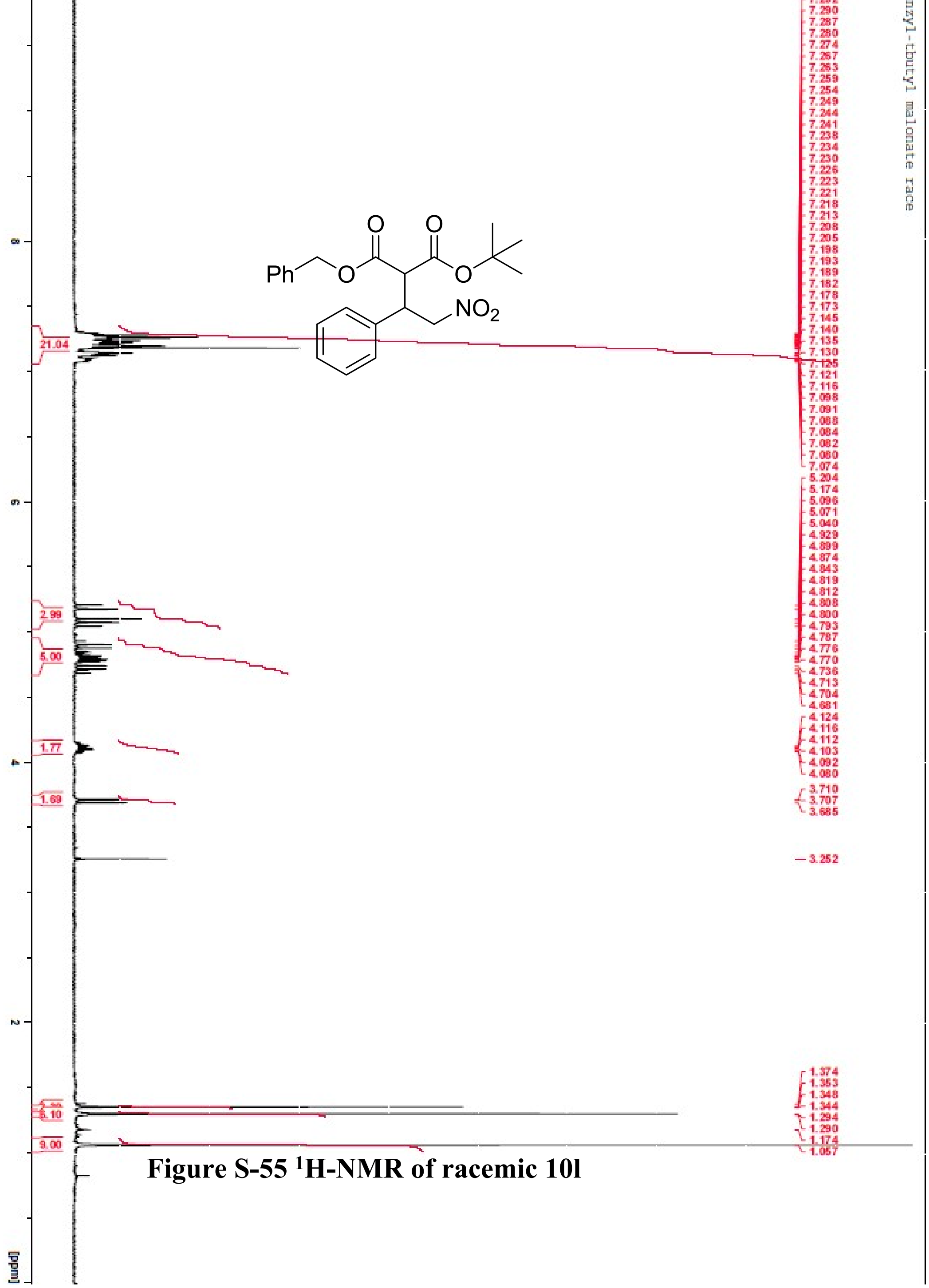

Figure S-55 ^{1}H-NMR of racemic 10l

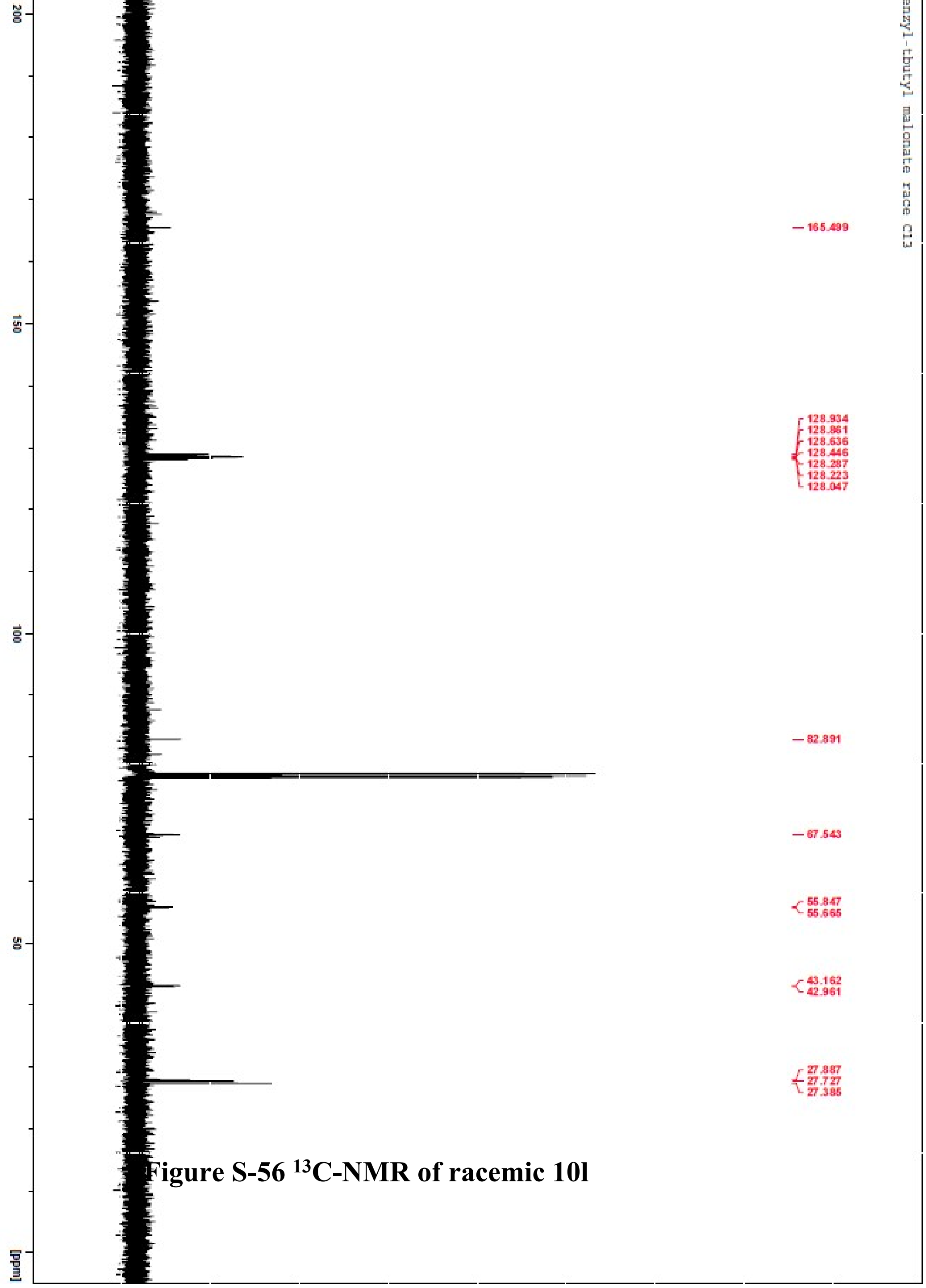

Figure S-56 ^{13}C-NMR of racemic 10l

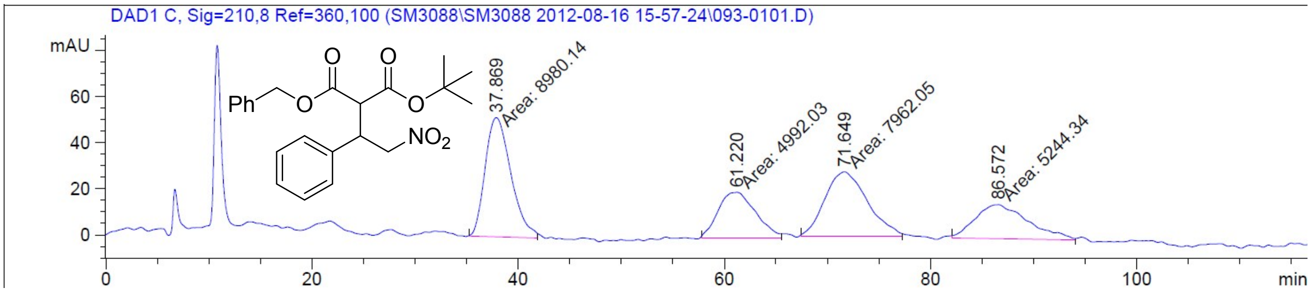

Peak #	RetTime [min]	Type	Width [min]	Area [mAU*s]	Height [mAU]	Area %
1	37.869	MM	2.8947	8980.13574	51.70438	33.0412
2	61.220	MM	4.2140	4992.03467	19.74389	18.3675

Peak #	RetTime [min]	Type	Width [min]	Area [mAU*s]	Height [mAU]	Area %
3	71.649	MM	4.7573	7962.05420	27.89420	29.2953
4	86.572	MM	5.9496	5244.33936	14.69098	19.2959

Totals : 2.71786e4 114.03345

Figure S-57 HPLC trace of racemic 10l

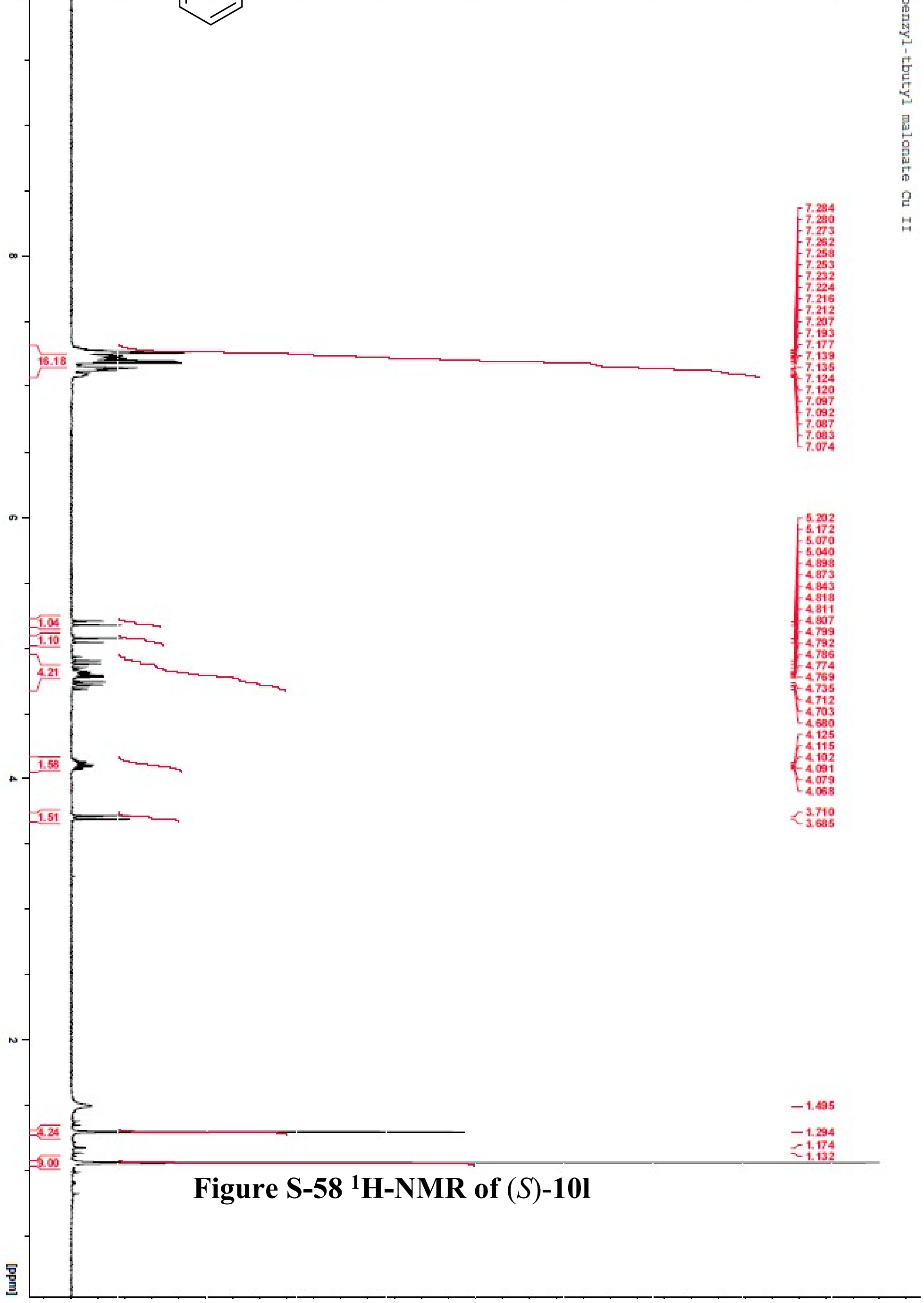

Figure S-58 ^{1}H-NMR of (*S*)-10l

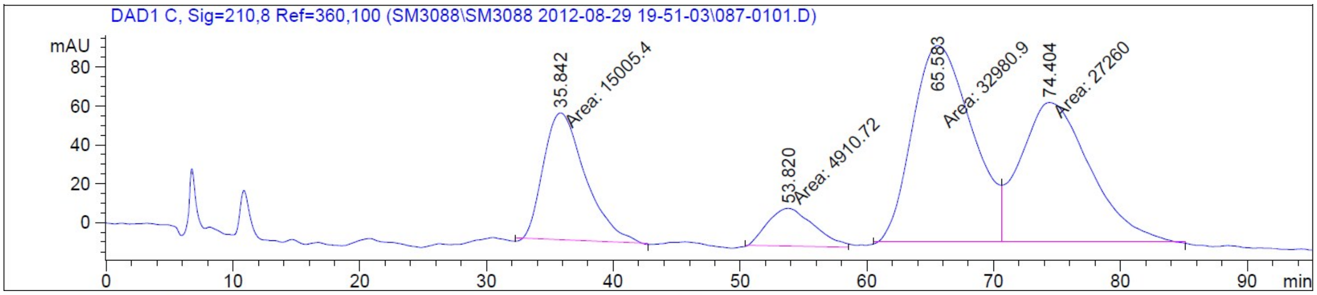

Signal 2: DAD1 C, Sig=210,8 Ref=360,100

Peak #	RetTime [min]	Type	Width [min]	Area [mAU*s]	Height [mAU]	Area %
1	35.842	MM	3.8339	1.50054e4	65.23075	18.7200
2	53.820	MM	4.2226	4910.72363	19.38260	6.1264

Peak #	RetTime [min]	Type	Width [min]	Area [mAU*s]	Height [mAU]	Area %
3	65.583	MF	5.4531	3.29809e4	100.80265	41.1454
4	74.404	FM	6.3558	2.72600e4	71.48313	34.0083

Totals :	8.01571e4	256.89913

Figure S-59 HPLC trace of (*S*)-10l

1-benzyl 3-tert-butyl 2-((*R*)-2-nitro-1-phenylethyl)malonate ((*R*)-10l):

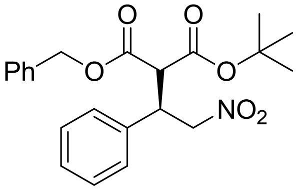

Λ-1 used as catalyst and (*R*)-**10l** was obtained as a mixture of inseparable diastereomers.

Analytical data for title compound: (28 mg, 42%, dr~50:50) , $[\alpha]_D^{25}$ = -4.2 (*c* 0.57, $CHCl_3$);

HPLC (Chiralcel AD-H, hexane/isopropanol= 95/5, 0.8 mL/min, λ= 210 nm, retention times:

First diastereon [illegible] ee;

Second diastere [illegible])% ee

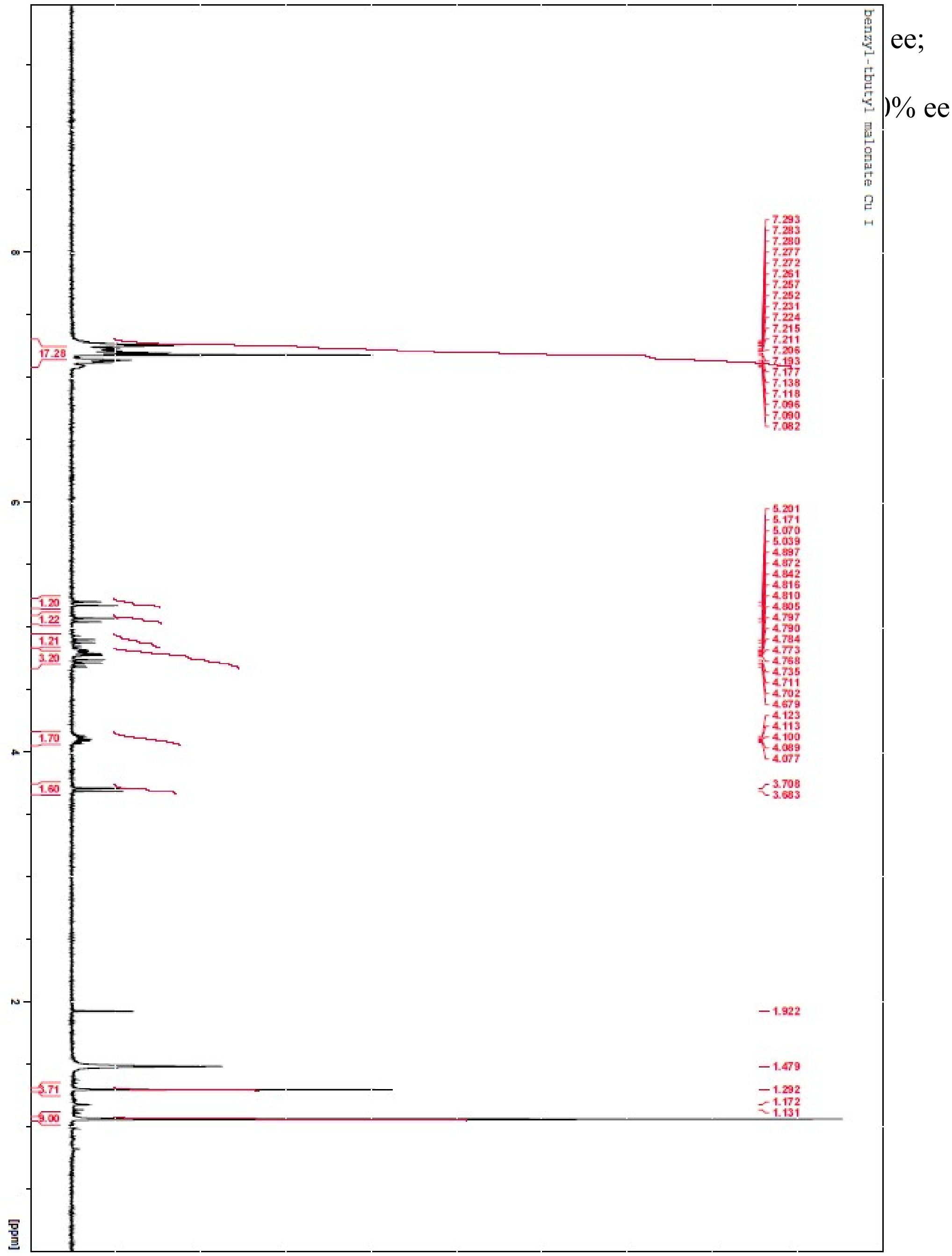

Figure S-60 ^{1}H-NMR of (*R*)-10l

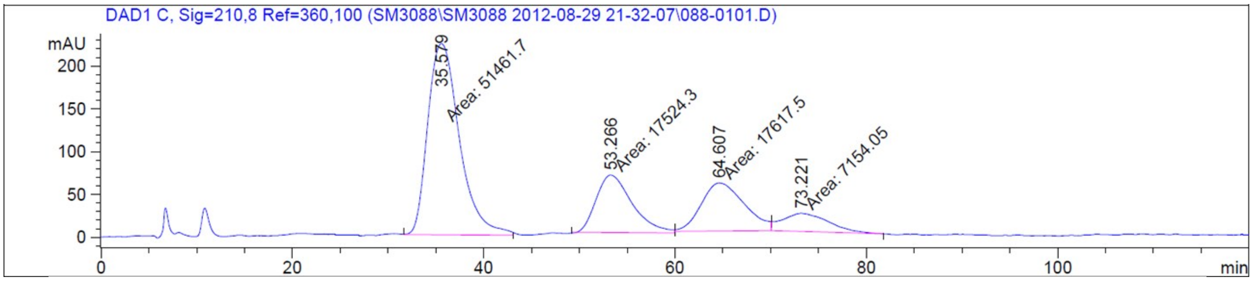

Signal 2: DAD1 C, Sig=210,8 Ref=360,100

Peak #	RetTime [min]	Type	Width [min]	Area [mAU*s]	Height [mAU]	Area %
1	35.579	MM	3.8414	5.14617e4	223.27827	54.8880
2	53.266	MM	4.3565	1.75243e4	67.04218	18.6911

Peak #	RetTime [min]	Type	Width [min]	Area [mAU*s]	Height [mAU]	Area %
3	64.607	MM	5.2308	1.76175e4	56.13378	18.7905
4	73.221	MM	5.6322	7154.05322	21.17004	7.6304

Totals : 9.37575e4 367.62428

Figure S-61 HPLC trace of (*R*)-10l

1-tert-butyl 3-ethyl 2-((*S*)-2-nitro-1-phenylethyl)malonate ((*S*)-10k):

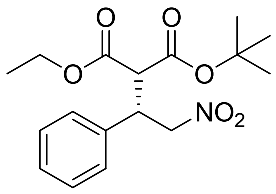

Δ-1 used as catalyst and (*S*)**-10k** was obtained as a mixture of inseparable diastereomers. Analytical data for title compound: (26 mg, 46%, dr~50:50) , $[\alpha]_D^{25}$ = -2.6 (*c* 0.54, $CHCl_3$); ^1H NMR (400 MHz, $CDCl_3$) δ 7.25-7.14 (m, 9H), 4.85-4.70 (m, 3H), 4.21-4.07 (m, 3.3H), 3.94-3.91 (m, 1.3H), 3.62 (m, 1.5H), 1.49 (s, 4.9H), 1.19 (t, *J* = 10.0 Hz, 3H), 1.13 (s, 7.4H), 0.99 (t, *J* = 10.0 Hz, 3H), ^{13}C NMR (100 MHz, $CDCl_3$) δ 166.5, 165.7, 165.5, 128.87, 128.84, 128.3, 128.2, 128.1, 78.1, 77.7, 77.2, 61.9, 61.7.0, 55.9, 55.7, 43.1, 42.9, 27.8, 27.5, 14.0, 13.8, HRMS (EI) calc. for $[C_{17}H_{23}NNaO_6]^+$ 360.1423, found 360.1434; HPLC (Chiralcel AD-H, hexane/isopropanol= 93/7, 0.8 mL/min, λ= 210 nm, retention times: First diastereomer: minor enantiomer t_r = 21.5 min, major enantiomer t_r = 33.9 min; 77% ee; Second diastereomer: minor enantiomer t_r = 27.1 min, major enantiomer t_r = 101.2 min; 72% ee

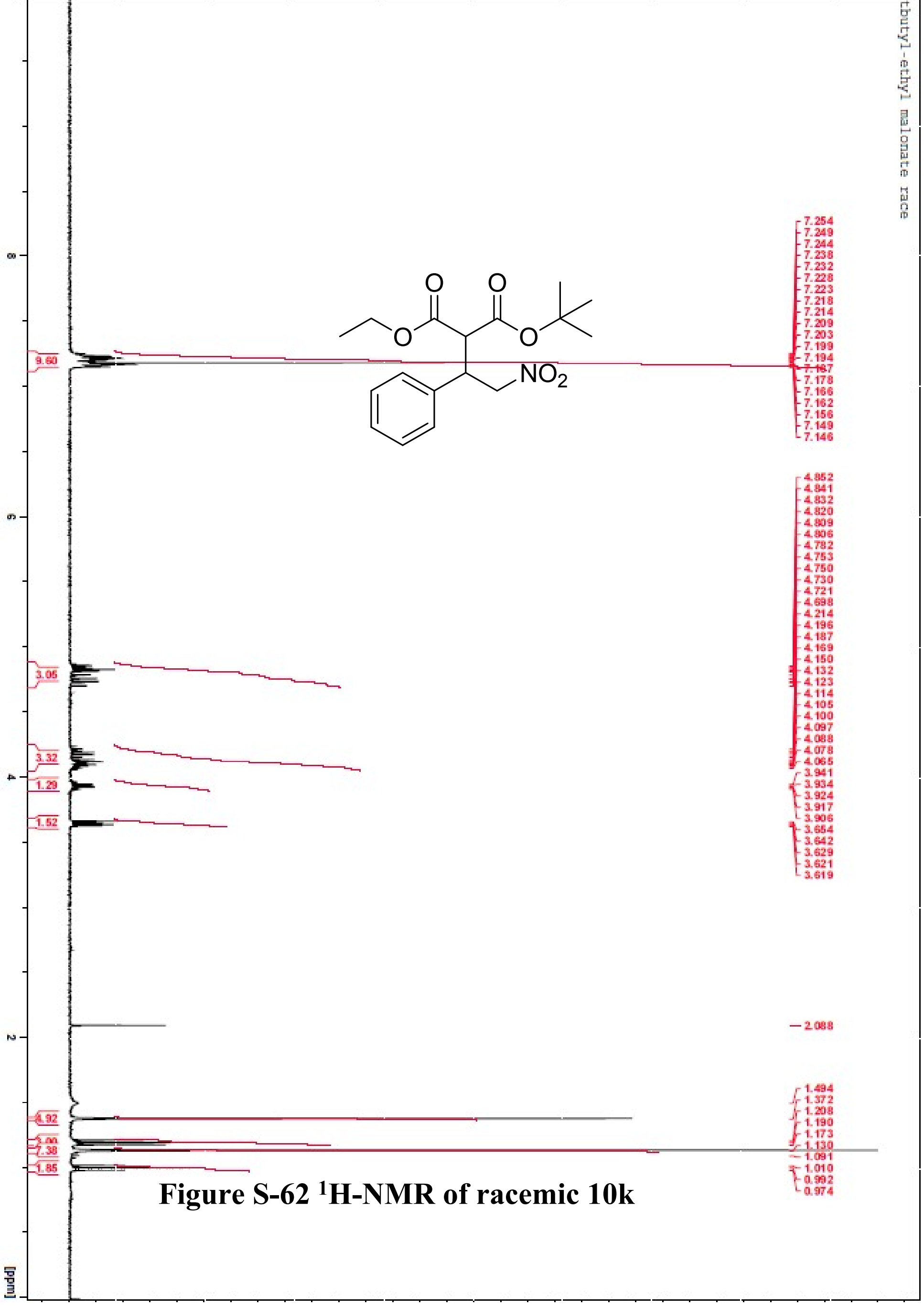

Figure S-62 ^{1}H-NMR of racemic 10k

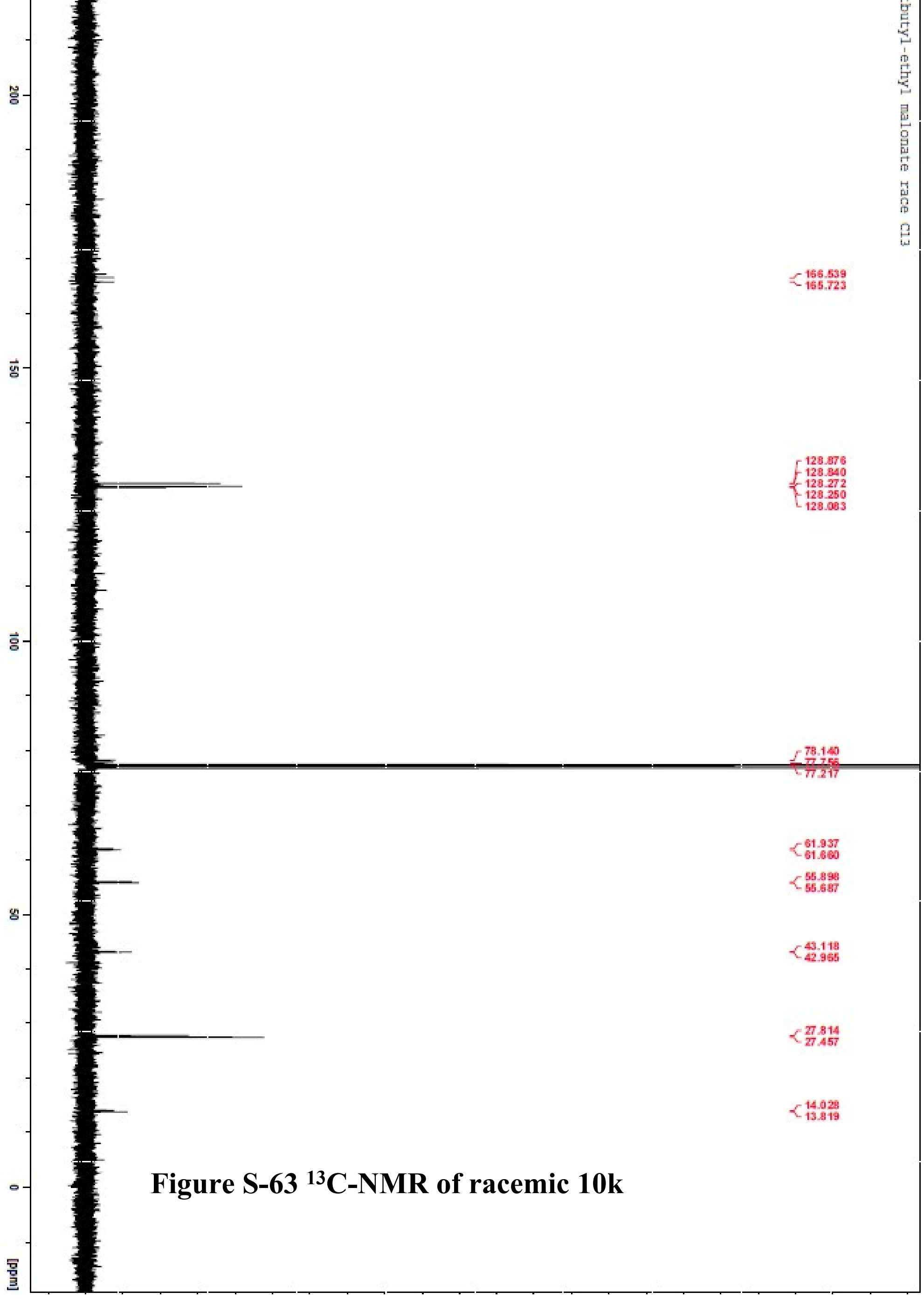

Figure S-63 ^{13}C-NMR of racemic 10k

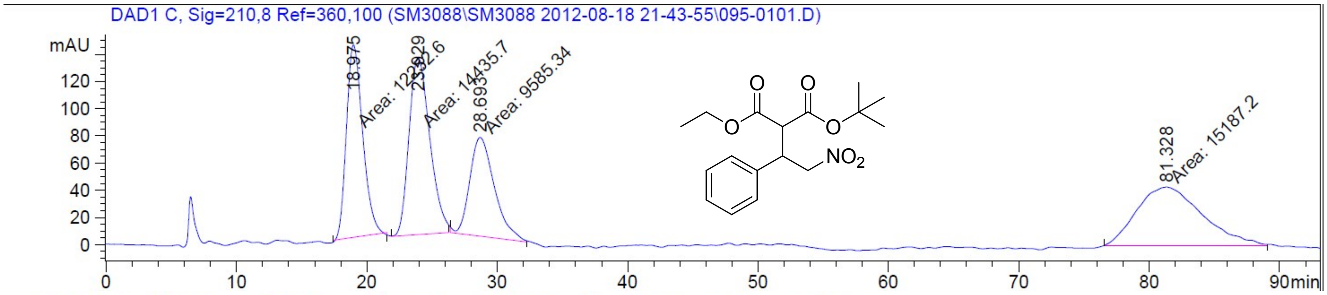

Signal 2: DAD1 C, Sig=210,8 Ref=360,100

Peak #	RetTime [min]	Type	Width [min]	Area [mAU*s]	Height [mAU]	Area %
1	18.975	MM	1.4430	1.22526e4	141.52020	23.8095
2	23.929	MM	1.8483	1.44357e4	130.16750	28.0518

Peak #	RetTime [min]	Type	Width [min]	Area [mAU*s]	Height [mAU]	Area %
3	28.693	MM	2.1973	9585.33691	72.70651	18.6265
4	81.328	MM	5.8593	1.51872e4	43.20010	29.5122

Totals : 5.14608e4 387.59431

Figure S-64 HPLC trace of racemic 10k

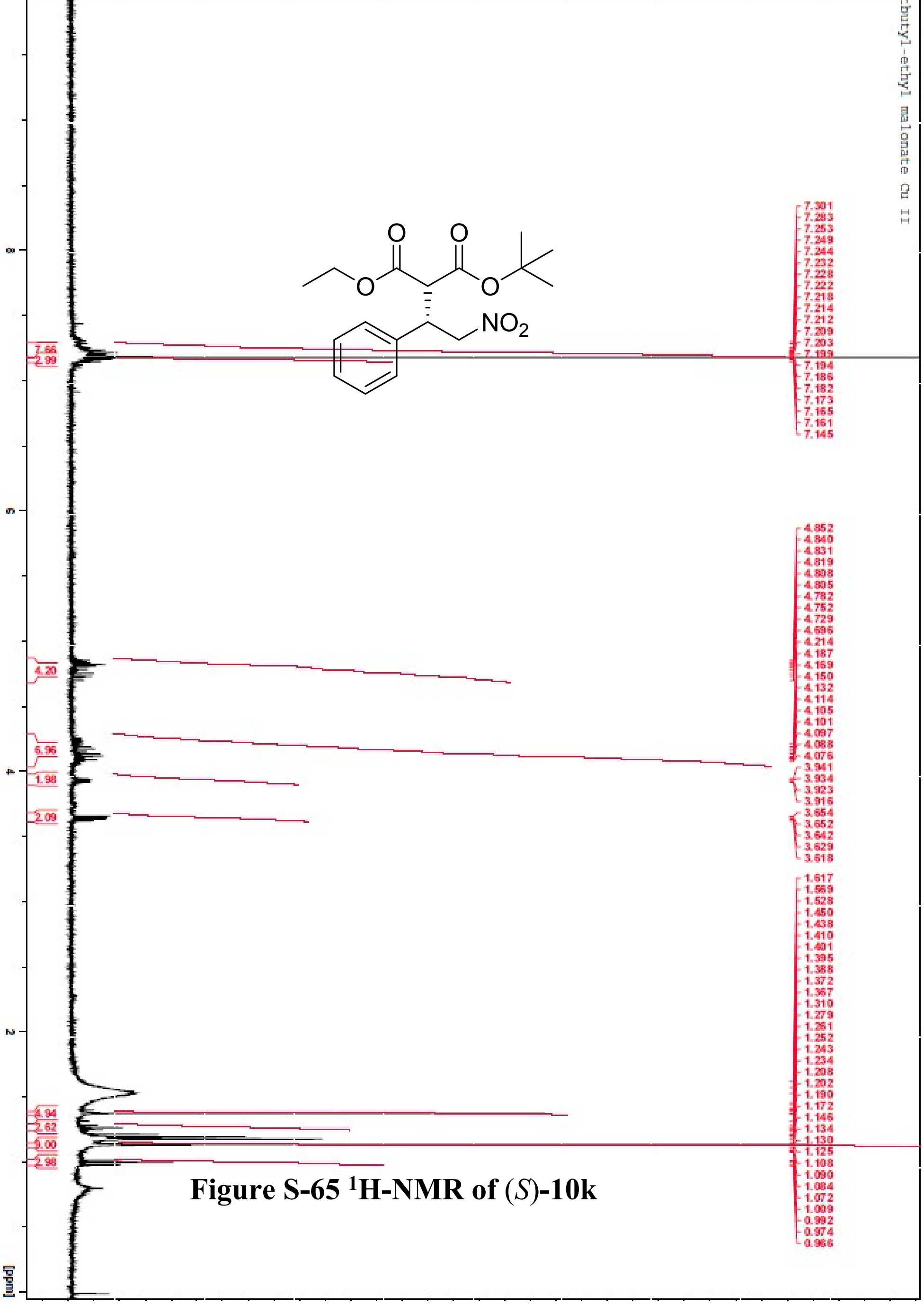

Figure S-65 ^{1}H-NMR of (*S*)-10k

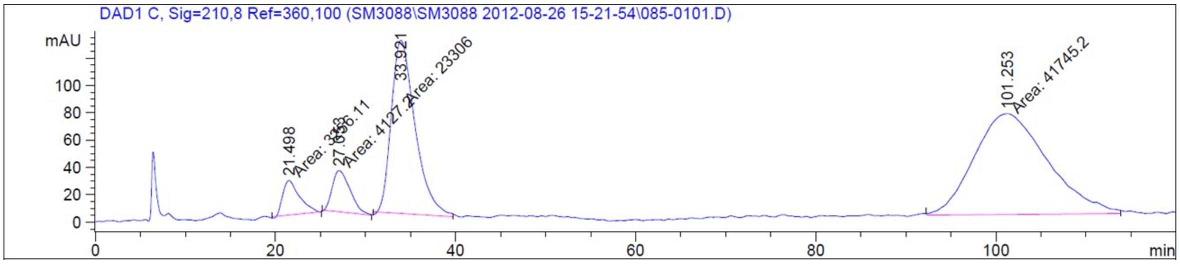

Signal 2: DAD1 C, Sig=210,8 Ref=360,100

Peak #	RetTime [min]	Type	Width [min]	Area [mAU*s]	Height [mAU]	Area %
1	21.498	MM	2.1493	3256.11475	25.24889	4.4953
2	27.053	MM	2.2883	4127.20117	30.06008	5.6978

Peak #	RetTime [min]	Type	Width [min]	Area [mAU*s]	Height [mAU]	Area %
3	33.921	MM	3.0690	2.33060e4	126.56712	32.1752
4	101.253	MM	9.4512	4.17452e4	73.61555	57.6317

Totals :		7.24345e4	255.49164

Figure S-66 HPLC trace of (*S*)-10k

1-tert-butyl 3-ethyl 2-((*R*)-2-nitro-1-phenylethyl)malonate ((*R*)-10k):

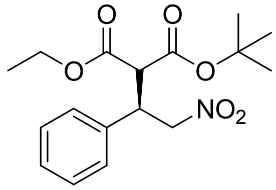

Λ-1 used as catalyst and (*R*)**-10k** was obtained as a mixture of inseparable diastereomers.

Analytical data for title compound: (24 mg, 39%, dr~50:50) , $[\alpha]_D^{25}$ = -4.7 (*c* 0.44, $CHCl_3$);

HPLC (Chiralcel AD-H, hexane/isopropanol= 93/7, 0.8 mL/min, λ= 210 nm, retention times:

First diastereon [illegible] % ee;

Second diastere [illegible] 24% ee

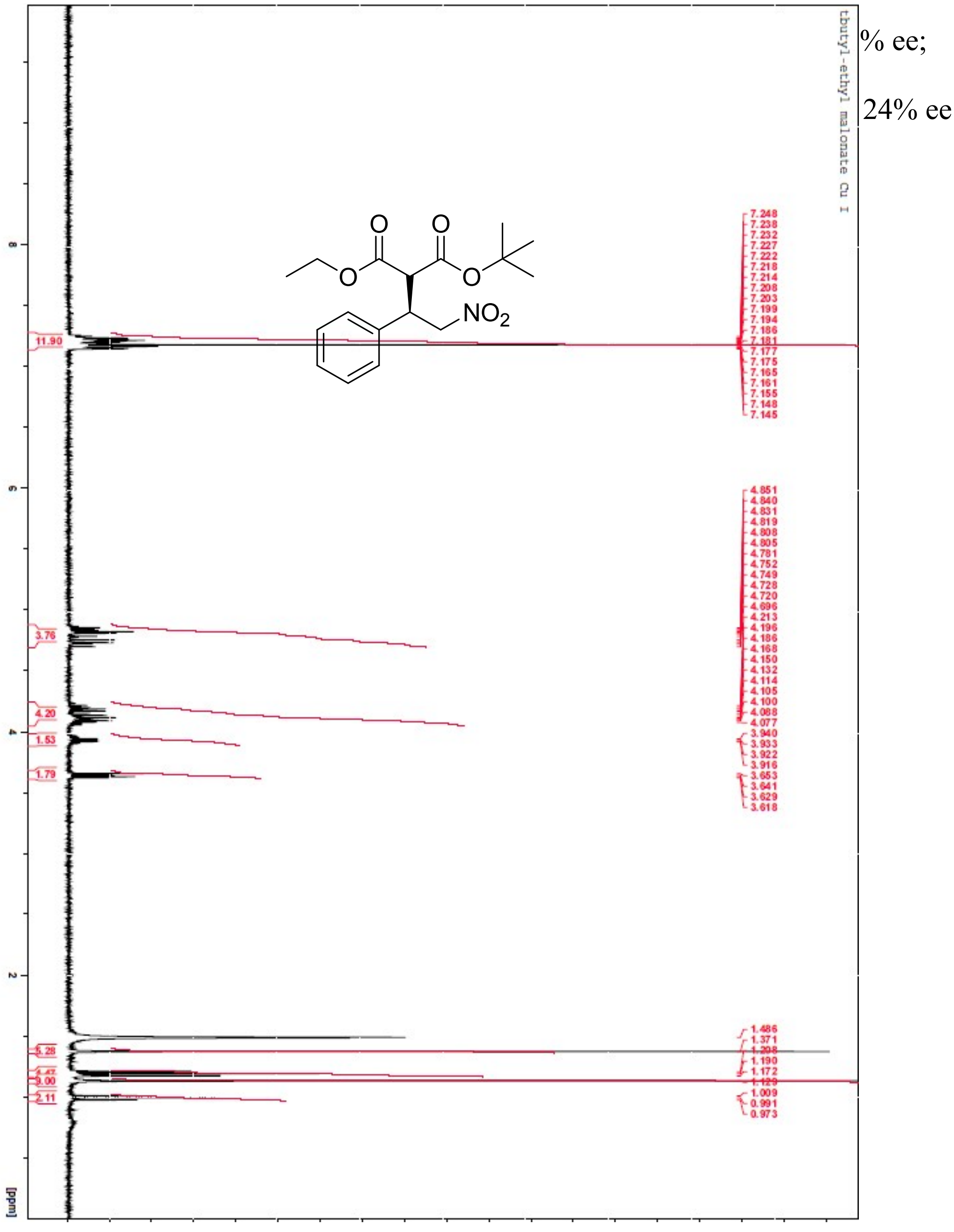

Figure S-67 ^{1}H-NMR of (*R*)-10k

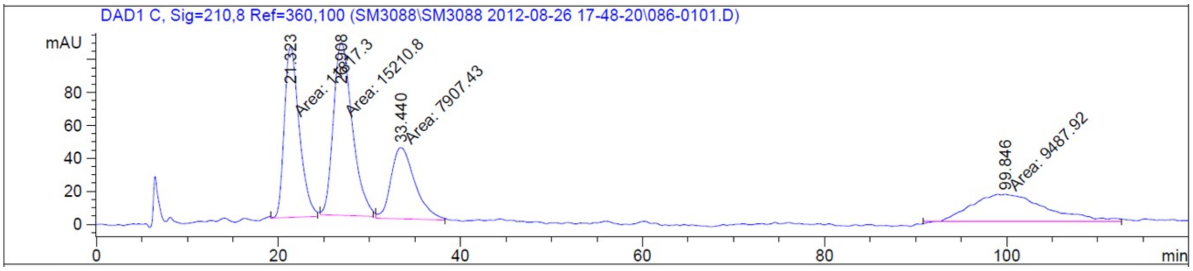

Signal 2: DAD1 C, Sig=210,8 Ref=360,100

Peak #	RetTime [min]	Type	Width [min]	Area [mAU*s]	Height [mAU]	Area %
1	21.323	MM	1.9000	1.18173e4	103.65935	26.6015
2	26.908	MM	2.4155	1.52108e4	104.95490	34.2405

Peak #	RetTime [min]	Type	Width [min]	Area [mAU*s]	Height [mAU]	Area %
3	33.440	MM	3.0485	7907.42529	43.23138	17.8001
4	99.846	MM	9.6059	9487.92285	16.46194	21.3579

Totals : 4.44235e4 268.30756

Figure S-68 HPLC trace of (*R*)-10k

1-tert-butyl 3-methyl 2-((*S*)-2-nitro-1-phenylethyl)malonate ((*S*)-10i):

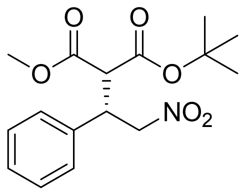

Δ-1 used as catalyst and (*S*)-**10i** was obtained as a mixture of inseparable diastereomers.

Analytical data for title compound: (28 mg, 51%, dr~70:30) , $[\alpha]_D^{25}$ = -1.4 (*c* 1.03, $CHCl_3$); ^{1}H NMR (400 MHz, $CDCl_3$) δ 7.25-7.14 (m, 11H), 4.83-4.67 (m, 3.6H), 4.12-4.06 (m, 1.8H), 3.69-3.64 (m, 3.5H), 3.49 (m, 1.8H), 1.36 (s, 5.2H), 1.17 (s, 9H), ^{13}C NMR (100 MHz, $CDCl_3$) δ 168.4, 165.6, 136.3, 128.9, 128.8, 128.2, 127.9, 77.2, 55.8, 55.5, 52.8, 43.2, 27.8, 27.4, HRMS (EI) calc. for $[C_{17}H_{23}NNaO_6]^+$ 346.1267, found 360.1243; HPLC (Chiralcel OJ-H, hexane/isopropanol= 95/5, 1.0 mL/min, λ= 210 nm, retention times: First diastereomer: major enantiomer t_r = 19.5 min, minor enantiomer t_r = 25.3 min; 60% ee; Second diastereomer: major enantiomer t_r = 21.4 min, minor enantiomer t_r = 29.1 min; 73% ee

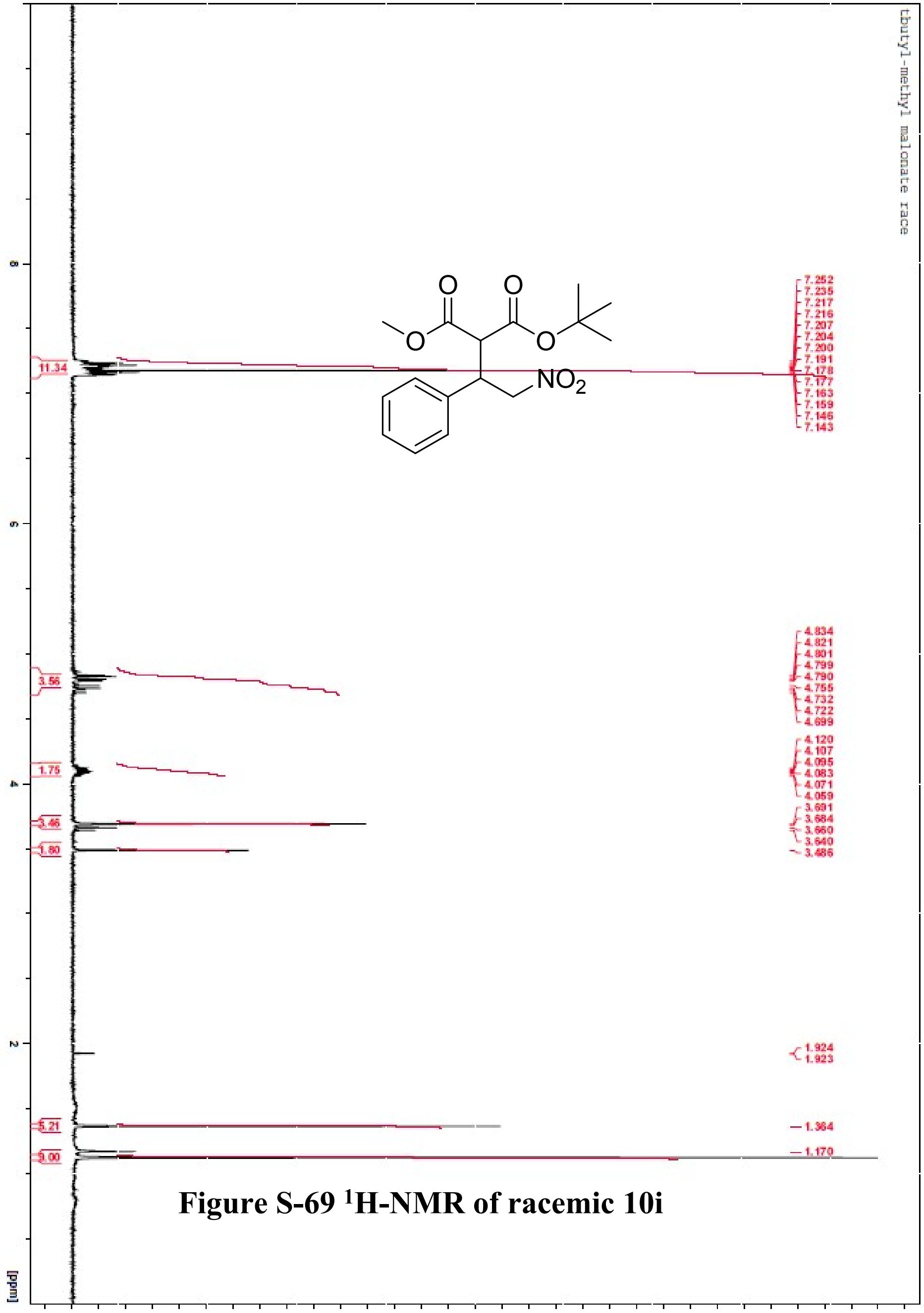

Figure S-69 ^{1}H-NMR of racemic 10i

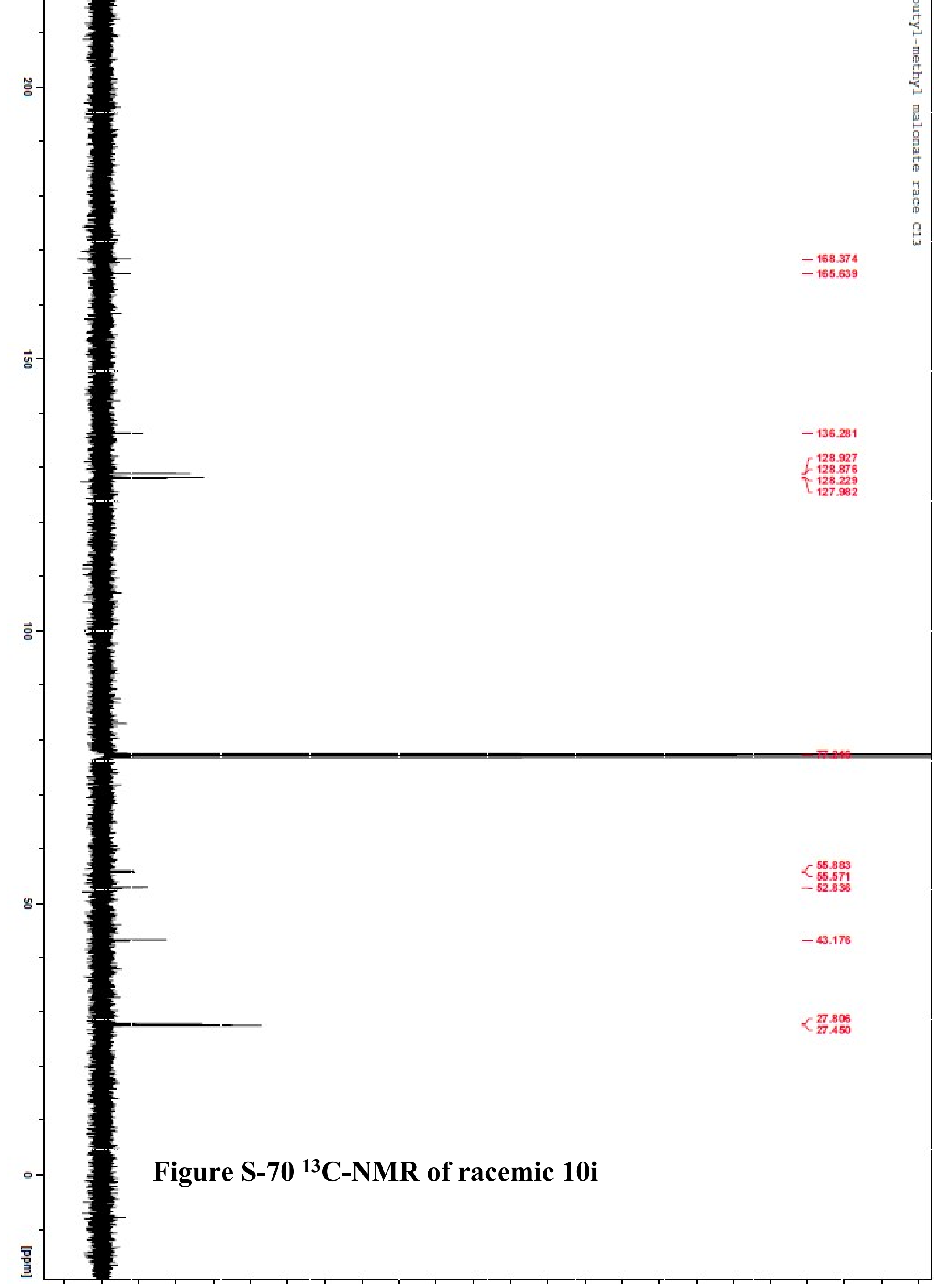

Figure S-70 ^{13}C-NMR of racemic 10i

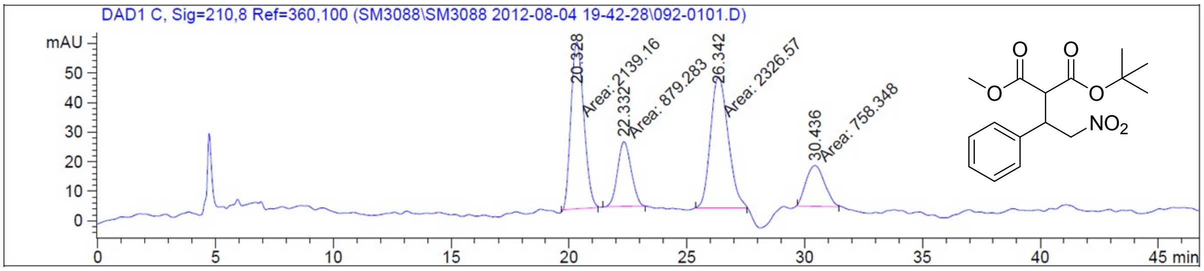

Signal 2: DAD1 C, Sig=210,8 Ref=360,100

Peak #	RetTime [min]	Type	Width [min]	Area [mAU*s]	Height [mAU]	Area %
1	20.328	MM	0.6316	2139.16284	56.44912	35.0489
2	22.332	MM	0.6702	879.28314	21.86770	14.4065

Peak #	RetTime [min]	Type	Width [min]	Area [mAU*s]	Height [mAU]	Area %
3	26.342	MM	0.8722	2326.57104	44.45645	38.1195
4	30.436	MM	0.9151	758.34802	13.81130	12.4251

Totals : 6103.36505 136.58457

Figure S-71 HPLC trace of racemic 10i

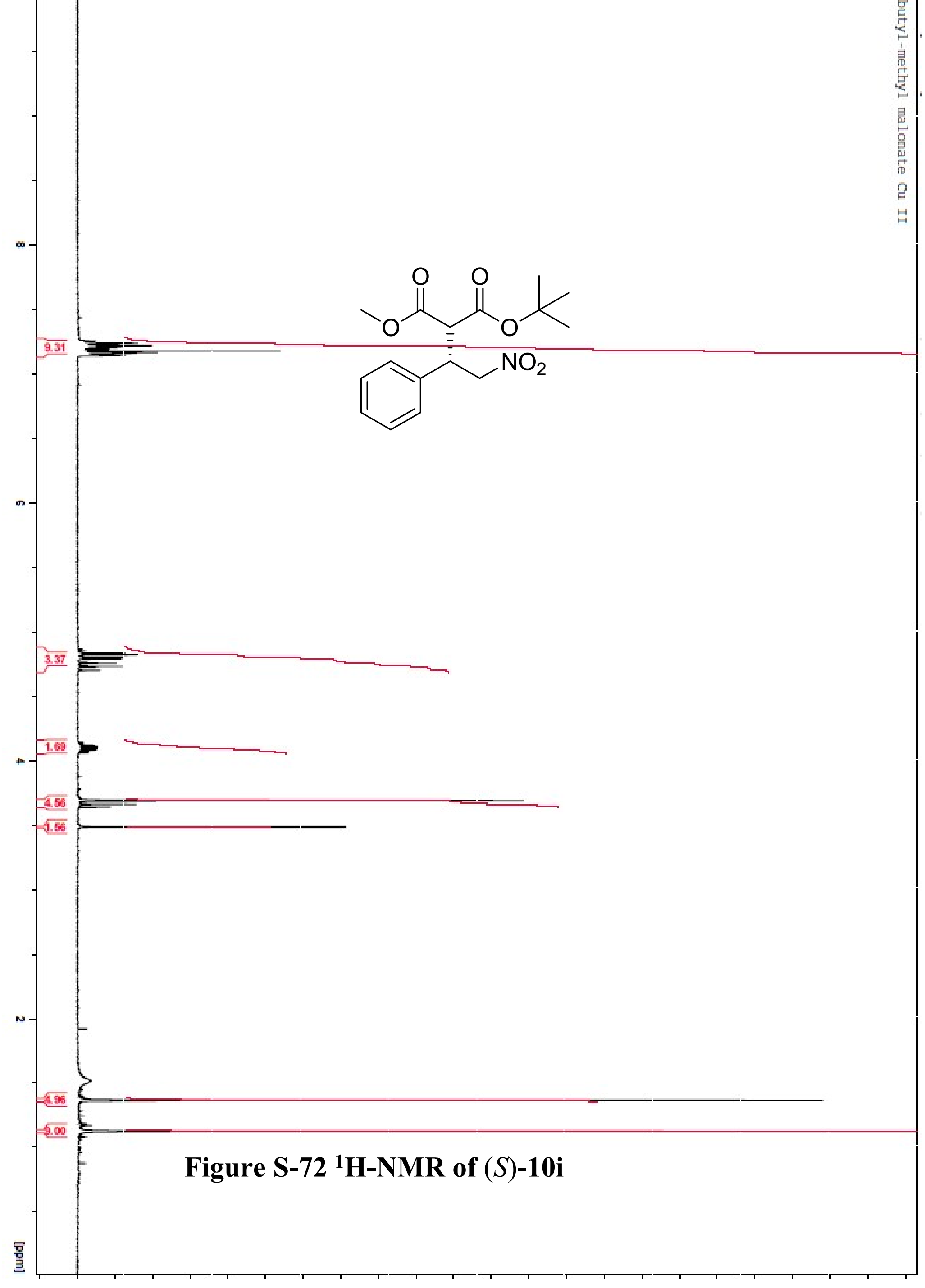

Figure S-72 ^{1}H-NMR of (*S*)-10i

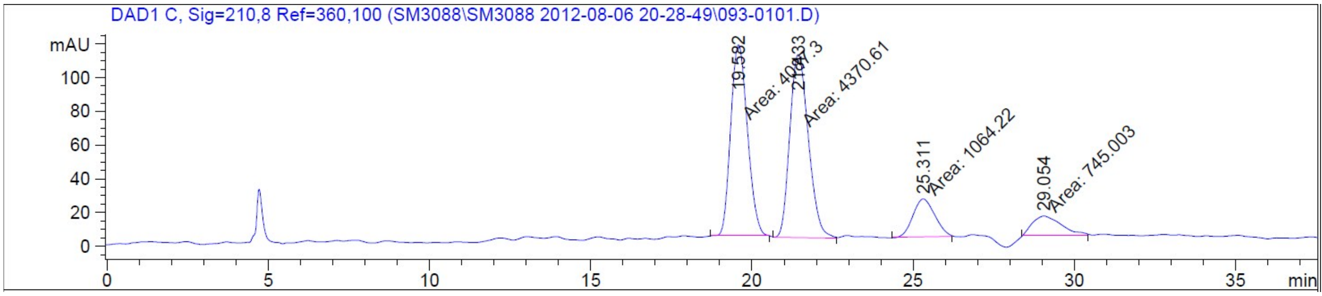

Signal 2: DAD1 C, Sig=210,8 Ref=360,100

Peak #	RetTime [min]	Type	Width [min]	Area [mAU*s]	Height [mAU]	Area %
1	19.582	MM	0.6000	4087.29663	113.53278	39.8095
2	21.433	MM	0.6694	4370.60645	108.82658	42.5689

Peak #	RetTime [min]	Type	Width [min]	Area [mAU*s]	Height [mAU]	Area %
3	25.311	MM	0.7858	1064.22400	22.57256	10.3654
4	29.054	MM	1.0619	745.00299	11.69344	7.2562

Totals : 1.02671e4 256.62536

Figure S-73 HPLC trace of (*S*)-10i

1-tert-butyl 3-methyl 2-((*R*)-2-nitro-1-phenylethyl)malonate ((*R*)-10i):

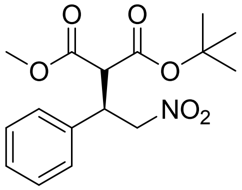

Λ-1 used as catalyst and (*R*)**-10i** was obtained as a mixture of inseparable diastereomers. Analytical data for title compound: (25 mg, 40%, dr~68:32) , $[\alpha]_D^{25}$ = -4.9 (*c* 0.44, $CHCl_3$, 72% *ee* (S)); HPLC (Chiralcel OJ-H, hexane/isopropanol= 95/5, 1.0 mL/min, λ= 210 nm, retention times: First diastereomer: minor enantiomer t_r = 18.3 min, major enantiomer t_r = 23.5 min; 64% ee; Second dia[illegible] min; 64% ee

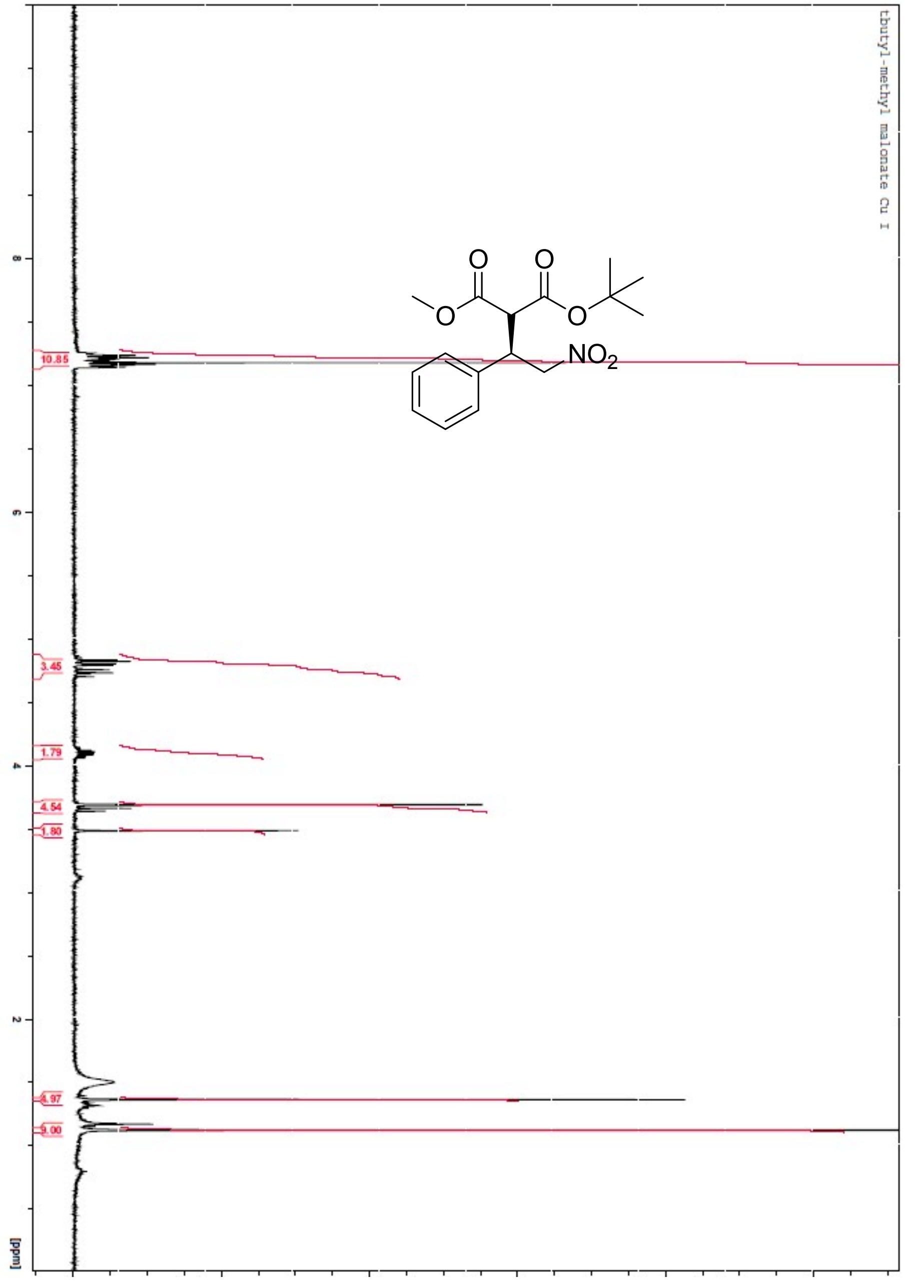

Figure S-74 ^{1}H-NMR of (*R*)-10i

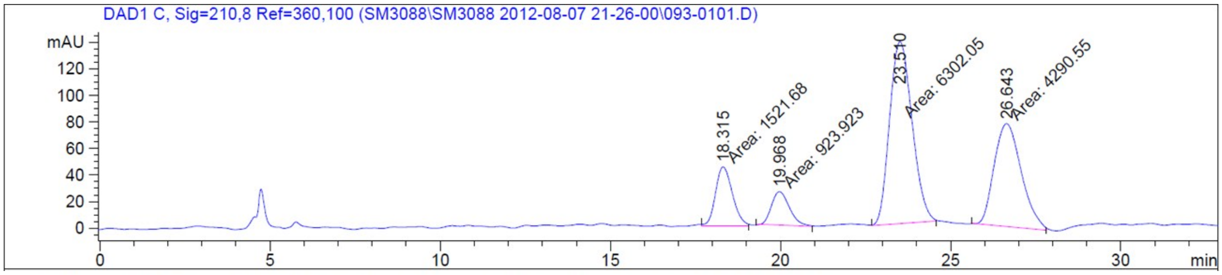

Signal 2: DAD1 C, Sig=210,8 Ref=360,100

Peak #	RetTime [min]	Type	Width [min]	Area [mAU*s]	Height [mAU]	Area %
1	18.315	MM	0.5720	1521.68201	44.33887	11.6709
2	19.968	MM	0.6121	923.92297	25.15882	7.0863

Peak #	RetTime [min]	Type	Width [min]	Area [mAU*s]	Height [mAU]	Area %
3	23.510	MM	0.7662	6302.05176	137.08719	48.3353
4	26.643	MM	0.9234	4290.54590	77.44334	32.9075

Totals :				1.30382e4	284.02822	

Figure S-75 HPLC trace of (*R*)-10i

Λ-1 by reduction of **Δ-1**: Catalyst **Δ-1** (24 mg, 0.017 mmol) was dissolved in 1 mL of CH_2Cl_2. NEt_3 (4 μL, 0.034 mmol) and L-ascorbic acid (6 mg, 0.034 mmol) were then added to the mixture and stirred for 1 h at room temperature. A color change from light blue to yellow was observed after addition of ascorbic acid. The solvent was removed *in vacuo*, and the solid was suspended in degassed H_2O (2 mL) and filtered. The solid residue was washed with H_2O and hexane before drying under reduced pressure. The yellow solid was then used as catalyst.

III. Method for *in situ* Catalysis

Ligand **7** (18 mg, 0.018 mmol), $Cu^{II}(ClO_4)_2$● $6(H_2O)$ (6.4 mg, 0.018 mmol), 10 mg of 4 Å MS, and 1 mL of CH_3CN were added to a scintillation vial and stirred for 1 h before nitrostyrene (**8**) (50 mg, 0.34 mmol), diethyl malonate (**9**) (102 μL, 0.67 mmol), and NEt_3 (4 μL, 0.034 mmol) were added to the vial. The reaction was stirred at room temperature for 1 h and solvent was removed. Crude product was purified using silica column chromatography (90/10 hexane/ethyl acetate) to obtain (*S*)-**10** (66 mg, 63%) in 72% ee.

For *in situ* formation of **Λ-1**, $Cu(MeCN)_4PF_6$ was used as the copper salt

IV. Crystallographic information for Δ-1

Single Crystal Structure Determination Experimental Description

A green block-like crystal with the size of 0.30 × 0.34 × 0.50 mm^3 was selected for geometry and intensity data collection with a Bruker SMART APEXII CCD area detector on a D8 goniometer at 100 K. The temperature during the data collection was controlled with an Oxford Cryosystems Series 700+ instrument. Preliminary lattice parameters and orientation matrices were obtained from three sets of frames. Data were collected using graphite-monochromated and 0.5 mm-MonoCap-collimated Mo-K_α radiation (λ = 0.71073 Å) with the ω scan method.(2) Data were processed with the INTEGRATE program of the APEX2 software(2) for reduction and cell refinement. Multi-scan absorption corrections were applied by using the SCALE program for the area detector. The structure was solved by the direct method and refined on F^2 (SHELXTL).(3) Non-hydrogen atoms, except a highly disordered 3,5-bis(trifluoromethyl)benzene group, were refined with anisotropic displacement parameters, and hydrogen atoms on carbons and nitrogens were placed in idealized positions (C-H = 0.95-1.00 Å and N-H = 0.88 Å) and included as riding with U_{iso}(H) = 1.2 or 1.5 U_{eq}(non-H).

Table 1. Sample and crystal data for Δ-1.

**Identification code**	13jca3h	
**Chemical formula**	$C_{113.30}H_{88.95}Cl_2Cu_2F_{24}N_{15.65}O_{14}S_2$	
**Formula weight**	2611.73	
**Temperature**	100(2) K	
**Wavelength**	0.71073 Å	
**Crystal size**	0.300 x 0.340 x 0.500 mm	
**Crystal habit**	green block	
**Crystal system**	trigonal	
**Space group**	P 32 2 1	
**Unit cell dimensions**	a = 29.930(6) Å	α = 90°
	b = 29.930(6) Å	β = 90°
	c = 28.878(6) Å	γ = 120°
**Volume**	22403.(8) $Å^3$	
**Z**	6	
**Density (calculated)**	1.161 g/cm^3	
**Absorption coefficient**	0.432 mm^{-1}	
**F(000)**	8353	

Table 2. Data collection and structure refinement for Δ-1.

**Diffractometer**	Bruker APEX-II CCD	
**Radiation source**	sealed tube, MoKα	
**Theta range for data collection**	1.36 to 23.00°	
**Index ranges**	-32<=h<=32, -32<=k<=32, -31<=l<=31	
**Reflections collected**	163141	
**Independent reflections**	20779 [R(int) = 0.0863]	
**Absorption correction**	multi-scan	
**Max. and min. transmission**	0.8813 and 0.8129	
**Structure solution technique**	direct methods	
**Structure solution program**	SHELXS-97 (Sheldrick, 2008)	
**Refinement method**	Full-matrix least-squares on F^2	
**Refinement program**	SHELXL-97 (Sheldrick, 2008)	
**Function minimized**	$\Sigma w(F_o^2 - F_c^2)^2$	
**Data / restraints / parameters**	20779 / 305 / 1393	
**Goodness-of-fit on F^2**	1.005	
**Final R indices**	15401 data; $I>2\sigma(I)$	R1 = 0.0782, wR2 = 0.2086
	all data	R1 = 0.0974, wR2 = 0.2228
**Weighting scheme**	$w=1/[\sigma^2(F_o^2)+(0.1611P)^2]$ where $P=(F_o^2+2F_c^2)/3$	
**Absolute structure parameter**	0.0(1)	
**Largest diff. peak and hole**	0.661 and -0.481 eÅ^{-3}	
**R.M.S. deviation from mean**	0.086 eÅ^{-3}	

V. References

(1) Evans, D. A.; Mito, S.; Seidel, D. *Journal of the American Chemical Society* **2007**, *129*, 11583.

(2) APEX2 (version 2012.10). *Program for Bruker CCD X-ray Diffractometer Control*, Bruker AXS Inc., Madison, WI, 2012.

(3) G. M. Sheldrick, SHELXTL, version 6.14. *Program for solution and refinement of crystal structures*, Universität Göttingen, Germany, 2009.
